# A nearly complete skull of the sauropod dinosaur *Diamantinasaurus matildae* from the Upper Cretaceous Winton Formation of Australia and implications for the early evolution of titanosaurs

**DOI:** 10.1098/rsos.221618

**Published:** 2023-04-12

**Authors:** Stephen F. Poropat, Philip D. Mannion, Samantha L. Rigby, Ruairidh J. Duncan, Adele H. Pentland, Joseph J. Bevitt, Trish Sloan, David A. Elliott

**Affiliations:** ^1^ Western Australian Organic and Isotope Geochemistry Centre, School of Earth and Planetary Science, Curtin University, Bentley, Western Australia 6102, Australia; ^2^ Australian Age of Dinosaurs Museum of Natural History, Winton, Queensland 4735, Australia; ^3^ School of Biological Sciences, Monash University, Clayton, Victoria 3800, Australia; ^4^ Department of Earth Sciences, University College London, Gower Street, London WC1E 6BT, UK; ^5^ School of Science, Computing and Engineering Technologies, Swinburne University of Technology, Hawthorn, Victoria 3122, Australia; ^6^ Australian Centre for Neutron Scattering, Australian Nuclear Science and Technology Organisation, Sydney, New South Wales 2234, Australia

**Keywords:** Dinosauria, Sauropoda, Titanosauria, Gondwana, Australia

## Abstract

Titanosaurian sauropod dinosaurs were diverse and abundant throughout the Cretaceous, with a global distribution. However, few titanosaurian taxa are represented by multiple skeletons, let alone skulls. *Diamantinasaurus matildae*, from the lower Upper Cretaceous Winton Formation of Queensland, Australia, was heretofore represented by three specimens, including one that preserves a braincase and several other cranial elements. Herein, we describe a fourth specimen of *Diamantinasaurus matildae* that preserves a more complete skull—including numerous cranial elements not previously known for this taxon—as well as a partial postcranial skeleton. The skull of *Diamantinasaurus matildae* shows many similarities to that of the coeval *Sarmientosaurus musacchioi* from Argentina (e.g. quadratojugal with posterior tongue-like process; braincase with more than one ossified exit for cranial nerve V; compressed-cone–chisel-like teeth), providing further support for the inclusion of both taxa within the clade Diamantinasauria. The replacement teeth within the premaxilla of the new specimen are morphologically congruent with teeth previously attributed to *Diamantinasaurus matildae*, and Diamantinasauria more broadly, corroborating those referrals. Plesiomorphic characters of the new specimen include a sacrum comprising five vertebrae (also newly demonstrated in the holotype of *Diamantinasaurus matildae*), rather than the six or more that typify other titanosaurs. However, we demonstrate that there have been a number of independent acquisitions of a six-vertebrae sacrum among Somphospondyli and/or that there have been numerous reversals to a five-vertebrae sacrum, suggesting that sacral count is relatively plastic. Other newly identified plesiomorphic features include: the overall skull shape, which is more similar to brachiosaurids than ‘derived' titanosaurs; anterior caudal centra that are amphicoelous, rather than procoelous; and a pedal phalangeal formula estimated as 2-2-3-2-0. These features are consistent with either an early-branching position within Titanosauria, or a position just outside the titanosaurian radiation, for Diamantinasauria, as indicated by alternative character weighting approaches applied in our phylogenetic analyses, and help to shed light on the early assembly of titanosaurian anatomy that has until now been obscured by a poor fossil record.

## 1. Introduction

The early evolution of titanosaurian sauropod dinosaurs remains poorly understood [1,2], despite the fact that the fossil record of the clade spans much of the Cretaceous [3–7] and includes exemplars from every continent (e.g. [8–15]). One of the greatest impediments to resolving titanosaur phylogenetics has been the dearth of cranial remains. Several of the best known titanosaur skulls date to the latest Cretaceous, namely those of *Rapetosaurus krausei* from Madagascar [16,17], and *Nemegtosaurus mongoliensis* and *Quaesitosaurus orientalis* from Mongolia [18–20]. However, of critical importance to understanding the early evolution of Titanosauria has been the recent description of two titanosaur skulls from the mid-Cretaceous of South America: *Tapuiasaurus macedoi* from the Aptian of Brazil [21,22], and *Sarmientosaurus musacchioi* from the Cenomanian–Turonian of Argentina [23]. The skull of *Tapuiasaurus* is strikingly similar to those of the substantially geochronologically younger titanosaurs of Madagascar and Mongolia [21]. This implies either marked cranial morphological conservatism across a vast spatiotemporal interval, or convergent evolution. By contrast, the skull of *Sarmientosaurus* appears to be somewhat intermediate in morphology between earlier-deriving titanosauriforms such as brachiosaurids (e.g. ‘*Brachiosaurus*' sp. [24]; *Giraffatitan brancai* [25]; *Abydosaurus mcintoshi* [26]), and most other titanosaurs known from substantial cranial remains [23]. Thus, *Sarmientosaurus* more fully embodies the cranial morphology perhaps expected of an early-branching titanosaur than does *Tapuiasaurus*, despite the latter taxon being geochronologically older. Unfortunately, very little is known of the postcranial anatomy of *Sarmientosaurus*, making it difficult to constrain its phylogenetic relationships [23] and leaving it susceptible to ‘monophyly of the preserved' branch attraction effects (see [5]).

Although Cretaceous sedimentary rocks in northeast Australia have produced abundant evidence of sauropod dinosaurs [9,27–44], very few cranial elements have been discovered. Indeed, aside from teeth and a dentary fragment [33,35,43], only one partial skull has been described to date: that of a referred specimen of *Diamantinasaurus matildae* (AODF 0836), which preserves a braincase and several additional cranial remains [38,41]. Numerous similarities between the cranial remains and teeth of *Diamantinasaurus* and *Sarmientosaurus* have been identified, and a close relationship between these taxa, within the early-branching titanosaurian clade Diamantinasauria, has been supported by phylogenetic analyses [41]. Nevertheless, the overall morphology of the skull of *Diamantinasaurus* remains unclear, as does the position of the clade Diamantinasauria within Titanosauria [1,45].

In this paper, we describe a new specimen of *Diamantinasaurus matildae*, comprising a partial skull with associated postcrania from the lower Upper Cretaceous Winton Formation of Queensland, northeast Australia. We compare this specimen with other sauropods, assess its phylogenetic position, and discuss its evolutionary implications.

### 1.1. Institutional abbreviations

AODF, Australian Age of Dinosaurs Museum Fossil, Winton, Australia; AODL, Australian Age of Dinosaurs Museum Locality, Winton, Australia; ASDM, Arizona-Sonora Desert Museum, Tucson, USA; CAMSM, Sedgwick Museum of Earth Sciences, University of Cambridge, England; CPT, Museo Aragonés de Paleontología, Fundación Conjunto Paleontológico de Teruel-Dinópolis, Teruel, Spain; DMNH, Denver Museum of Natural History, Denver, USA; DNHM, Dalian Natural History Museum, Liaoning, China; FC-DPV, Colección de Vertebrados Fósiles, Facultad de Ciencias, Universidad de la República, Uruguay; FMNH PR, Field Museum of Natural History, Chicago, USA; FPDM, Fukui Prefectural Dinosaur Museum, Fukui, Japan; FWMSH, Fort Worth Museum of Science and History, Fort Worth, USA; GSI, Geological Survey of India, Kolkata, India; IANIGLA-PV, Instituto Argentino de Nivología, Glaciología y Ciencias Ambientales, Colección de Paleovertebrados, Mendoza, Argentina; IVPP, Institute of Vertebrate Paleontology and Paleoanthropology, Chinese Academy of Sciences, Beijing, China; IZANUZ, Institute of Zoology, Uzbekistan Academy of Sciences, Tashkent, Uzbekistan; MACN PV, Museo Argentino de Ciencias Naturales ‘Bernardino Rivadavia', Buenos Aires, Argentina; MAL, Malawi Department of Antiquities Collection, Lilongwe and Nguludi, Malawi; MAU, Museo Municipal ‘Argentina Urquiza', Rincón de los Sauces, Argentina; MCF-PVPH, Museo Carmen Funes, Plaza Huincul, Neuquén, Argentina; MCNA, Museo de Ciencias Naturales de Álava/Arabako Natur Zientzien Museoa, Vitoria-Gasteiz, Spain; MCS, Museo de Cinco Saltos, Río Negro, Argentina; MDE, Le Musée des Dinosaures in Espéraza, France; MLP Av., Museo de La Plata, Rancho de Ávila Collection, La Plata, Argentina; MLP CS, Museo de La Plata, Cinco Saltos Collection, La Plata, Argentina; MMS/VBN, Musée Moulin Seigneurial/Velaux-La Bastide Neuve, Bouches-du-Rhône, France; MPCA-Pv, Museo Provincial ‘Carlos Ameghino', Cipolletti, Río Negro, Argentina; MPEF-PV, Museo Paleontológico Egidio Feruglio, Trelew, Argentina; MPM, Museu de Paleontologia de Marília, Marília, São Paulo, Brazil; MPM PV, Museo Padre Molina, Río Gallegos, Argentina; MPZ, Museo Paleontológico de la Universidad de Zaragoza, Zaragoza, Spain; MRS Pv, Museo de Rincón de los Sauces, Neuquén, Argentina; MUCPv, Museo de Geología y Paleontología de la Universidad Nacional del Comahue, Argentina; MUVP, Mansoura University Vertebrate Paleontology Center, Mansoura University, Egypt; MZSP-PV, Museu de Zoologia da Universidade de São Paulo, Brazil; NMMNH, New Mexico Museum of Natural History and Science, Albuquerque, USA; PMU, Paleontological Museum of the University of Uppsala, Uppsala, Sweden; PVL, Fundacion Miguel Lillo, Universidad Nacional de Tucuman, San Miguel de Tucuman, Argentina; QM, Queensland Museum (Brisbane, Australia); RRBP, Rukwa Rift Basin Project, Tanzanian Antiquities Unit, Dar es Salaam, Tanzania; SM, Sirindhorn Museum, Changwat Kalasin, Thailand; TMM, Texas Memorial Museum, University of Texas, Austin, USA; TV, Musée des Dinosaures à Savannakhet, Laos; UA, Université d'Antananarivo, Antananarivo, Madagascar; UBB NVM1, Babes-Bolyai University, Cluj-Napoca, Romania; UNCUYO-LD, Universidad Nacional de Cuyo, Mendoza, Argentina; UNPSJB-PV, Universidad Nacional de la Patagonia ‘San Juan Bosco'–Paleovertebrados, Comodoro Rivadavia, Argentina; USNM, National Museum of Natural History, Washington, DC, USA; Z. PAL, Instytut of Paleobiologii, Polish Academy of Sciences, Warsaw, Poland; ZIN PH, Paleoherpetological Collection, Zoological Institute, Russian Academy of Sciences, Saint Petersburg, Russia.

## 2. Geological setting

**Figure 1.**
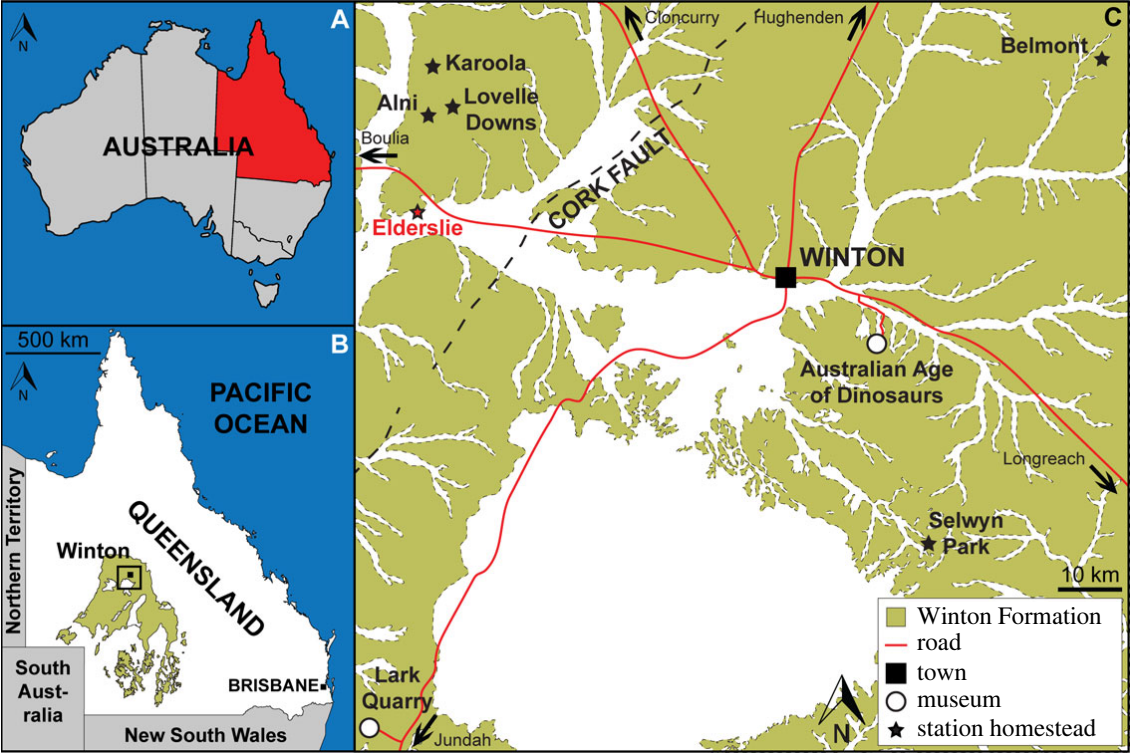
Location of the ‘Ann’ Site (AODL 0252). (*a*) Map of Australia showing the location of Queensland. (*b*) Map of Queensland showing the distribution of Winton Formation outcrop. (*c*) Map of the Winton area showing the extent of Winton Formation outcrop, the location of Elderslie Station and numerous other stock stations and sites in the area from which sauropod fossils have been collected. Map drafted by the senior author (S.F.P.) in Adobe Illustrator CC 2022 (modified from Poropat *et al*. [43], incorporating geological information from Vine [46] and Vine and Casey [47] (© Commonwealth of Australia (Geoscience Australia) 2022. This product is released under the Creative Commons Attribution 4.0 International Licence. http://creativecommons.org/licenses/by/4.0/legalcode).

The Winton Formation (figure 1) is the stratigraphically uppermost Mesozoic-aged sedimentary unit in the Eromanga Basin of northeast Australia [48]. The mudstones, siltstones, sandstones and minor conglomerates that make up this unit were deposited on a low relief, forested floodplain [49], shortly after the recession of the epeiric Eromanga Sea [50,51]. The top 100 m of the Winton Formation (and the underlying Mackunda Formation) was subjected to substantial chemical weathering during the Cretaceous and early Cenozoic, following cessation of sedimentation in the Eromanga Basin [48]. Consequently, despite its great lateral extent, Winton Formation outcrop is limited to nodular sandstones, as well as erosion-resistant mesas comprising chemically weathered siltstones and sandstones [51]. The remaining Winton Formation is now blanketed by montmorillonite-rich soil [48].

Vertebrates are represented in the Winton Formation by both body fossils and ichnofossils. Actinopterygians are represented by the ichthyodectiform *Cladocyclus geddesi* from near Isisford [52], as well as a possible feeding trace from near Winton [42]. Isolated toothplates of two ceratodontoid dipnoan species (*Metaceratodus wollastoni* and *M. ellioti*) are known from several widely dispersed localities, [53–55], and possible lungfish feeding traces have also been identified near Winton [42]. Lissamphibians and synapsids are as yet unknown from the Winton Formation, whereas lepidosauromorphs are represented by a single vertebra originally interpreted as a dolichosaurid [56], but more recently regarded as an indeterminate varanoid [57]. Although fossil remains of testudinatans (possibly chelids) are seemingly fairly common near both Winton and Eromanga [9,34], the only described evidence for their presence constitutes possible ichnites from near Winton [42]. Crocodyliforms are well represented near both Isisford and Winton, with the former region producing *Isisfordia duncani* [58,59], and the latter producing *Confractosuchus sauroktonos* [60], as well as several trackways made by swimming or underwater-walking crocodyliforms [42]. Pterosaurs are rare, with the anhanguerid *Ferrodraco lentoni* and an isolated anhanguerian femur from near Winton being the only specimens described to date [61–63]. Ankylosaurian dinosaurs are also uncommon, the only published specimens constituting three teeth from near Winton [64]. The ornithopod body fossil record is restricted to a single tooth from near Winton [65,66], a partial skeleton found within the body cavity of *Confractosuchus sauroktonos* [60], and undescribed specimens from near Isisford [67]. Conversely, the ornithopod ichnofossil record is extensive, with both Lark Quarry and the Snake Creek Tracksite providing evidence of small- to medium-sized ornithopods [42,68–75]. Theropod teeth are fairly commonly found at vertebrate sites near Winton [76], but other body fossils are presently limited to the holotype specimen of the megaraptorid *Australovenator wintonensis* [9,76–78] and an indeterminate megaraptorid [79]. Tracks possibly made by theropods are also fairly common near Winton [42,68–70], although their attribution to theropod trackmakers has been the subject of some debate [42,71–75,80]. Sauropods are common as body fossils in the Winton Formation [28–31,33,42], with four taxa described to date: the early-branching somphospondylan (or perhaps diamantinasaurian titanosaur [34]) *Wintonotitan wattsi* [9,36] and the diamantinasaurian titanosaurs *Diamantinasaurus matildae* [9,37,38,41,44] and *Savannasaurus elliottorum* [38,40] from near Winton, and the diamantinasaurian titanosaur *Australotitan cooperensis* from near Eromanga [34]. Sauropod tracks have also been reported from both Winton [42] and Eromanga [34].

**Figure 2.**
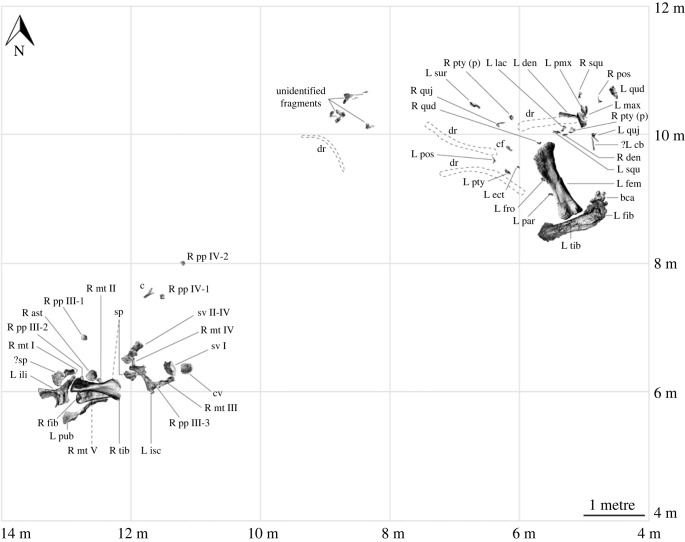
*Diamantinasaurus matildae* referred specimen (AODF 0906) site map. Site map showing the distribution of skeletal elements of the newly referred *Diamantinasaurus matildae* specimen (AODF 0906) within the ‘Ann’ Site (AODL 0252). ast, astragalus; bca, braincase; c, chevron; cb, ceratobranchial; cf, cervical fragment; cv, caudal vertebra; den, dentary; dr, dorsal rib; ect, ectopterygoid; fem, femur; fib, fibula; fro, frontal; ili, ilium; isc, ischium; L, left; lac, lacrimal; max, maxilla; mt ##, metatarsal and position; (p), part; par, parietal; pmx, premaxilla; pos, postorbital; pp ##-#, pedal phalanx and position; pty, pterygoid; pub, pubis; qud, quadrate; quj, quadratojugal; R, right; sp, sacral process; squ, squamosal; sur, surangular; sv #, sacral vertebra and position; tib, tibia.

The new sauropod site, AODL 0252 (the ‘Ann' Site), is approximately 10 m × 6 m in area, but is divisible into two concentrated sections (one west and one east), between which few fragments of bone were found (figure 2). The western section of AODL 0252, which is about 3 m × 3 m, has yielded a partial sacrum, an anterior caudal vertebra, a chevron, the left ilium, the left pubis, both ischia, and much of the right hind limb including the femur, tibia, fibula, metatarsals I–V, and several pedal phalanges including at least one ungual. Thus, the western section preserves elements restricted to the posterior half of the animal. The proximal end of the left pubis, and the right femur, tibia and fibula were found in the ‘black soil' layer that represents the weathered cap of the Winton Formation. The remaining bones were found below the ‘black soil' in the Winton Formation proper. The long axes of the left pubis, right tibia and right fibula were effectively parallel. Four of the five metatarsals and the probable astragalus fragment were found immediately beneath the tibia and fibula, and the fifth was found approximately 50 cm northeast, associated with fragmentary sacral vertebrae. The sole chevron and two other pedal phalanges were found 1–1.5 m northeast of this concentration of bones. Thus, all of these remains were found in close association.

The eastern section of AODL 0252, which is also about 3 m × 3 m, yielded all of the sauropod cranial remains recovered from this site. The skull was almost entirely disarticulated, and its various elements were scattered around a partial left hind limb (comprising the femur, tibia and fibula) and several partial dorsal ribs. The left tibia and fibula were probably articulated prior to the incorporation of the former into the ‘black soil’, and both were found at the distal end of the left femur, with their long axes perpendicular to its long axis. The braincase was found near the proximal end of the left fibula, and several additional elements (including the left frontal, left parietal and a fragment of the right quadrate) were found scattered within the lowest sedimentary level occupied by the femur (an approx. 10 cm thick layer of siltstone, blue–grey when unweathered). Three partial dentigerous elements, all from the left side, were found near the proximal end of the femur. The left premaxilla was found wedged within the antorbital foramen of the maxilla, whereas the dentary was found in contact with, and with its long axis perpendicular to that of, the dentigerous portion of the maxilla. The remaining cranial elements were found in the same horizon, within 1.5 m of the proximal end of the femur.

All of the sauropod elements found within AODL 0252 are size congruent (notably the hind limb elements from the western and eastern sections), some articulate perfectly with one another, and there is no duplication of elements. Moreover, there is no evidence of other dinosaurs at the site, and little evidence of other animals (beyond a few possible turtle and fish fragments). Thus, we interpret all of the sauropod remains from AODL 0252 as deriving from a single individual. Based on measurements of overlapping elements, the sauropod at AODL 0252 was approximately the same size as, or perhaps slightly larger than, the *Diamantinasaurus matildae* holotype specimen (AODF 0603); however, it appears to have been more gracile based on the dimensions of the hind limb elements. Based on the size of the skull elements (e.g. quadrate dorsoventral height 216 mm), the AODF 0906 skull represents a slightly larger individual than AODF 0836 (quadrate dorsoventral height 184 mm).

## 3. Methods

Permission to dig on Elderslie Station was granted to the Australian Age of Dinosaurs Museum of Natural History by the landowners.

The cranial remains of AODF 0906 were CT scanned at St Vincent's Hospital (Melbourne, Victoria). One objective of this was to determine whether or not any replacement teeth were present within the dentigerous elements. As several were identified in the premaxilla, this element was subsequently scanned at the Australian Nuclear Science and Technology Organisation's (ANSTO) Australian Synchrotron (Melbourne, Australia) to provide higher-resolution imagery. Microtomographic measurements of the sauropod left premaxilla were performed using the Imaging and Medical Beamline (IMBL) at the Australian Synchrotron. For this investigation, acquisition parameters included a pixel size of 100 × 100 µm, a pink beam of X-rays with a peak intensity of 220 keV generated using a superconducting multipole wiggler operating at 3T, a sample–to–detector distance of 500 mm, and use of the ‘Quartz' Hamamatsu C9252-DK14 flat panel photodiode array imager (2432 × 100 pixels). As the height and width of the specimen exceeded the detector field-of-view, the specimen was aligned axially relative to the beam, its centre of rotation shifted toward one edge of the detector. Nineteen successive scans were required to cover the full specimen volume, each consisting of 1800 equally spaced angle shadow-radiographs with an exposure length of 0.1 s, obtained every 0.10° as the sample was continuously rotated 180° about its vertical axis. Vertical translation of the specimen between tomographic scans was 80 mm. 100 dark (closed shutter) and beam profile (open shutter) images were obtained for calibration before and after shadow-radiograph acquisition. Total time for the scan was 70 min.

The raw 16-bit radiographic series were normalized relative to the beam calibration files and stitched with the in-house software ‘IMBL-Stitch' to yield a 32-bit dataset which was reconstructed to yield a three-dimensional tomogram by the filtered-back projection method using the CSIRO's X-TRACT [81]. All tomographic scan data were rendered and visualized in Dragonfly v.2020.1.1.809 (Object Research Systems: www.theobjects.com/dragonfly).

All elements were surface scanned at Australian Age of Dinosaurs Museum of Natural History using an Artec Space Spider (www.artec3d.com/portable-3d-scanners/artec-spider-v2). All three-dimensional surface models were manipulated, and the skull reconstruction assembled, in Artec Studio 15 Professional (www.artec3d.com/3d-software/artec-studio). The screenshots of the elements used in the figures of three-dimensional models were taken in MeshLab (www.meshlab.net), owing to its customizable lighting. All figures were assembled in Adobe Photoshop 2022 and 2023 (www.adobe.com/au/products/photoshop.html) and outlined and annotated in Adobe Illustrator 2022 and 2023 (www.adobe.com/au/products/illustrator.html).

We scored AODF 0906 for the phylogenetic matrix of Poropat *et al*. [41]. We modified one existing character and added four new ones, while also altering several character scores for operational taxonomic units (OTUs) already included in the matrix based on new information on *Diamantinasaurus matildae* [41,43] and *Phuwiangosaurus sirindhornae* [82], as well as re-evaluation of the CT scan data of *Sarmientosaurus musacchioi* [23]. These additions and changes are documented in the appendix. The revised data matrix comprises 126 OTUs, scored for 556 characters, and is provided as a TNT file (electronic supplementary material).

A taxonomic aside: we have referred to USNM 5730 as simply *Brachiosaurus* in [2], and as ‘*Brachiosaurus*' sp. in this paper and a previous one [41], because the referral of this specimen to *Brachiosaurus altithorax* by D'Emic and Carrano [24] was not made based on direct anatomical overlap between that specimen and the holotype of the taxon (FMNH P25107), which comprises only postcrania [83–85]. The other specimen assigned to *Brachiosaurus altithorax* by D'Emic and Carrano [24] (BYU 9754(4744)/USNM 21903) fails to bridge the anatomical gap: it comprises only postcrania, like the holotype. Thus, the referral of USNM 5730 to *Brachiosaurus* is circumstantial: the skull represents a brachiosaurid, it is from the Morrison Formation, and the only brachiosaurid heretofore named from the Morrison Formation is *Brachiosaurus altithorax*. In our view, this laissez faire taxonomic approach is not adequate grounds for referral, especially given that the Morrison Formation represents approximately 7 Myr of sedimentary deposition [86].

Phylogenetic analyses were run in TNT v.1.6 [87–89]. Eighteen of the 556 characters were treated as ordered (11, 14, 15, 27, 40, 51, 104, 122, 147, 148, 195, 205, 259, 297, 426, 435, 472 and 510). Eight taxa that have been determined in previous analyses to be unstable [6,41,90,91] were excluded *a priori*: *Astrophocaudia slaughteri*, *Australodocus bohetii*, *Brontomerus mcintoshi*, *Fukuititan nipponensis*, *Fusuisaurus zhaoi*, *Liubangosaurus hei*, *Malarguesaurus florenciae* and *Mongolosaurus haplodon*. The protocol employed for the equal weights and extended implied weights analyses was identical to that used by Poropat *et al*. [41]: under ‘New Technology Search', we used the ‘Stabilize Consensus' option with sectorial searches, drift and tree fusing. After five rounds of consensus stabilizing, the resultant trees were used as the starting topologies for a ‘Traditional Search', which used tree bisection–reconnection (TBR). Two versions of the analysis were run: one with equal character weighting, the other with extended implied weighting and a *k*-value of 9 [92,93]. Following Poropat *et al*. [41], two further taxa were excluded *a priori* from analyses applying equal character weighting, namely the ‘Cloverly titanosauriform' and *Ruyangosaurus giganteus*; these taxa were retained in the extended implied weighting analysis.

## 4. Systematic palaeontology

Sauropoda [94]

Macronaria [95]

Titanosauriformes [96,97]

Somphospondyli [95]

Diamantinasauria [41]

*Diamantinasaurus matildae* [9]

### 4.1. Holotype specimen

AODF 0603 (‘Matilda'; AODL 0085): dentary fragment, tooth, three partial cervical ribs, three incomplete dorsal vertebrae, dorsal ribs, fragmentary gastralia, five coalesced sacral vertebrae, isolated sacral processes, right and left scapulae, right coracoid, partial right sternal plate, right and left humeri, right and left ulnae, right radius, right and left metacarpals I–V, eight manual phalanges (including right manual ungual I-2), right and left ilia, right and left pubes, right and left ischia, right femur, right tibia, right fibula, right astragalus, associated fragments [37,43].

### 4.2. Previously referred specimens

AODF 0836 (‘Alex'; AODL 0127): left frontal, right and left parietals, left squamosal, right and left quadrates, braincase (comprising supraoccipital, right and left exoccipital–opisthotics, basioccipital, partial basisphenoid, right and left prootics, right and left laterosphenoids, right and left orbitosphenoids, and right and left possible sphenethmoids), left surangular, atlas intercentrum, axis, cervical vertebrae III–VI, middle/posterior cervical vertebral neural arch, three dorsal vertebrae, dorsal ribs, two co-ossified sacral vertebrae, right scapula, right and left iliac preacetabular processes, right and left pubes, right and left ischia, and abundant associated fragments, many representing ribs or partial vertebrae [38,41]; AODF 0663 (‘Oliver'; AODL 0122): one cervical rib, two dorsal vertebral centra, three dorsal vertebral neural arches, several dorsal ribs, left scapula, right humerus, right manual ungual I-2, right femur, and associated fragments [44].

### 4.3. Newly referred specimen

AODF 0906 (‘Ann'; AODL 0252): partial skull comprising left premaxilla, left maxilla, left lacrimal, left frontal, left parietal, right and left postorbitals, right and left squamosals, right and left quadratojugals, right and left quadrates, right and left pterygoids, left ectopterygoid, braincase (comprising supraoccipital, partial right and left exoccipital–opisthotics, fragmentary basioccipital, right and left prootics, right and left laterosphenoids, right and left orbitosphenoids, and a possible right sphenethmoid), right and left dentaries, left surangular, ?left ceratobranchial, four dorsal ribs, five sacral centra, several sacral processes, one anterior caudal vertebra, one chevron, left ilium, left pubis, right and left ischia, right and left femora, right and left tibiae, right and left fibulae, a probable right astragalus fragment, right metatarsals I–V, right pedal phalanges III-1–3 and IV-1–2, and associated fragments.

### 4.4. Newly identified diagnostic characteristics

Quadratojugal and quadrate with horizontal ridge present across both elements anterior to their articulation point (lateral surface of quadrate, medial surface of quadratojugal).

### 4.5. Locality

AODL 0252 (The ‘Ann' site), Elderslie Station, approximately 60 km NW of Winton, Queensland, Australia. Approximate coordinates 22° 15′ S, 142° 30′ E.

### 4.6. Horizon

Winton Formation; Cenomanian–lowermost Turonian, lower Upper Cretaceous [98,99].

## 5. Skull

### 5.1. General shape

**Figure 3.**
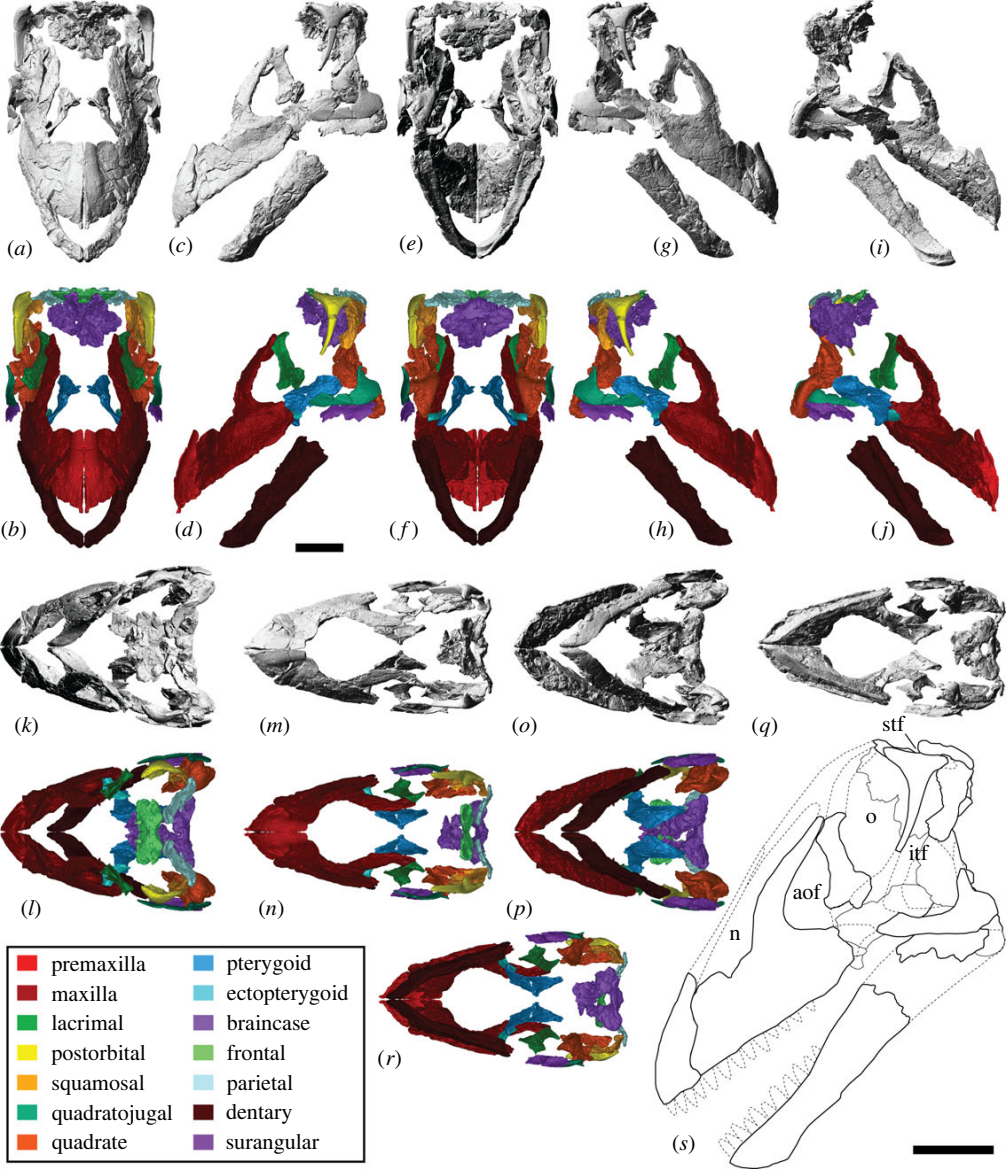
*Diamantinasaurus matildae* reconstructed skull (AODF 0906). (*a–r*) *Diamantinasaurus matildae* referred skull (AODF 0906) reconstruction in (*a,b*) anterior, (*c,d*) left lateral, (*e,f*) posterior, (*g,h*) right lateral, (*i,j*) left medial (right elements removed), (*k,l*) dorsal, (*m,n*) anterodorsal, (*o,p*) ventral and (*q,r*) posteroventral views. (*a–r*) are screenshots of three-dimensional models derived from surface scans. (*s*) *Diamantinasaurus matildae* referred skull (AODF 0906) reconstruction line drawing in left lateral view. aof, antorbital fenestra; itf, infratemporal fenestra; n, naris; o, orbit; stf, supratemporal fenestra. Scale bars = 100 mm.

The reconstructed skull of AODF 0906 is approximately 500 mm long anteroposteriorly. Based on the transverse width of the incomplete braincase, the mediolateral width of the left parietal (table 1), and the three-dimensional reconstruction of the skull presented herein (figure 3), the skull would have been approximately 250 mm wide transversely along its posterior margin. The incomplete left quadrate is approximately 210 mm tall dorsoventrally, indicating the minimum height of the posterior margin of the skull. Thus, the skull of AODF 0906 is slightly larger than the holotype skull of *Sarmientosaurus musacchioi* [23].

**Table 1.** Measurements of the skull and hyobranchial elements of AODF 0906 *Diamantinasaurus matildae*. An asterisk (*) indicates a tentative measurement based on an incomplete or distorted specimen.

element	dimension measured	measurement (mm)
premaxilla (left)	mesiodistal length along jawline	77
	dorsoventral height (perpendicular to jawline)	146
maxilla (left)	mesiodistal length along jawline	246*
	maximum dorsoventral height of base (anterior)	114
	maximum dorsoventral height of base (posterior)	74
	maximum dorsoventral height (total)	220
lacrimal (left)	maximum dorsoventral height	110
	maximum anteroposterior length (dorsal)	47
	maximum mediolateral width (dorsal)	24
	maximum anteroposterior length (ventral)	68
	maximum mediolateral width (ventral)	23
frontal (left)	maximum anteroposterior length	58*
	maximum mediolateral width	73*
parietal (left)	maximum dorsoventral height	51*
	maximum mediolateral width	108
postorbital (left)	maximum anteroposterior length	72*
	maximum dorsoventral height	117
postorbital (right)	maximum anteroposterior length	81
	maximum dorsoventral height	123
squamosal (left)	maximum anteroposterior length	50*
	maximum dorsoventral height	96*
squamosal (right)	maximum anteroposterior length	52*
	maximum dorsoventral height	96*
quadratojugal (left)	maximum anteroposterior length	149
	maximum dorsoventral height	80*
quadratojugal (right)	maximum anteroposterior length	135*
	maximum dorsoventral height	76*
quadrate (left)	maximum dorsoventral height	216*
pterygoid (right)	maximum anteroposterior length	139*
ectopterygoid (left)	maximum anteroposterior length	68
braincase	maximum anteroposterior length	140*
	maximum transverse width	150*
dentary (left)	maximum length along jawline	310*
	maximum dorsoventral height (anterior)	58*
	maximum dorsoventral height (posterior)	78
surangular (left)	maximum anteroposterior length	190
	maximum dorsoventral height	60
ceratobranchial (?left)	maximum anteroposterior length	231*

The premaxilla of *Diamantinasaurus* is not stepped, distinguishing the ‘snout' from that of taxa such as *Camarasaurus* and *Malawisaurus dixeyi* ([100,101]; although see [102], and [103]). However, it is likely that the snout was ‘boot'-shaped, as in brachiosaurids [104] and *Sarmientosaurus* [23]. As preserved, the premaxillary–maxillary index (PMI): [105] is approximately 45%. All other macronarians have PMI values greater than 60%, and those with anteriorly flattened snouts (like *Antarctosaurus wichmannianus*) have PMI values that exceed 75% [38,100,105]. However, the unusually low PMI value in *Diamantinasaurus* is almost certainly an underestimate, owing to the distortion to which the maxilla has been subjected. The nares appear to have been large and were presumably divided by the narial bar (comprising the narial processes of the premaxillae and the premaxillary processes of the nasals), although the direction in which they faced is unclear. By contrast, the antorbital fenestra on each side is small, ovate (dorsally tapered), faces laterally, and is bounded by the maxilla, lacrimal, and jugal; in all of these respects, it is akin to the antorbital fenestra of *Sarmientosaurus* [23]. The orbit of *Diamantinasaurus* was presumably ovate (tapering ventrally), although this cannot be stated with certainty, owing to the non-preservation of the jugal or prefrontal. The infratemporal fenestra is taller dorsoventrally than it is long anteroposteriorly, and is subtriangular, being expanded ventrally. This differentiates AODF 0906 from the titanosaur *Rapetosaurus*, which has slit-like infratemporal fenestrae [17]. The anterior margin of the infratemporal fenestra is situated posterior to the midpoint of the orbit, as in all macronarians [100,101,106,107]. The supratemporal fenestra is large, as in all neosauropods other than rebbachisaurids [104,108], and is bordered by the frontal, postorbital, squamosal and parietal. As in all somphospondylans, the greatest diameter of each supratemporal fenestra in *Diamantinasaurus* is less than the transverse distance between them [6,101,104]. The temporal bar precludes the supratemporal fenestra from being visible in lateral view, a feature that *Diamantinasaurus* shares with the titanosaurs *Nemegtosaurus*, *Rapetosaurus* and *Sarmientosaurus*, but that distinguishes it from non-titanosaurian macronarians and the titanosaur *Tapuiasaurus* [22,95,101]. Whether or not an external mandibular fenestra was present cannot be established with certainty in AODF 0906. However, it is probable that this fenestra was absent given that virtually all other macronarians lack external mandibular fenestrae [106,109]. *Tapuiasaurus* seems to be an exception: the presence of an external mandibular fenestra was tentatively inferred by Martínez *et al*. [23], although this opening was not described by either Zaher *et al*. [21] or Wilson *et al*. [22]. However, given the existence of near-identical elliptical openings on both mandibles (MZSP-Pv 807: PDM, pers. obs. 2019), we contend that *Tapuiasaurus* is characterized by the presence of an external mandibular fenestra.

### 5.2. Premaxilla

**Figure 4.**
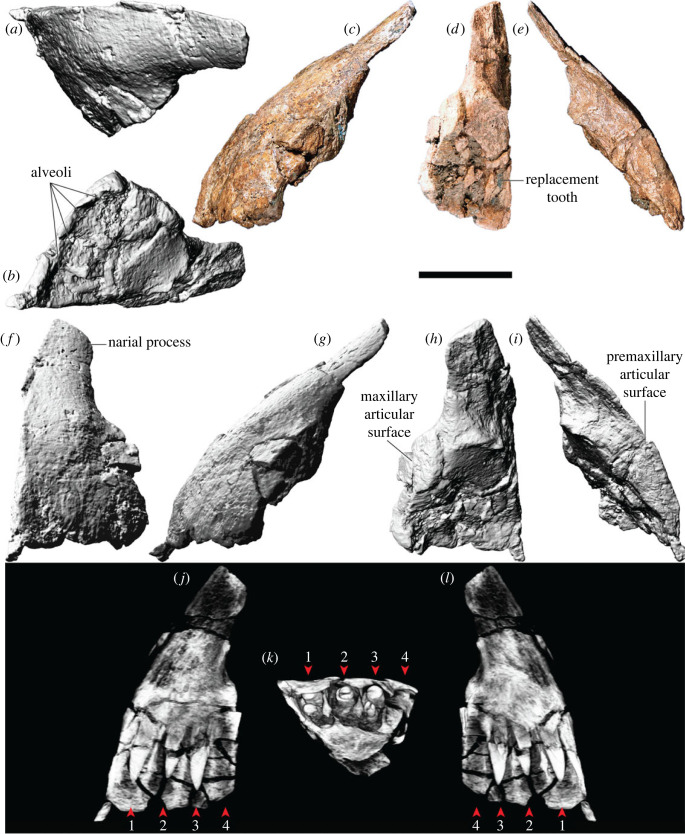
*Diamantinasaurus matildae* referred left premaxilla (AODF 0906). (*a–i*) Left premaxilla in (*a*) dorsal, (*b*) ventral, (*c*,*g*) lateral, (*d*,*h*) posterior, (*e*,*i*) medial and (*f*) anterior views. (*a*,*b*) and (*f–i*) are screenshots of three-dimensional models derived from surface scans, and (*c–e*) are photographs. (*j–l*) Three-dimensional digital models (derived from synchrotron data) of the left premaxilla in (*j*) labial, (*k*) occlusal and (*l*) lingual views. Numbers 1–4 indicate alveoli. Scale bar = 50 mm.

The body of the left premaxilla is complete and generally well preserved, despite having suffered extensive fracturing (figure 4). The lateral surface of the premaxilla of AODF 0906 is smoothly convex (figure 4*c,g*), lacking the vascular grooves seen in *Nemegtosaurus* [104,110]. A slightly rough texture characterizes the lanceolate articular surface for the right premaxilla, which would have faced medially (figure 4*i*). The premaxilla articulates with the maxilla posteriorly via a similarly lanceolate articular surface (figure 4*d,h*). The medial surface of the premaxilla is overlapped by the premaxillary process of the maxilla. In lateral view, the premaxilla–maxilla contact is essentially straight, as in non-titanosauriform sauropods, *Euhelopus zdanskyi* [111–114], and some titanosaurs (e.g. *Malawisaurus* [115], *Muyelensaurus pecheni* [116], *Tapuiasaurus* [21,22]); this distinguishes *Diamantinasaurus* from brachiosaurids and *Nemegtosaurus*, in which this contact is sinuous [20,26]. Lateral to the tooth row, there is a prominent plate of bone, as in sauropods generally [106,117].

The premaxilla of AODF 0906 appears to lack maxillary processes. If this absence is genuine, this would serve to distinguish *Diamantinasaurus* from *Europasaurus holgeri* and *Camarasaurus*, in which both dorsomedial and ventromedial maxillary processes are present on the premaxilla [118,119], and from *Malawisaurus* and *Tapuiasaurus*, in which a single medial process is present [22,115,120]. However, given that maxillary processes are positioned near the base of the narial process of the premaxilla, that they tend to be relatively fragile, and that this portion of the premaxilla is incomplete in AODF 0906, it is possible that they were present in *Diamantinasaurus*, but have been lost.

The premaxilla of AODF 0906 hosts four tooth positions (figure 4*b,j–l*), as in sauropods generally [8,109]. The only reported exception—an embryonic titanosaurian from the Late Cretaceous of Argentina, described by Kundrát *et al*. [121] as having five premaxillary alveoli—might have been incorrectly interpreted: based on their size relative to each other and to the other alveoli, the mesialmost two alveoli in this specimen could represent a single alveolus. Although the active teeth have been dislodged from the premaxilla in AODF 0906, a total of five replacement teeth are present in the crypt (figure 4*j–l*): two in the first alveolus (both of which are partly exposed on the specimen in lingual view owing to loss of part of the wall of the crypt), one in the second alveolus, and two in the third one. The fourth alveolus is vacant.

### 5.3. Maxilla

**Figure 5.**
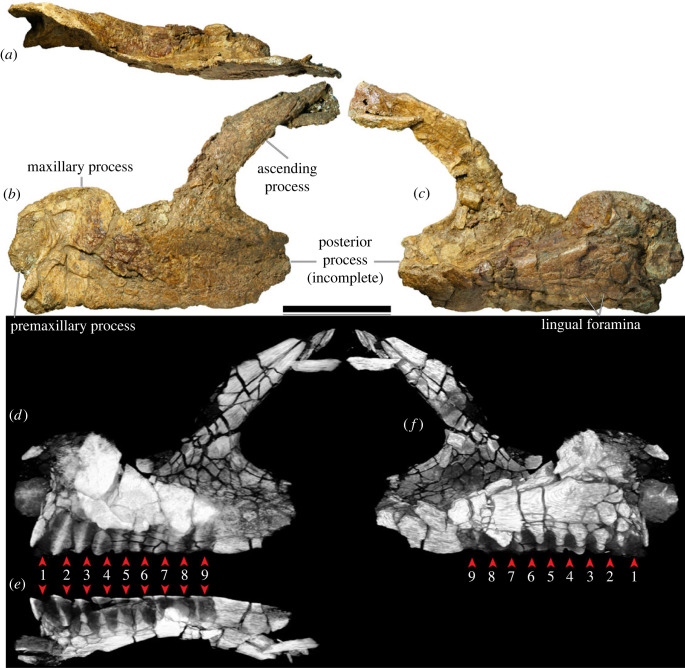
*Diamantinasaurus matildae* referred left maxilla (AODF 0906). (*a–c*) Photographs of the left maxilla in (*a*) dorsal, (*b*) lateral and (*c*) medial views. (*d–f*) three-dimensional digital models (derived from medical CT data) of the left maxilla in (*d*) lateral, (*e*) ventral and (*f*) medial views. Numbers 1–9 indicate alveoli. Scale bar = 50 mm.

**Figure 6.**
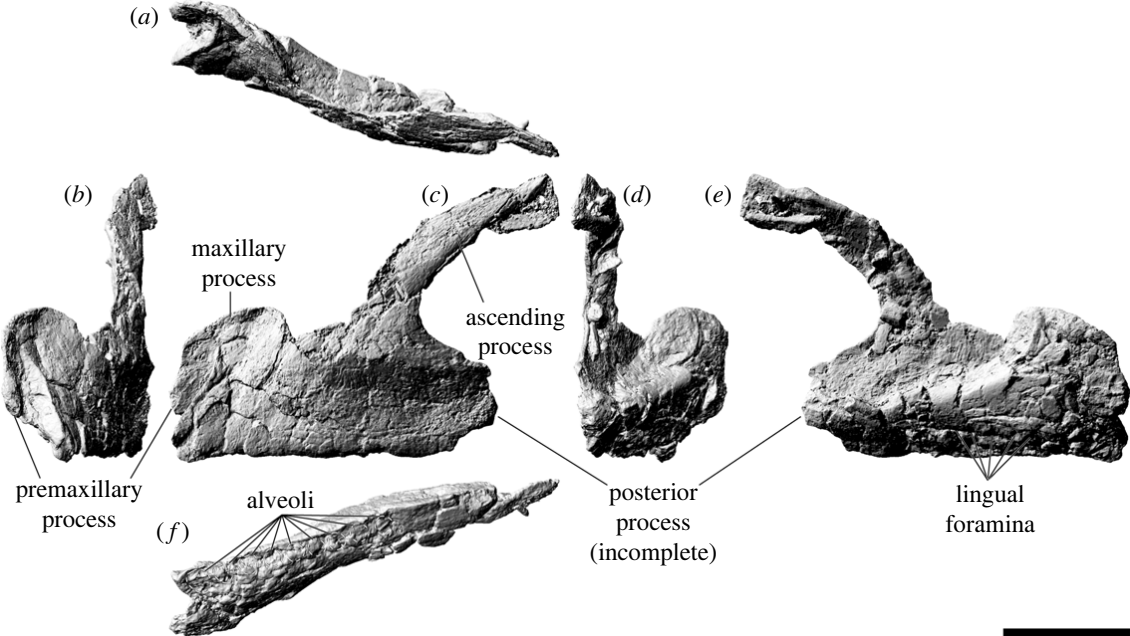
*Diamantinasaurus matildae* referred left maxilla (AODF 0906). (*a–f*) Three-dimensional digital models (derived from surface scans) of the left maxilla in (*a*) dorsal, (*b*) anterior, (*c*) lateral, (*d*) posterior, (*e*) medial and (*f*) ventral views. Scale bar = 50 mm.

The left maxilla (figures 5 and 6), which is lacking only the active dentition and the terminus of the posterior process, has suffered such extensive distortion that precise physical articulation with the premaxilla is not possible. Regardless, it is still morphologically informative.

The maxilla of AODF 0906 comprises a subrectangular dentigerous portion, a posterodorsally tapered ascending process, and an incomplete posterior process. Anteromedially, a prominent premaxillary process (anteromedial process *sensu* Wilson [20]) is present. Manifested as a semicircular flange, it is dorsoventrally taller, and consequently extends much further ventrally, than the same process in *Europasaurus* [119], *Camarasaurus* [118], ‘*Brachiosaurus*' [24], ‘*Astrodon*' / ‘*Pleurocoelus*' [122,123] and *Nemegtosaurus* [20]. Although this potentially represents an autapomorphy of *Diamantinasaurus*, the morphology of this process cannot be assessed in most somphospondylans, either owing to genuine absence or non-preservation. Moreover, it is likely that the present orientation and position of this process in AODF 0906 has been impacted by distortion: it is not hard to conceive that this process was originally oriented more anteriorly (and medially) than preserved. At the posteroventral base of this process, a small foramen is present; following Madsen *et al*. [118], this is the subnarial foramen.

The premaxillary process unites dorsally with a flange of bone that presumably represents the contact for the contralateral maxilla. We suggest that this flange, which is now essentially vertical, would have been inclined dorsomedially in life, owing to the mediolateral compression to which the maxilla has been subjected, and based on the morphology of the premaxilla. Posterior to this flange, the dorsomedial margin of the maxilla is incomplete; it is quite likely that this surface was more extensive *in vivo*. A similar inference has been made about an isolated titanosaur maxilla from the mid-Cretaceous of Argentina (UNPSJB-PV 583 [23,124]).

The lateral surface of the dentigerous portion appears to have been completely smooth (figure 5*b*), lacking the vascular grooves seen in nemegtosaurids and, to a lesser extent, in *Giraffatitan* and *Sarmientosaurus* [6,20,23,25,104]. However, the poor preservation of this surface in AODF 0906 (figure 6*c*) might have destroyed any evidence of these, had they been present. If a preantorbital opening was present (which is unclear), it was only expressed as a shallow fossa, as in most macronarians [6,21,38,125]. Similarly, it is not clear whether or not an additional foramen anterior to the preantorbital fenestra, which is present in the titanosaurs *Nemegtosaurus* and *Tapuiasaurus* [20–22], was present in AODF 0906.

The preservation of the medial surface of the dentigerous portion of the maxilla evinces the degree of distortion to which this element was subjected (figures 5*c,f* and 6*e*). The dorsal surface of the crypt, which should be completely smooth, is broken into segments that have shifted slightly, despite remaining effectively in life position. Nevertheless, morphological information can be gleaned from the medial surface. As in all other sauropods, a rounded special foramen (*sensu* Edmund [126]; replacement foramen *sensu* Jin *et al*. [127]) is present dorsal to each tooth position. Although many of these special foramina are observable, none are complete—the ventral halves of all are missing. Dorsal to these special foramina, the medial surface of the crypt is smooth and essentially flat. Although the dentigerous portion of the maxilla articulated with the palatine and ectopterygoid posteromedially, neither of these contacts is evident, owing to breakage. The roof of the crypt is manifested as a posteroventrally slanted shelf, walled laterally by the base of the ascending process.

The posterior process of the maxilla of AODF 0906 is incompletely preserved but was clearly not as elongate as that of *Nemegtosaurus* [20], *Rapetosaurus* [17], or the embryonic titanosaurs from Auca Mahuevo [128–130]. Based on the preserved length of the dentary and the morphology of the quadratojugals in AODF 0906, we suggest that the posterior margin of the posterior process contacted the jugal only, as in most macronarians [6,22,23,26,38,106,119]; by contrast, in *Camarasaurus* and *Nemegtosaurus*, the posterior process also has a substantial contact with the quadratojugal [20,118]. Whether or not the ventral margin of the posterior process of the maxilla possessed a distinct ventral emargination, as in other titanosauriforms [3,6,38], is not known.

The ascending process of the maxilla contacts the nasal and lacrimal dorsally. It forms the ventral and lateral margins of the naris and the anteroventral margin of the antorbital fenestra. In AODF 0906, the ascending process is almost complete, albeit broken. The grain of the bone, and the shapes of the fragments near the base, demonstrate that the ascending process should project more dorsally than preserved, relative to the dentigerous portion. However, the distortion to which both the dorsal half of the dentigerous portion and the ventral portion of the ascending process have been subjected implies that a simple dorsal orientation of the ascending process is not correct either: the present vertical orientation of the ascending process is probably only slightly exaggerated relative to its *in vivo* state. It seems unlikely that the ascending process projected posteriorly beyond the posterior process; as such, the ascending process in *Diamantinasaurus* appears to be characterized by the same morphology as most titanosauriforms, other than the titanosaurs *Nemegtosaurus* and *Rapetosaurus* [6,131].

As preserved, the tooth row of AODF 0906 terminates just anterior to the antorbital fenestra (figure 5*d–f*, 6*f*). However, given that the angle of the ascending process—and, consequently, the position of the anterior margin of the antorbital fenestra—has been altered by taphonomic distortion, we contend that it is more likely that the tooth row terminated somewhere below the antorbital fenestra. This would suggest that this aspect of the tooth row of *Diamantinasaurus* is more similar to titanosaurs such as *Rapetosaurus* and *Sarmientosaurus*, but differentiates it from others wherein the tooth row terminates anterior to the external naris, including *Nemegtosaurus* and *Tapuiasaurus*, as well as brachiosaurids [26,100,101,104,132]. Based on CT scan data of the left maxilla of AODF 0906, nine alveoli are present (figure 5*d–f*). This is fewer than in the early-diverging macronarian *Europasaurus* (12–13: [119]), brachiosaurids (10–14: [24–26,133]), *Euhelopus* (10: [114]), and the titanosaurs *Sarmientosaurus* (11–12: [23]) and *Tapuiasaurus* (12: [21,22]). By contrast, it is greater than the number seen in the Auca Mahuevo titanosaur embryos (7–8: [129]) and (probably) *Narambuenatitan* (at least 8: [134]). *Camarasaurus* ranges from having 8 to 10 maxillary alveoli [118,135–140], and in nemegtosaurid titanosaurs either eight or nine maxillary alveoli are present [18–20]. Given that maxillary alveolus count in macronarian sauropods can vary between the two sides of a single individual (e.g. *Sarmientosaurus* [23]), within taxa known from multiple exemplars (e.g. *Giraffatitan* [25]), and probably changed ontogenetically (based on the low maxillary tooth count in the Auca Mahuevo titanosaur embryos [129]), attaching significance to maxillary alveolus count is tenuous. Thus, the relatively low maxillary alveolus count of *Diamantinasaurus* simply serves to distinguish it from most titanosauriforms other than nemegtosaurids.

### 5.4. Lacrimal

**Figure 7.**
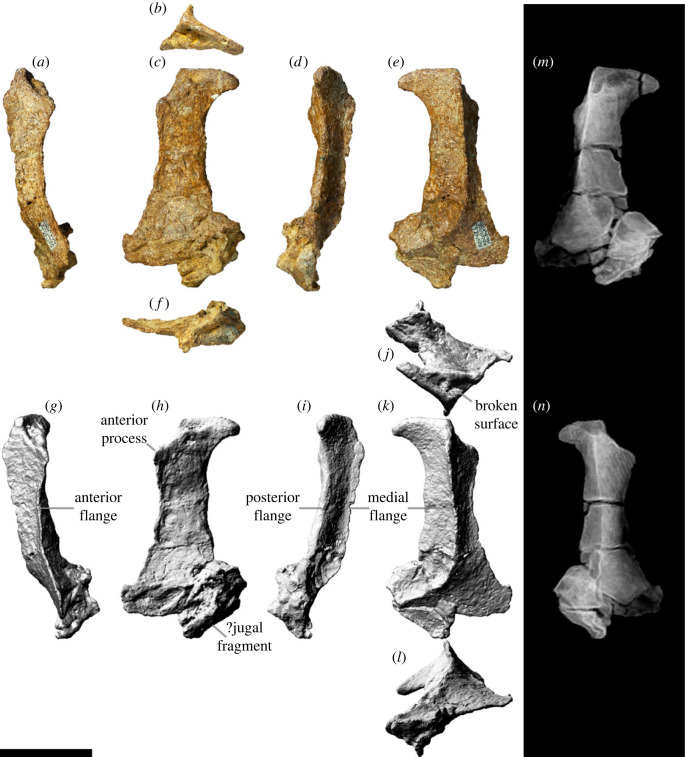
*Diamantinasaurus matildae* referred left lacrimal (AODF 0906). (*a–f*) Photographs of the left lacrimal in (*a*) anterior, (*b*) dorsal, (*c*) lateral, (*d*) posterior, (*e*) medial and (*f*) ventral views. (*g–l*) Three-dimensional digital models (derived from surface scans) of the left lacrimal in (*g*) anterior, (*h*) lateral, (*i*) posterior, (*j*) dorsal, (*k*) medial and (*l*) ventral views. (*m,n*) Three-dimensional digital models (derived from medical CT data) of the left lacrimal in (*m*) lateral and (*n*) medial views. Scale bar = 50 mm.

The lacrimal (figure 7) forms the posterior margin of the antorbital fenestra and the anterior margin of the orbit. The dorsal margin would have contacted the ascending process of the maxilla, the nasal and the prefrontal. If the lacrimal of AODF 0906 is complete dorsally, then the facet for the ascending process of the maxilla is a deep furrow (figure 7*b,j*); however, it is far more likely that the apex of this element is incomplete, based on both its poor preservation and the height of the lacrimal relative to that of the ascending process of the maxilla (figures 5 and 6). Thus, it cannot be determined whether or not the lacrimal of *Diamantinasaurus* clasped the ascending process of the maxilla, as in *Sarmientosaurus* [23]. The dorsal four-fifths of the lacrimal (as preserved) is triangular in cross-section, with the element divided into lateral, anteromedial and posteromedial surfaces; a similar morphology was reported in *Rapetosaurus* [17] and *Sarmientosaurus* [23]. By contrast, at its ventral end, the lacrimal is anteroposteriorly flared and mediolaterally compressed. The lateral surface is flat to shallowly concave along its length and separated from the other two surfaces by pronounced flanges. The anteromedial and posteromedial surfaces are also separated by a pronounced medial flange (figure 7*e,k*). The anteromedial surface is shallowly concave, whereas the posteromedial surface is mostly flat, being concave only near the medial flange. The lacrimal foramen cannot be observed on the posteromedial surface, nor in CT scans of the specimen, owing to the distortion to which the lacrimal has been subjected (figure 7*m,n*). The lacrimal of AODF 0906 possesses a very weak anterior process (figure 7*c,h*), as in *Giraffatitan*, *Nemegtosaurus* and *Sarmientosaurus* [20,23,25]; this distinguishes *Diamantinasaurus* from *Abydosaurus*, *Bonitasaura salgadoi*, *Rapetosaurus* and *Tapuiasaurus*, wherein the anterior process is more pronounced [17,22,26,141]. The dorsal end of the lacrimal also possesses a posterior process similar to that seen in *Nemegtosaurus* [20]; a similar process appears to be present in *Sarmientosaurus*, although in this taxon it is obscured laterally by the prefrontal [23].

The ventral margin of the lacrimal would have contacted the jugal ventrally. Indeed, the AODF 0906 lacrimal appears to be preserved in articulation with a fragment of the jugal (figure 7*c,h*). Based on the morphology of the ventral end of the lacrimal, and our reconstruction of the skull, we infer that the jugal made a small contribution to the antorbital fenestra, as in *Giraffatitan*, *Nemegtosaurus*, *Rapetosaurus* and *Sarmientosaurus* [6,104,142]; this distinguishes AODF 0906 from *Tapuiasaurus*, wherein the contribution of the jugal to the antorbital fenestra is extensive [21,22].

### 5.5. Frontal

**Figure 8.**
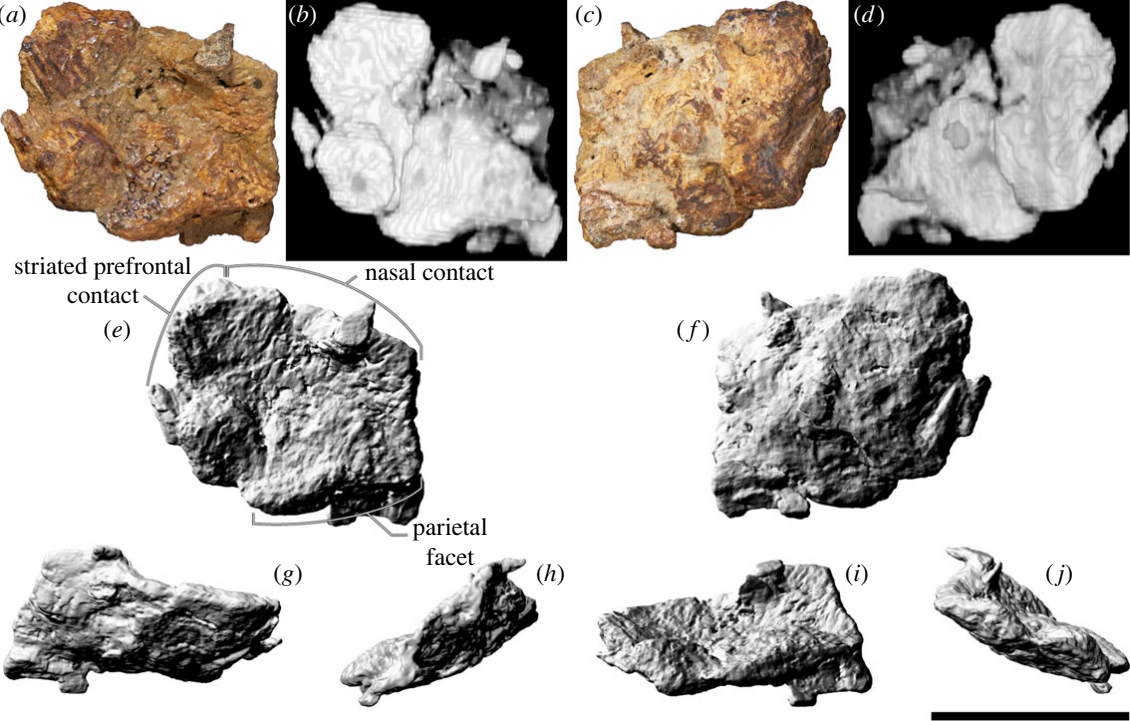
*Diamantinasaurus matildae* referred left frontal (AODF 0906). (*a–d*) Left frontal in (*a*,*b*) dorsal and (*c*,*d*) ventral views. (*a*) and (*c*) are photographs, (*b*) and (*d*) are three-dimensional models derived from CT data. (*e–j*) Three-dimensional models (derived from surface scans) of the left frontal in (*e*) dorsal, (*f*) ventral, (*g*) anterior, (*h*) medial, (*i*) posterior and (*j*) lateral views. Scale bar = 50 mm.

Only the left frontal is preserved (figure 8), and it appears to be incomplete laterally and to have suffered significant *post mortem* damage. Despite its incompleteness, it is approximately 25% wider mediolaterally than long anteroposteriorly. Thus, as in all non-euhelopodid titanosauriforms [6,38,131,143], including a previously referred specimen (AODF 0836) of *Diamantinasaurus* [38,41], the frontal of AODF 0906 is shorter anteroposteriorly than it is wide mediolaterally. The dorsal surface is broadly concave mediolaterally (figure 8*a,e*), although it becomes convex medially, as in the early-branching somphospondylan *Phuwiangosaurus sirindhornae* and the titanosaurs *Antarctosaurus*, AODF 0836, *Bonatitan*, *Bonitasaura*, *Narambuenatitan*, *Rapetosaurus* and *Saltasaurus loricatus* [3,6,17,38,41,134,141,144–147]. By contrast, the ventral surface of the AODF 0906 frontal is broadly convex but irregularly undulatory (figure 8*c,f*), which is presumably a consequence of deformation and/or under-preparation. The ventral surface would have been sutured to the dorsal margin of the orbitosphenoid and the anterodorsal portion of the laterosphenoid; unfortunately, neither point of contact is well preserved.

The anterior margin of the frontal is gently convex. The medial portion of this margin abutted the posterior margin of the nasal, whereas the lateral portion was overlapped by the prefrontal. The frontal–nasal contact is practically complete and, as in all macronarians, straight in dorsal view [6,131]. By contrast, the prefrontal articular surface is incomplete both anteriorly and laterally. Towards the lateral margin, the dorsal and ventral surfaces of the frontal host anteromedially–posterolaterally projecting striations, with those on the dorsal surface situated further posteriorly than the same on the ventral surface. The posterior margin of the frontal is convex posteriorly in dorsal view and sinusoidal in posterior view. The medial half of the posterior margin hosts a flattened facet for the parietal; thus, the parietal suture was situated near the anterior margin of the supratemporal fenestra, as in all macronarians [6,143]. The lateral half of the posterior margin either contacted the anterolateral wing of the parietal (if such a structure was present and in contact with the postorbital), or contributed to the supratemporal fenestra, as was inferred for AODF 0836 [38,41]. The medial margin of the frontal is straight and anteroposteriorly convex. No signs of interdigitation or breakage are evident on the medial margin (figure 8*h*); thus, it seems safe to presume that the frontals were not fused, as in most sauropods [106,148], including AODF 0836 [38,41].

### 5.6. Parietal

**Figure 9.**
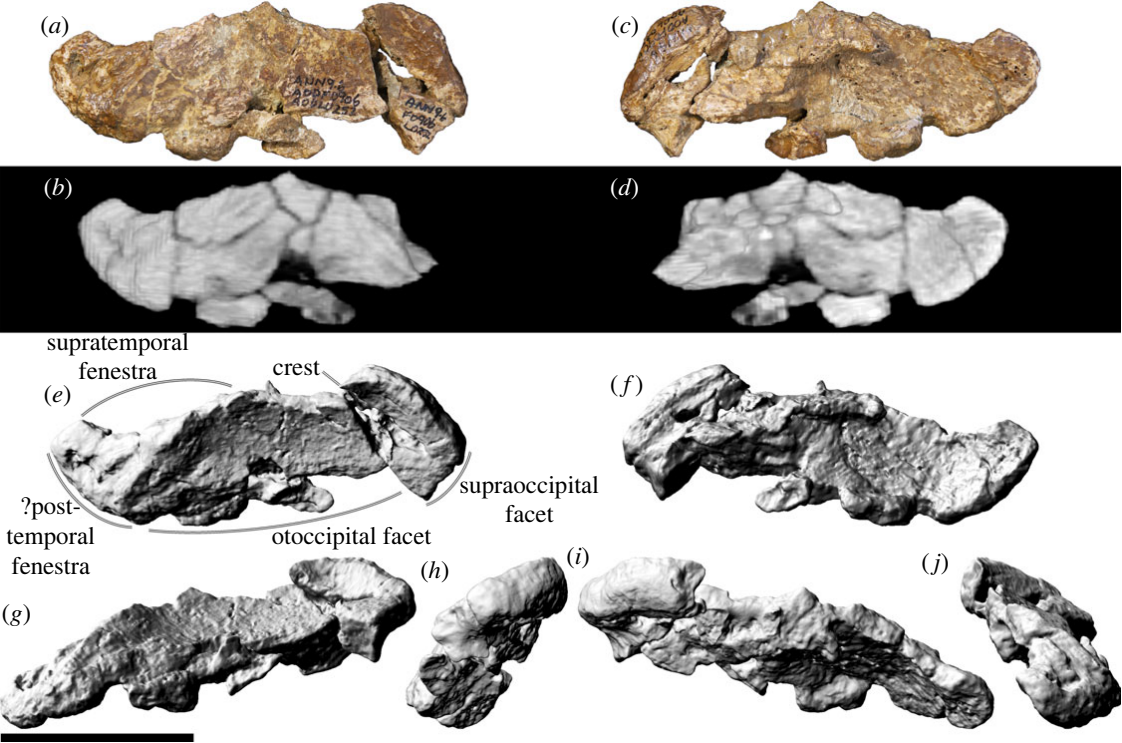
*Diamantinasaurus matildae* referred left parietal (AODF 0906). (*a–d*) Left parietal in (*a*,*b*) posterior and (*c*,*d*) anterior views. (*a*) and (*c*) are photographs, (*b*) and (*d*) are three-dimensional models derived from CT data. (*e–j*) Three-dimensional models (derived from surface scans) of the left parietal in (*e*) posterior, (*f*) anterior, (*g*) ventral, (*h*) medial, (*i*) dorsal and (*j*) lateral views. Scale bar = 50 mm.

Only the left parietal is present in AODF 0906 (figure 9), and, despite being neither complete nor well-preserved, it is still anatomically informative. Moreover, it is practically identical to the parietals of AODF 0836 [41]. The parietal is broader mediolaterally than tall dorsoventrally, and is slightly tapered laterally. The dorsoventral height of the parietal occipital process is greater than the maximum diameter of the foramen magnum, similar to *Giraffatitan* and *Sarmientosaurus*, but differentiating it from *Abydosaurus*, *Europasaurus* and titanosaurs such as *Antarctosaurus* and *Tapuiasaurus* [90,101,104]. As in other titanosaurs, the anterior margin is elevated such that it forms a crest [3,17,38]. The medial two-thirds of the posterior surface is shallowly concave (figure 9*a,e*), with a subtle ridge demarcating the medial and lateral margins (and presumably, when complete, the anterior margin). The lateral portion, by contrast, is shallowly convex dorsoventrally. Thus, AODF 0906 is similar to the parietals of AODF 0836, which were autapomorphically characterized as having their medial half concave and their lateral half convex [41]. The medial three-quarters of the dorsal margin of the parietal are dorsally convex in posterior view, whereas the lateral one-quarter (which would have formed the posterior margin of the supratemporal fenestra) is concave in posterior view. Unfortunately, the contact with the frontal has suffered extensive damage; thus, it is not clear if this contact occupied a deep trough, as in several other titanosaurs (e.g. *Isisaurus colberti*, *Nemegtosaurus*, *Rapetosaurus*, *Saltasaurus* and *Tapuiasaurus* [3,6,38]). The medial surface of the parietal, which appears to present finished bone, is still partially obscured by matrix. Thus, the nature of the inter-parietal contact is unclear. Whether or not a post-temporal fenestra was present in *Diamantinasaurus* is uncertain, although if there was one, the parietal would have contributed to its margin.

### 5.7. Postorbital

**Figure 10.**
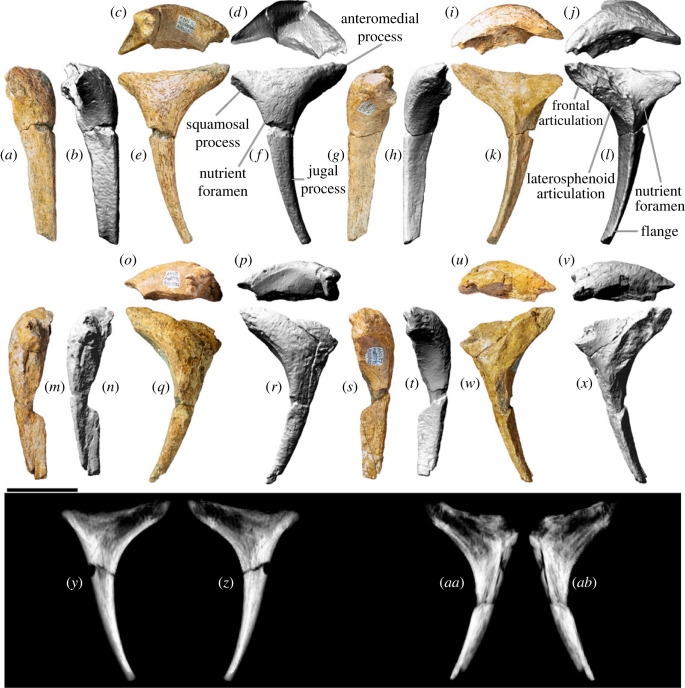
*Diamantinasaurus matildae* referred postorbitals (AODF 0906). (*a–l*) Right postorbital in (*a,b*) posterior, (*c,d*) ventral, (*e,f*) lateral, (*g,h*) anterior, (*i,j*) dorsal and (*k,l*) medial views. (*a*), (*c*), (*e*), (*g*), (*i*) and (*k*) are photographs, (*b*), (*d*), (*f*), (*h*), (*j*) and (*l*) are three-dimensional models derived from surface scans. (*m–x*) Left postorbital in (*m,n*) posterior, (*o,p*) ventral, (*q,r*) lateral, (*s,t*) anterior, (*u,v*) dorsal and (*w,x*) medial views. (*m*), (*o*), (*q*), (*s*), (*u*) and (*w*) are photographs, (*n*), (*p*), (*r*), (*t*), (*v*) and (*x*) are three-dimensional models derived from surface scans. (*y,z*) Three-dimensional digital models (derived from medical CT data) of the right postorbital in (*y*) lateral and (*z*) medial views. (*aa*–*ab*) Three-dimensional digital models (derived from medical CT data) of the left postorbital in (*aa*) lateral and (*ab*) medial views. Scale bar = 50 mm.

The right postorbital (figure 10*a–l,y,z*) is more complete than the left element (figure 10*m–x*, *aa*–*ab*); thus, the description below is based mainly on the former, with supplementary observations derived from the latter. In lateral view (figure 10*e,f*) the postorbital is triradiate, with a short anteromedial process, an even shorter posterior (squamosal) process, and an elongate anteroventral (jugal) process. The postorbital forms the posterodorsal margin of the orbit, the anterodorsal margin of the infratemporal fenestra, and the anterolateral margin of the supratemporal fenestra. The lateral surface of the postorbital bears a prominent nutrient foramen near the junction of the squamosal and jugal processes; the postorbitals of *Abydosaurus* and *Sarmientosaurus* possess a nutrient foramen in the same position [23,26]. Otherwise, the lateral surface of the postorbital of AODF 0906 is smoothly convex in all directions, albeit only shallowly so on the jugal process. By contrast, the anterior surface is mediolaterally convex and dorsoventrally concave (figure 10*g,h*), whereas the posterior surface (which would have articulated with the postorbital process of the jugal) is mediolaterally and (shallowly) dorsoventrally concave (figure 10*a,b*). The jugal process as a whole is anteroposteriorly compressed, as in macronarians generally [8,95]. In medial view (figure 10*k,l*), the anterior and posterior surfaces of the jugal process are divided by a flange that dissipates dorsally at the laterosphenoid articulation. This anteroposteriorly elongate, shallowly concave facet is more extensive anteroposteriorly in *Diamantinasaurus* than in *Giraffatitan* [25], although it is similar in extent to that of *Europasaurus* [119], *Euhelopus* [114] and *Sarmientosaurus* [23]. Anterior to the laterosphenoid facet lies the frontal facet, which is more strongly concave and faces anteromedially, rather than just medially. Dorsal to these facets, the dorsomedial surface (which would have formed the lateral margin of the supratemporal fenestra) is shallowly concave. The path of the supratemporal canal runs posterior to the laterosphenoid facet. On the medial surface, immediately posterior to the laterosphenoid facet, a small nutrient foramen is present. The squamosal process is broken in both postorbitals, but was clearly triangular and fairly short, albeit not reduced to the same extent as seen in *Antarctosaurus*, *Nemegtosaurus*, *Quaesitosaurus* and *Tapuiasaurus* [20–22,104].

### 5.8. Squamosal

**Figure 11.**
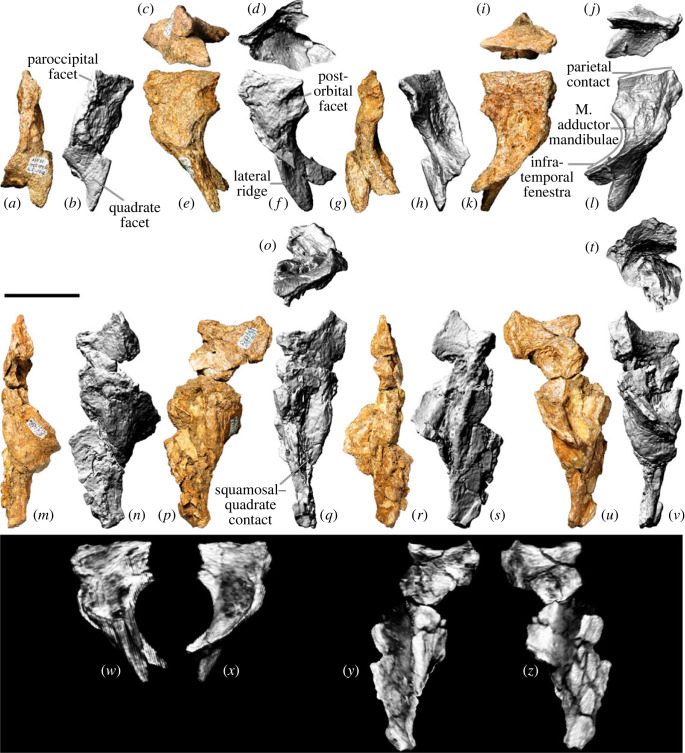
*Diamantinasaurus matildae* referred squamosals (AODF 0906). (*a–l*) Right squamosal in (*a,b*) posterior, (*c,d*) ventral, (*e,f*) lateral, (*g*,*h*) anterior, (*i,j*) dorsal and (*k,l*) medial views. (*a*), (*c*), (*e*), (*g*), (*i*) and (*k*) are photographs, (*b*), (*d*), (*f*), (*h*), (*j*) and (*l*) are three-dimensional models derived from surface scans. (*m–v*) Left squamosal (with attached left quadrate fragment) in (*m*,*n*) posterior, (*o*) ventral, (*p*,*q*) lateral, (*r*,*s*) anterior, (*t*) dorsal and (*u*,*v*) medial views. (*m*), (*p*), (*r*) and (*u*) are photographs, (*n–o*), (*q*), (*s–t*) and (*v*) are three-dimensional models derived from surface scans. (*w,x*) Three-dimensional digital models (derived from medical CT data) of the right squamosal in (*w*) lateral and (*x*) medial views. (*y,z*) Three-dimensional digital models (derived from medical CT data) of the left squamosal in (*y*) lateral and (*z*) medial views. Scale bar = 50 mm.

The right squamosal (figure 11*a–l*,*w–x*) is more complete and better-preserved than the left one (figure 11*m–v*,*y–z*). The latter is incompletely exposed, remaining in contact, albeit not in articulation, with a fragment of the dorsal section of the pterygoid process of the left quadrate. Consequently, the description below is based almost entirely on the right squamosal.

The squamosal was restricted to the postorbital region, distinguishing it from *Nemegtosaurus*, *Rapetosaurus* and *Tapuiasaurus*, wherein the squamosal extends beyond the posterior margin of the orbit [6]. Broadly speaking, the squamosal is comma-shaped in lateral view (figure 11*e,f*). The dorsal margin is manifested as a mediolaterally thin, shallowly anteroposteriorly concave ridge that terminates posteriorly at a small but pronounced point (figure 11*i,j*). The lateral surface of the short anterior process bears a posteriorly tapered facet to receive the posterior process of the postorbital. Posterior to the postorbital facet, the lateral surface is smoothly convex. Ventral to this facet, a prominent lateral ridge is present, which divides the anteroventral process into anterior and posterior surfaces. The anterior surface of the anteroventral process (figure 11*g,h*)), which is shallowly concave, forms the posterodorsal margin of the infratemporal fenestra. The mostly flattened posterior surface of the anteroventral process hosts the articular facet for the quadrate (figure 11*a,b*), which is not well preserved in either squamosal of AODF 0906; nevertheless, it appears to be bilobate, as is also the case in AODF 0836 [38,41] and *Sarmientosaurus*, as observed in CT data [23]. Although the ventral processes of both squamosals are incomplete in AODF 0906, it is presumed that the ventral tip of the anteroventral process of the squamosal contacted the dorsal process of the quadratojugal, as in most macronarians [95,100,106,132].

The dorsomedial surface of the squamosal would have articulated with the posterolateral margin of the parietal. It seems likely that the squamosal of *Diamantinasaurus* contributed to the supratemporal fenestra, contrasting with the titanosaurs *Nemegtosaurus*, *Quaesitosaurus* and *Tapuiasaurus* [20,22,100,106]. A subtle horizontal ridge separates the relatively small parietal facet from the rest of the medial surface of the squamosal, which is strongly concave and would have accommodated the *M. adductor mandibulae*. The posterior margin of the squamosal is expressed as a thin, anteroventrally–posterodorsally oriented ridge for most of its length, although the dorsal one-third broadens into a flat (albeit still mediolaterally narrow) facet against which the paroccipital process abutted. The ventral prong situated ventrolateral to the paroccipital articulation in the squamosals of the non-titanosaurian somphospondylan *Phuwiangosaurus* and the titanosaurs *Nemegtosaurus* and *Tapuiasaurus* is not evident in AODF 0906 and seems to be genuinely absent [17,20,22,149].

### 5.9. Quadratojugal

**Figure 12.**
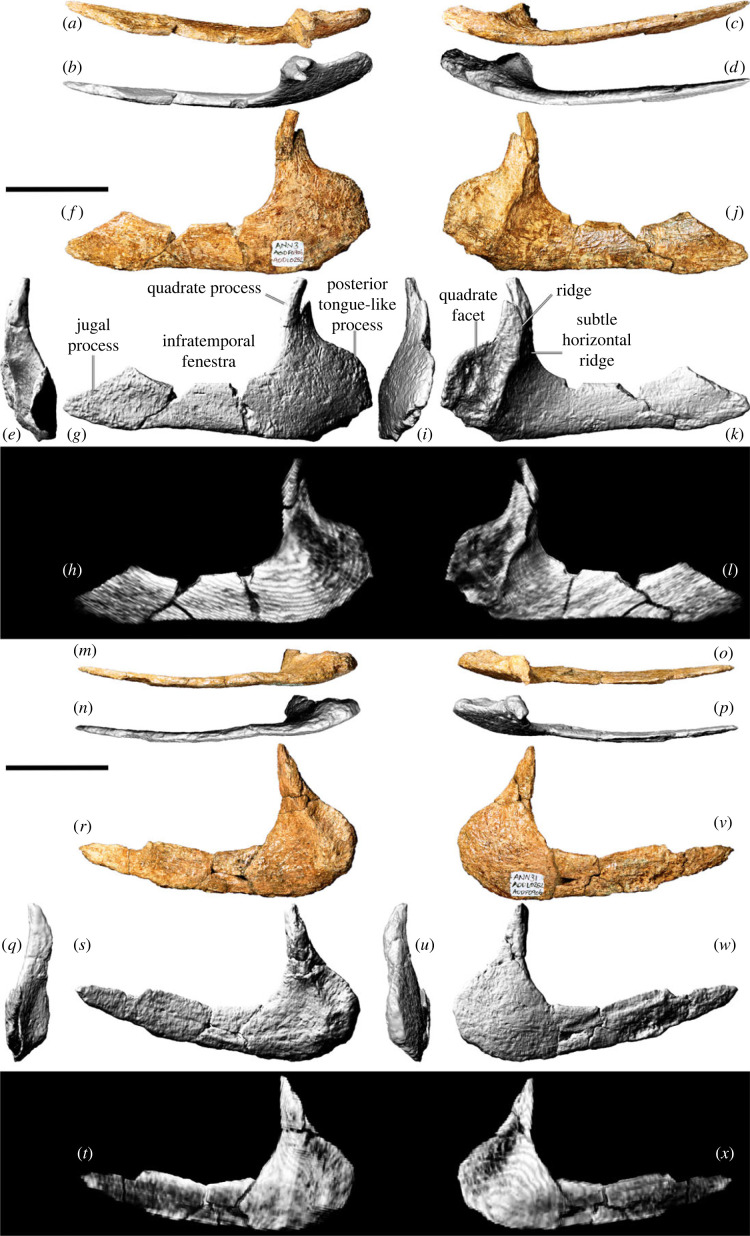
*Diamantinasaurus matildae* referred quadratojugals (AODF 0906). (*a–l*) Left quadratojugal in (*a*,*b*) dorsal, (*c*,*d*) ventral, (*e*) anterior, (*f–h*) lateral, (*i*) posterior and (*j–l*) medial views. (*a*), (*c*), (*f*) and (*j*) are photographs, (*b*), (*d–e*), (*g*), (*i*) and (*k*) are three-dimensional models derived from surface scans, and (*h*) and (*l*) are three-dimensional models derived from CT data. (*m–x*) Right quadratojugal in (*m*,*n*) ventral, (*o*,*p*) dorsal, (*q*) anterior, (*r–t*) medial, (*u*) anterior, and (*v–x*) lateral views. (*m*), (*o*), (*r*) and (*v*) are photographs, (*n*), (*p–q*), (*s*), (*u*) and (*w*) are three-dimensional models derived from surface scans, and (*t*) and (*x*) are three-dimensional models derived from CT data. Scale bar = 50 mm.

The left quadratojugal of AODF 0906 (figure 12*a–l*) is more complete than the right (figure 12*m–x*), and it forms the basis for the entire description of this element herein. The quadratojugal is approximately L-shaped in lateral view (figure 12*f–h*), with an elongate jugal (anterior) process and an apparently shorter quadrate (dorsal) process. The latter process is not complete in either quadratojugal, but it was likely sufficiently elongate to contact the anteroventral process of the squamosal. In both quadratojugals, the jugal process also appears to be incomplete; nevertheless, it can safely be presumed that the jugal process was more than 1.3 times the length of the quadrate process, as in all macronarians [95,100]. It seems unlikely that the anterior extremity of the jugal process of the quadratojugal possessed a ventral triangular projection, differentiating *Diamantinasaurus* from brachiosaurids, *Europasaurus*, *Nemegtosaurus*, *Quaesitosaurus* and *Tapuiasaurus* [5,38]. The angle between the quadrate and jugal processes is less than 90° in AODF 0906. In the titanosaur *Sarmientosaurus*, and in non-titanosaurian macronarians generally, these processes meet at an angle of 90° or lower [6,23,106,132]; by contrast, the angle between these processes is greater than 90° in *Nemegtosaurus* and *Tapuiasaurus*, as well as the Auca Mahuevo titanosaur embryos [20,22,128].

The lateral surface of the jugal process of the quadratojugal is entirely convex, whereas that of the base of the squamosal process, which is distinctly twisted and thickened mediolaterally, is shallowly concave. The medial surface of the jugal process is anteroposteriorly convex but dorsoventrally concave, and it terminates posteriorly in a ridge that demarcates the anterior margin of the quadrate facet (figure 12*j–l*). At approximately two-thirds of the preserved height of the quadrate facet, a subtle horizontal ridge is present, which is aligned with a similar ridge on the quadrate when the elements are articulated. The quadrate facet of the quadratojugal is strongly concave, with two small pits present near the posterior margin. The quadratojugal lacks the posteroventral hook seen in *Tapuiasaurus* [22]. However, a posterior tongue-like process, situated posterior to the quadrate facet, is present. A similar process is present in *Sarmientosaurus* and was regarded as an autapomorphy of that taxon by Martínez *et al*. [23]. To test its distribution, we include its presence as a new character (C555).

### 5.10. Quadrate

**Figure 13.**
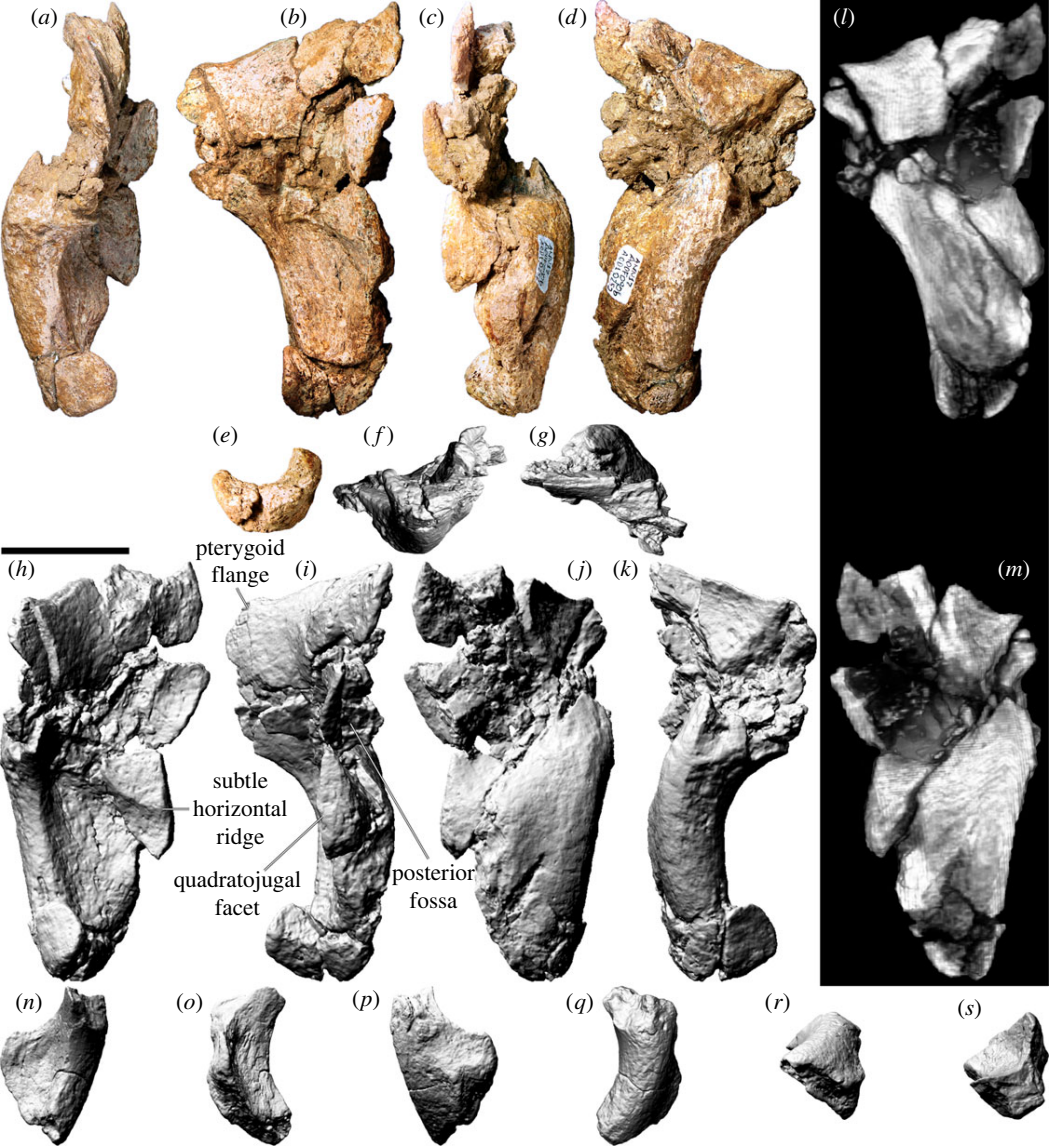
*Diamantinasaurus matildae* referred quadrates (AODF 0906). (*a–e*) Photographs of the left quadrate in (*a*) anterior, (*b*) lateral, (*c*) posterior, (*d*) medial and (*e*) ventral views. (*f–k*) Three-dimensional models (derived from surface scans) of the left quadrate in (*f*) ventral, (*g*) dorsal, (*h*) anterior, (*i*) lateral, (*j*) posterior and (*k*) medial views. (*l,m*) Three-dimensional models derived from CT scans of the left quadrate in (*l*) lateral and (*m*) medial views. (*n–s*) Three-dimensional models (derived from surface scans) of the right quadrate in (*n*) anterior, (*o*) lateral, (*p*) posterior, (*q*) medial, (*r*) dorsal and (*s*) ventral views. Scale bar = 50 mm.

The left quadrate (figure 13*a–m*) is almost complete but quite damaged, whereas the right quadrate (figure 13*n–s*) is only represented by a small, albeit far less distorted, ventral portion of the body. Consequently, the description below is based entirely on the left quadrate, which is virtually indistinguishable from the quadrates of AODF 0836 [41].

When complete, the quadrate would have been triangular in lateral and medial views, with a prominent anterior pterygoid flange. The quadrate was evidently oriented more or less vertically, as in some titanosaurs, such as *Sarmientosaurus*, but unlike taxa such as *Tapuiasaurus* [22,23]. Although the left quadrate of AODF 0906 is broken, the posterior surface is clearly characterized by a deep excavation (figure 13*c,i,j*), as in all macronarians [8,95]. In AODF 0906, this fossa faces posterolaterally, distinguishing it from most sauropods, with the exception of several titanosaurs, including AODF 0836, *Nemegtosaurus*, *Rapetosaurus*, *Sarmientosaurus* and *Tapuiasaurus* [17,20,22,23,41,104]. It seems unlikely that the quadrate contacted the basal tuber, distinguishing AODF 0906 from *Nemegtosaurus*, *Quaesitosaurus*, *Rapetosaurus* and *Tapuiasaurus* [17,20,22,104].

The head of the quadrate, and therefore the squamosal contact, is largely missing. However, the articular surface for the quadratojugal is complete and well-preserved, such that a subtle horizontal ridge on the quadrate (figure 13*h*) is contiguous with a similar horizontal ridge on the quadratojugal when the two elements are articulated. A comparable ridge appears to be present on each quadrate of AODF 0836, although this feature was not identified in the original description of that specimen [41]. We consider this feature to be autapomorphic for *Diamantinasaurus matildae*. Ventral to the anterior flange, the lateral surface of the quadrate is strongly concave (figure 13*b,i*), whereas the medial surface is correspondingly strongly convex (figure 13*d,k*). The ventral process of the quadrate is expanded mediolaterally and laterally bevelled at a 45° angle. The ventral articular facet, in distal view, is crescentic. As in AODF 0836, the concave margin of the facet faces anterolaterally, whereas the convex one faces posteromedially [41].

### 5.11. Pterygoid

**Figure 14.**
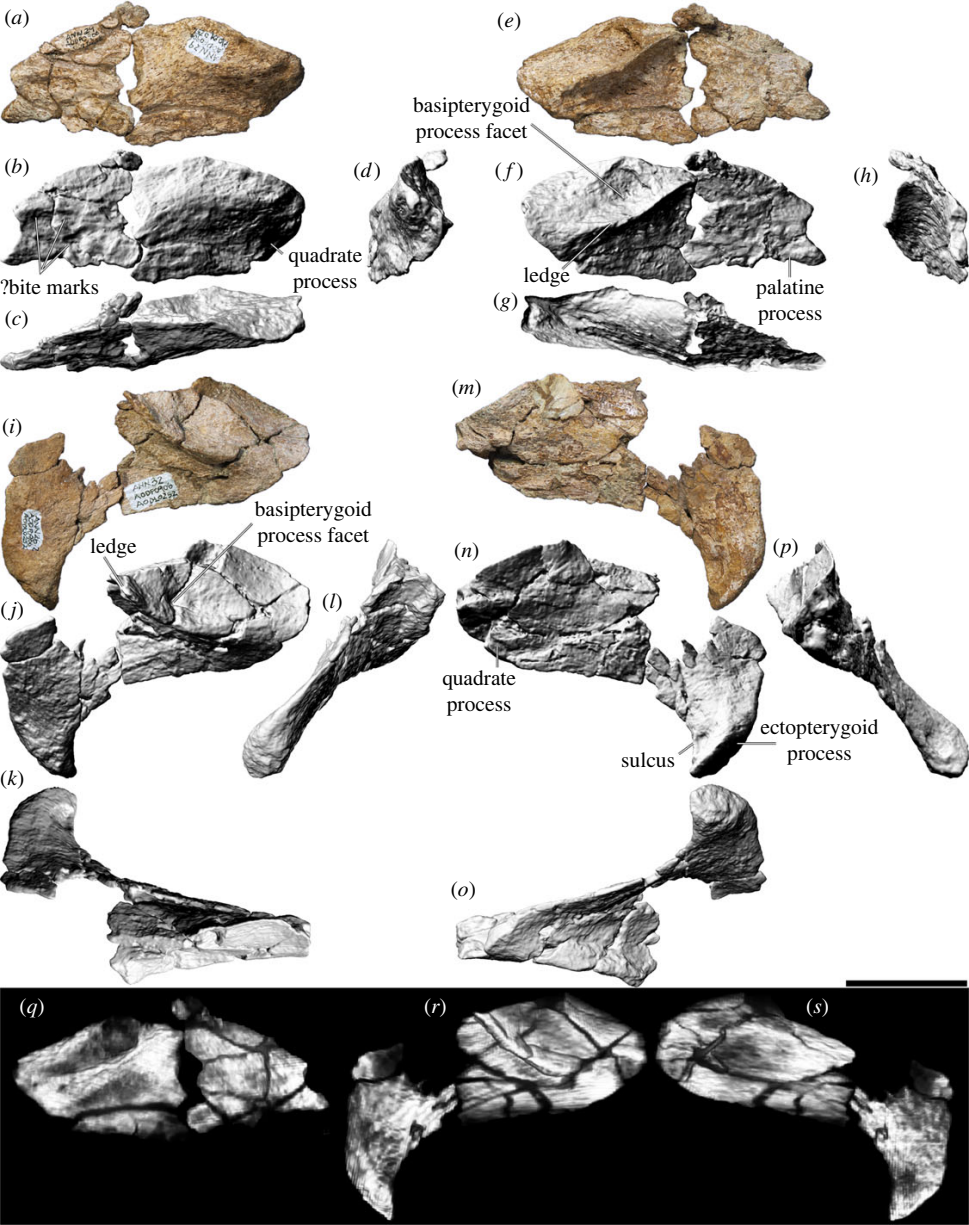
*Diamantinasaurus matildae* referred pterygoids (AODF 0906). (*a–h*) Left pterygoid in (*a*,*b*) medial, (*c*) dorsal, (*d*) posterior, (*e*,*f*) medial, (*g*) ventral and (*h*) anterior views. (*i–p*) Right pterygoid in (*i*), (*j*) medial, (*k*) dorsal, (*l*) anterior, (*m,n*) lateral, (*o*) ventral and (*p*) posterior views. (*a*), (*e*), (*i*) and (*m*) are photographs, and (*b–d*), (*f–h*), (*j–l*) and (*n*–*p*) are three-dimensional models derived from surface scans. (*q*) Three-dimensional model (derived from CT scans) of the left pterygoid in medial view. (*r,s*) Three-dimensional models (derived from CT scans) of the right pterygoid in (*r*) medial and (*s*) lateral views. Scale bar = 50 mm.

The left pterygoid (figure 14*a–h,q*) is represented by incomplete palatine and quadrate processes, whereas the right pterygoid (figure 14*i–p,r,s*) preserves nearly complete ectopterygoid and quadrate processes. Between the two elements, an almost complete pterygoid can be reconstructed, and morphologically it appears to be similar to those of *Europasaurus* [119], *Giraffatitan* [25] and *Sarmientosaurus* [23]. When complete, the pterygoid would have been triradiate, with the quadrate process directed posterolaterally, the palatine process anteriorly, and the ectopterygoid process ventrolaterally. Thus, the pterygoid of AODF 0906 was fairly robust, unlike those of *Nemegtosaurus*, *Quaesitosaurus* and *Rapetosaurus*, wherein the three processes are more or less coplanar [17,20,38]. The preserved portion of the palatine process (figure 14*e,f*) is sheet-like, being exceedingly thin mediolaterally and tall dorsoventrally. It is otherwise uninformative, although it appears to preserve bite marks (figure 14*a,b*). The distal end of the ectopterygoid process is mediolaterally thickened on its anterior surface (figure 14*l*) and tapers to a thin ridge posteriorly (figure 14*p*). This ridge is confluent with the ventral margin of the quadrate process, which is similarly mediolaterally thin along its ventral surface (figure 14*g,q*). In anterior view, the distal end of the ectopterygoid process is mostly flat. The medial surface of the ectopterygoid process is similarly flattened (figure 14*i.j*), whereas its lateral surface is mostly convex (figure 14*m,n*). The exception to this is a small sulcus immediately proximal to the end of the process, situated at the ventrolateral margin. Whether or not this is a natural feature is unclear. As is the case in most sauropods for which this can be assessed, the articular surface for the ectopterygoid appears to extend along the dorsal half of the lateral surface of the ectopterygoid process; this distinguishes AODF 0906 from *Rapetosaurus*, *Sarmientosaurus* and *Tapuiasaurus*, wherein the articular surface for the ectopterygoid is restricted to the tip of this process [21,23,38]. The lateral surface of the quadrate process is mostly dorsoventrally convex, with a shallow, horizontal rise at one-quarter of the height of the process. This implies that the medial surface of the pterygoid flange of the quadrate, which is incompletely preserved, was dorsoventrally concave. The medial surface of the quadrate process of the pterygoid is more complex (figure 14*e,f, i,j*): it is bisected by a prominent, medially projecting and anterodorsally–posteroventrally inclined ledge. The articular surface for the basipterygoid process is manifested as a socket in AODF 0906, distinguishing it from *Nemegtosaurus*, *Quaesitosaurus*, *Rapetosaurus* and *Tapuiasaurus*, wherein it forms a convex rocker-like surface [17,20,22,104]. Anteroventral to the ledge, the pterygoid is vaulted to form the posterior portion of the palate.

### 5.12. Ectopterygoid

**Figure 15.**
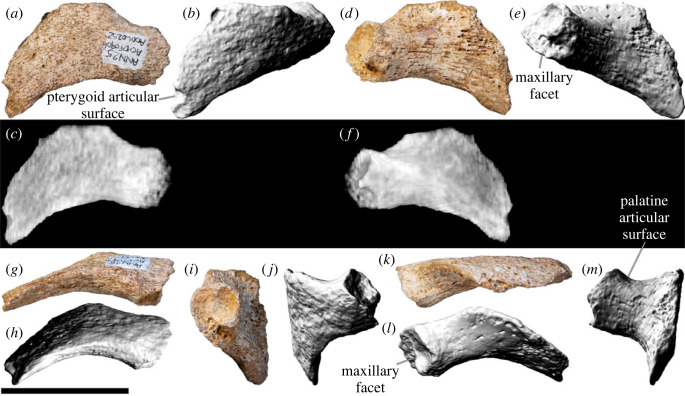
*Diamantinasaurus matildae* referred left ectopterygoid (AODF 0906). (*a–m*) Left ectopterygoid in (*a–c*) anterior, (*d–f*) posterior, (*g*,*h*) ventral, (*i*,*j*) lateral, (*k*,*l*) dorsal and (*m*) posterior views. (*a*), (*d*), (*g*), (*i*) and (*k*) are photographs; (*b*), (*e*), (*h*), (*j*) and (*l–m*) are three-dimensional models derived from surface scans; and (*c*) and (*f*) are three-dimensional models derived from CT scans. Scale bar = 50 mm.

The left ectopterygoid is virtually complete (figure 15), although the maxillary articular surface is incompletely exposed and appears to be broken. In anterior and posterior views, the ectopterygoid is ligulate (strap-shaped), being somewhat flared medially. The maxillary articular surface is circular and mostly concave, albeit partially bisected by a bulge. As in all neosauropods, the ectopterygoid did not articulate with the jugal [106]. Medial to the maxillary facet, the body of the ectopterygoid is tubular. Further medially, however, it flattens out to form a dorsoventrally expanded flange. This flange is biased posteriorly, such that the posterior surface of the ectopterygoid is essentially a mildly dorsoventrally convex sheet. The anterior surface is more strongly convex dorsoventrally than the posterior one, with a distinct dorsal shelf immediately medial to the maxillary articular facet. The medial flange is presumed to have articulated with the ectopterygoid process of the pterygoid posteriorly and the palatine anteriorly.

### 5.13. Braincase

**Figure 16.**
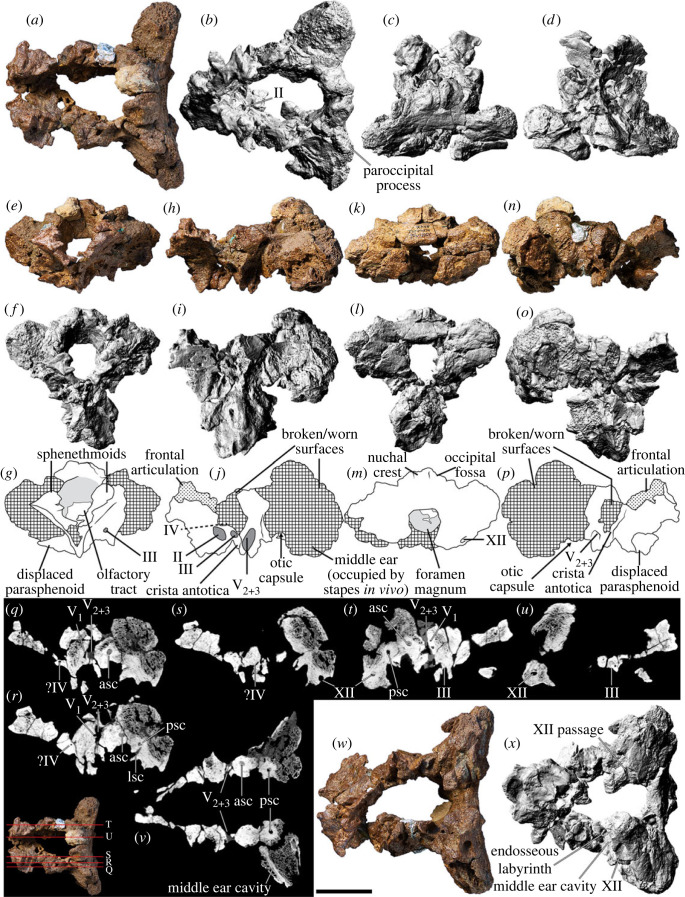
*Diamantinasaurus matildae* referred braincase (AODF 0906). (*a–p*) Braincase in (*a*,*b*) dorsal, (*c*) posterodorsal, (*d*) anteroventral, (*e–g*) anterior, (*h–j*) left lateral, (*k–m*) posterior and (*n–p*) right lateral views. (*q–u*) Braincase cross-sections in (*q–s*) left lateral, (*t*,*u*) right lateral and (*v*) dorsal views. (*w–x*) Braincase in ventral view. (*a*), (*e*), (*h*), (*k*), (*n*) and (*w*) are photographs; (*b–d*), (*f*), (*i*), (*l*), (*o*) and (*x*) are three-dimensional models derived from surface scans; and (*q–v*) are CT slices. Scale bar = 50 mm.

The braincase is poorly preserved and in two sections (figure 16). The first comprises an incomplete supraoccipital, partial otoccipitals, fragmentary prootics, laterosphenoids and orbitosphenoids. The second comprises a partial basioccipital and basisphenoid. The photographs (figure 16*a,e,h,k,n* and *w*), line drawings (figure 16*g,j,m* and *p*), and CT slices (figure 16*q–v*) of the braincase presented herein only illustrate the first fragment, whereas the screenshots of three-dimensional models derived from surface scans (figure 16*b–d, f, i, l, o* and *x*) illustrate both fragments in approximate life position.

#### 5.13.1. Supraoccipital

Despite being incomplete, the supraoccipital is one of the better-preserved braincase elements. This subhexagonal element is slightly taller dorsoventrally (greater than 32 mm) than it is wide transversely (28 mm) and forms the dorsal margin of the foramen magnum (figure 16*k–m*). As is also the case in AODF 0836 and most other sauropods, the supraoccipital is slightly taller dorsoventrally (approx. 38 mm) than the foramen magnum, which contrasts with the titanosaurs *Malawisaurus*, *Pitekunsaurus macayai* and *Rapetosaurus* [101,104]. The nuchal crest, which runs dorsoventrally along the midline of the supraoccipital, is prominent anteriorly but fades out well before reaching the level of the foramen magnum; consequently, the 20 mm tall portion of the supraoccipital dorsal to the foramen magnum is almost entirely flat. Only the left half to two-thirds of the nuchal crest is preserved, and it is difficult to determine whether or not an anteroposterior groove was present on its dorsal surface. A hint of such a groove is preserved, although this might be an artefact of preservation and is situated too far to the left to be considered ‘midline’. Although it is possible that there were two grooves on the nuchal crest, situated either side of the midline, this would be a highly unusual morphology for a sauropod, let alone a titanosauriform [150]. On both sides, lateral to the nuchal crest, the supraoccipital hosts a shallowly concave occipital fossa; however, both fossae might appear shallower than they were *in vivo* owing to their incompleteness.

#### 5.13.2. Basioccipital

The basioccipital is incomplete: the occipital condyle has been lost, and the basal tubera are extremely fragmentary. Consequently, much of the morphology of the basioccipital cannot be determined, other than that it was sutured dorsolaterally with the otoccipital, dorsally with the prootic, and anteriorly with the basisphenoid.

#### 5.13.3. Basisphenoid

The basisphenoid forms the floor of the endocranial cavity and is firmly sutured to the (missing) parasphenoid anteriorly, the orbitosphenoid anterodorsally, the laterosphenoid dorsally, the prootic posterodorsally and the basioccipital posteriorly. The basisphenoid forms the basipterygoid processes and a significant portion of the basal tubera. Unfortunately, both the basipterygoid processes and basal tubera of AODF 0906 are quite incomplete.

The hypophyseal extension is visible in dorsal view (figure 16*b*). The boundaries of this structure define an oval shape, lateral to which the paths of the carotid arteries can be traced. Although both are mostly open, this is almost certainly a consequence of breakage since finished bone is not evident throughout much of the length of the more complete left carotid artery canal. It seems likely that the carotid arteries opened lateral to the basipterygoid processes, as in most sauropods, including AODF 0836 [41] and *Sarmientosaurus* [23], but contrasting with the condition in several titanosaurs (e.g. *Antarctosaurus*, *Nemegtosaurus*, *Rapetosaurus*, *Saltasaurus*), as well as the early-branching somphospondylan *Tambatitanis amicitiae* [151] and possibly *Mongolosaurus haplodon* ([152]: fig. 2), in which the carotid arteries exit medial to the basipterygoid processes [38,153].

#### 5.13.4. Otoccipital

As in sauropods generally, the exoccipital and opisthotic are firmly sutured [8]; consequently, they are referred to herein as the otoccipital. The otoccipital is dorsomedially sutured to the supraoccipital, ventromedially to the basioccipital, anteroventrally to the prootic, and anteromedially to the laterosphenoid. The medial edge of the otoccipital forms the lateral margin of the foramen magnum. Immediately lateral to the foramen magnum lies the external opening for the hypoglossal nerve (CN XII). A single exit for CN XII is present on each side, as in most macronarians [38,153], including AODF 0836 [38,41]. This contrasts with *Sarmientosaurus* [23], as well as some specimens of *Camarasaurus* and *Giraffatitan*, which have two openings for CN XII on each side [153]. In AODF 0906, the left CN XII opening is broken, such that its passage from the endocast to the anterolateral surface of the occipital condyle is entirely exposed. By contrast, the right CN XII canal is intact, such that only its internal and external openings can be observed. Lateral to the left side of the foramen magnum, and dorsal to the external opening for CN XII, a node-like proatlantal facet appears to be present; a less well-preserved one appears to be present on the right side as well. Proatlantal facets have also been identified in AODF 0836 [41] and characterize most titanosaurs, although they are absent in *Malawisaurus*, *Rapetosaurus* and *Sarmientosaurus* [23,38,41]. When complete, the otoccipitals would have formed prominent paroccipital processes laterally; however, both are incomplete laterally, and the morphology of the paroccipital processes is unclear.

#### 5.13.5. Prootic

Both prootics are incomplete in AODF 0906. Each prootic is firmly wedged between the laterosphenoid anteriorly, the parietal dorsally, the supraoccipital posteriorly, the opisthotic portion of the exoccipital-opisthotic complex posteroventrally, and the basisphenoid and basioccipital ventrally. The crista antotica marks the boundary between the laterosphenoid and prootic. Immediately posterior to this ridge is a foramen that would have hosted the maxillary (CN V_2_) and mandibular (CN V_3_) branches of the trigeminal nerve (CN V). This section of the prootic is poorly preserved on both sides of AODF 0906; nevertheless, it is clear that the morphology of this region is extremely similar to that of AODF 0836 [38,41]. Little evidence of the crista prootica is preserved on either prootic. Consequently, the precise position of the opening for the facial nerve (CN VII) is difficult to determine.

Neither prootic preserves its parietal or basisphenoid suture, meaning that the crista tuberalis is not preserved. By contrast, the crista interfenestralis is preserved on both sides. In AODF 0836, this feature was interpreted as a preservational artefact [38,41]; however, the fact that this structure in AODF 0906 occupies exactly the same position and has a near-identical morphology implies that it is a genuine anatomical feature. Posterior to the crista interfenestralis lies the metotic fissure. Anterior to the crista interfenestralis, the dorsal surface of a straight, narrow, posterolaterally–anteromedially oriented canal—the middle ear canal (or tympanic cavity)—can be observed: *in vivo*, this would have been occupied by the stapes (figure 16*w–x*). Medial to this canal, the matrix infilling the cochlear portions of both endosseous labyrinths has been removed, approximately to the level of the lateral semicircular canal; it is also possible to discern the approximate paths of the anterior and posterior semicircular canals. CT data reveal that the anterior semicircular canal is slightly larger in diameter than the posterior one (figure 16*q,r, t* and *v*). The anterior and posterior semicircular canals diverge from each other at approximately 100°, as in AODF 0836 [41].

#### 5.13.6. Laterosphenoid

The anterolaterally concave laterosphenoid forms the posteromedial rim of the orbital fossa. Neither the dorsal suture with the frontal, nor the lateral suture with the postorbital, can be observed; by contrast, the completely preserved anterior suture with the orbitosphenoid has been largely obliterated. The orbitosphenoid suture line of the laterosphenoid is punctuated by the openings for the oculomotor (CN III), trochlear (CN IV) and abducens (CN VI) nerves, as in sauropods generally [154]. The laterosphenoid is sutured to the opisthotic portion of the otoccipital posteriorly, and to the prootic posteroventrally. The contact between the laterosphenoid and otoccipital/prootic is manifested as the crista antotica. The ophthalmic branch (CN V_1_) of the trigeminal nerve (CN V) lies entirely on the laterosphenoid, anterior to the crista antotica; the other two branches of the trigeminal nerve exited posterior to the crista antotica, as in AODF 0836 [38,41]. Among neosauropods, only *Phuwiangosaurus*, *Diamantinasaurus* and *Sarmientosaurus* are characterized by more than one ossified exit for CN V [23,38,41,82,149].

#### 5.13.7. Orbitosphenoid

Both orbitosphenoids are incomplete. They are firmly sutured together along their ventral margins, and each is sutured posteriorly to its corresponding laterosphenoid. The frontal articulation, which would have been situated dorsally, is indistinct on both orbitosphenoids; however, based on the left side, it would seem that this suture was interdigitated, as in AODF 0836 [38,41]. The suture with the basisphenoid is poorly preserved on both.

The opening for the olfactory nerve (CN I) is situated anteriorly and is generally well preserved. A medially projecting prong is present within this opening on the right orbitosphenoid; a similar structure is present in the left orbitosphenoid of AODF 0836, and tentatively interpreted to represent the boundaries of the olfactory filaments [38,41]. One of few other titanosaurs in which the anteriormost portion of the orbitosphenoid is preserved, and in which the braincase was found disarticulated from the rest of the skull, is *Bonatitan reigi*: in this taxon, ethmoidal elements (specifically sphenethmoids) were inferred to be preserved anterior to, and in articulation with, the orbitosphenoids [155]. If this interpretation is correct, then at least one sphenethmoid appears to be preserved in AODF 0836, and both appear to be preserved in AODF 0906. The sphenethmoid would then be the element that hosts the medially projecting prong described above. The opening for the optic nerve (CN II) is hosted on the orbitosphenoid and is medially divided, as in nearly all neosauropods [156,157]. The opening for CN III is positioned immediately posterior to that for CN II, whereas the section that would have hosted CN IV (posterodorsal to CN II) is incomplete on both sides (although its passage can possibly be traced using the CT data, at least on the left side (figure 16*q,r*)). The passage for CN VI appears to be preserved on the left side (figure 16*s*). As in AODF 0836 [38,41], each CN VI canal projects lateral to the hypophyseal chamber and does not penetrate the pituitary fossa, meaning that *Diamantinasaurus* shows the derived condition that characterizes all titanosaurs [6,38,153]. In AODF 0906, as in many titanosaurs (including AODF 0836), the opening for CN VI is interpreted to have been situated anteroventral to, and quite distant from, the opening for CN III; by contrast, in non-titanosaurian macronarians, the external opening for CN VI lies ventral (and often quite close) to that for CN III [6,38,41,158].

### 5.14. Dentary

**Figure 17.**
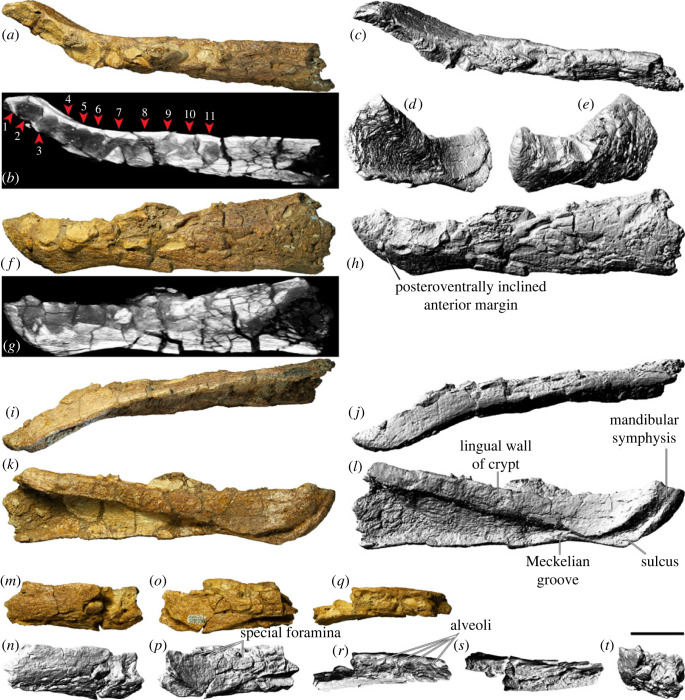
*Diamantinasaurus matildae* referred dentaries (AODF 0906). (*a–l*) Left dentary in (*a–c*) occlusal, (*d*) posterior, (*e*) anterior, (*f–h*) lateral, (*i–j*) ventral and (*k–l*) medial views. (*a*), (*f*), (*i*) and (*k*) are photographs; (*b*) and (*g*) are three-dimensional models derived from CT scans; and (*c–e*), (*h*), (*j*) and (*l*) are three-dimensional models derived from surface scans. Numbers 1–11 indicate alveoli. (*m–t*) Right dentary in (*m–n*) lateral, (*o–p*) medial, (*q–r*) occlusal, (*s*) ventral and (*t*) posterior views. (*m*), (*o*) and (*q*) are photographs, (*n*), (*p*) and (*r–t*) are three-dimensional models derived from surface scans. Scale bar = 50 mm.

The left dentary is nearly complete and anteroposteriorly elongate (figure 17*a–l*); by contrast, the right dentary is represented only by a fragment (figure 14*m–t*). The description below is based on the left dentary unless otherwise indicated.

The dorsoventral height of the anterior end of the dentary (51 mm) is approximately equivalent to its height at mid-length (49 mm), as in most titanosauriforms, other than *Camarasaurus*, brachiosaurids and *Malawisaurus*, wherein the anterior end of the dentary is 120% taller than the mid-length [6,95,100,104]. As preserved, the anterior margin of the dentary is angled posteroventrally (figure 17*f–h*), as is the case in most sauropods [38,100], including *Camarasaurus* [118], *Europasaurus* [119], *Euhelopus* [114], and the titanosaurs *Malawisaurus* [115] and *Choconsaurus baileywillisi* [159]. This distinguishes AODF 0906 from brachiosaurids and most titanosaurs, including *Mansourasaurus shahinae*, *Nemegtosaurus*, *Rapetosaurus*, *Sarmientosaurus* and *Tapuiasaurus*, wherein the anterior margin is perpendicular (or nearly so) to the long axis of the dentary [17,20,22,23,26,160]. The ‘chin' observed in *Mansourasaurus* [160] and many flagellicaudatans [100,161] is not present in AODF 0906, nor is there a labial tuberosity near the anterior end of the dentary, such as that which characterizes dicraeosaurids [131].

The paired dentaries would have formed a rounded lower jaw in occlusal view (figure 17*a–c*), as in non-titanosauriform macronarians, brachiosaurids, *Euhelopus*, and numerous titanosaurs, including *Ampelosaurus atacis*, *Choconsaurus*, *Karongasaurus gittelmani*, *Malawisaurus*, *Mansourasaurus*, *Nemegtosaurus*, *Quaesitosaurus*, *Rapetosaurus*, *Sarmientosaurus* and *Tapuiasaurus* [2]. This is in stark contrast to the condition in some other titanosaurs, such as *Antarctosaurus*, *Baalsaurus mansillai*, *Bonitasaura* and *Brasilotitan nemophagus*, wherein the dentaries are squared-off in occlusal view [141,145,162–165].

The lateral surface of the left dentary (figure 17*a–c*), which is dorsoventrally convex along its length, has suffered extensive fragmentation. As a result, the tooth row appears to have been bowed outwards relative to the rest of the dentary, as in dicraeosaurids, *Nigersaurus taqueti* [166], as well as *Antarctosaurus* [90]. However, the preserved portion of the right dentary demonstrates that this was not the case *in vivo*—as in other macronarians, the lingual and labial margins of the alveoli were level, and the tooth row was not bowed either way (figure 17*q,r*). Presently, it is not possible to determine if the dentary of AODF 0906 bifurcated into two posterior processes (dorsal and ventral), nor if the posteroventral process was forked (as it is in brachiosaurids [26]).

The medial surface of the dentary (figure 17*k,l*) can be divided into four surfaces. The anteriormost and least extensive is the flattened articular surface for the opposite dentary. Immediately ventral to the mandibular symphysis, a distinct sulcus is present. Although it is separated from the anterior end of the Meckelian groove by a region wherein the lateral and medial surfaces meet directly (approx. 30 mm long anteroposteriorly), such that they are barely separated by a groove (in the left dentary) or entirely lack a groove (in the right one), it is possible that this feature represents an anterior continuation of the Meckelian groove proper. Among macronarians, this feature appears to be present only in ontogenetically immature individuals of the early-diverging macronarian *Europasaurus* [119]. Thus, this feature might indicate that the AODF 0906 individual was not fully grown perimortem; alternatively, it might be locally autapomorphic for *Diamantinasaurus* within Titanosauria or even Titanosauriformes. The most extensive of the four medial surfaces of the dentary is the lingual wall of the crypt (within which the replacement teeth were generated and held). This surface is mostly flat, except where it is concave immediately posterior to the dentary articulation. This is also the position at which this surface is dorsoventrally tallest: it gradually decreases in dorsoventral height posteriorly (owing to the Meckelian groove) and anteriorly (owing to the anteroventral sulcus). Both dentaries preserve the ventral margins of several special foramina, although those on the less complete right dentary are more easily observable. Posteroventral to the crypt lies the Meckelian groove, which is dorsoventrally concave, anteriorly tapered and posteriorly expanded. The medial surface of the dentary within the Meckelian groove is concave and would have been overlain by the splenial in life.

CT scan data reveal that the incomplete right dentary of AODF 0906 hosts six alveoli, whereas the complete toothrow of the left dentary comprises 11 alveoli (figure 17*a,g*). The presence of 11 dentary alveoli separates AODF 0906 from non-titanosaurian titanosauriforms, which typically have 12 to 14 [24–26,114,118,119,133,135,137,167]. It also separates AODF 0906 from several titanosaurs with rounded snouts, including *Nemegtosaurus*, *Quaesitosaurus* and *Sarmientosaurus*, which have 13 [18–20,23]. However, alveolus count in titanosaurs with rounded snouts varies greatly. Some of the lowest counts are those seen in *Ampelosaurus* (nine) and *Mansourasaurus* (10) [160,168]. *Rapetosaurus* possesses at least 11 [17], and *Karongasaurus* at least 12 [115]. By contrast, other titanosaurs with rounded snouts have a greater number of alveoli, with 15 in *Tapuiasaurus* [22] and at least this many in *Malawisaurus* [115]. Similarly, alveolus counts in titanosaurs with squared-off snouts vary, from at least 13 in *Baalsaurus* [165], to 14 in *Brasilotitan* [164] and 16 in *Antarctosaurus* (MACN 6904, S.F.P. and P.D.M., pers. obs.). This places AODF 0906 at the lower end of the expected range of dentary alveoli for Titanosauria, particularly among early-branching titanosaurs.

### 5.15. Surangular

**Figure 18.**
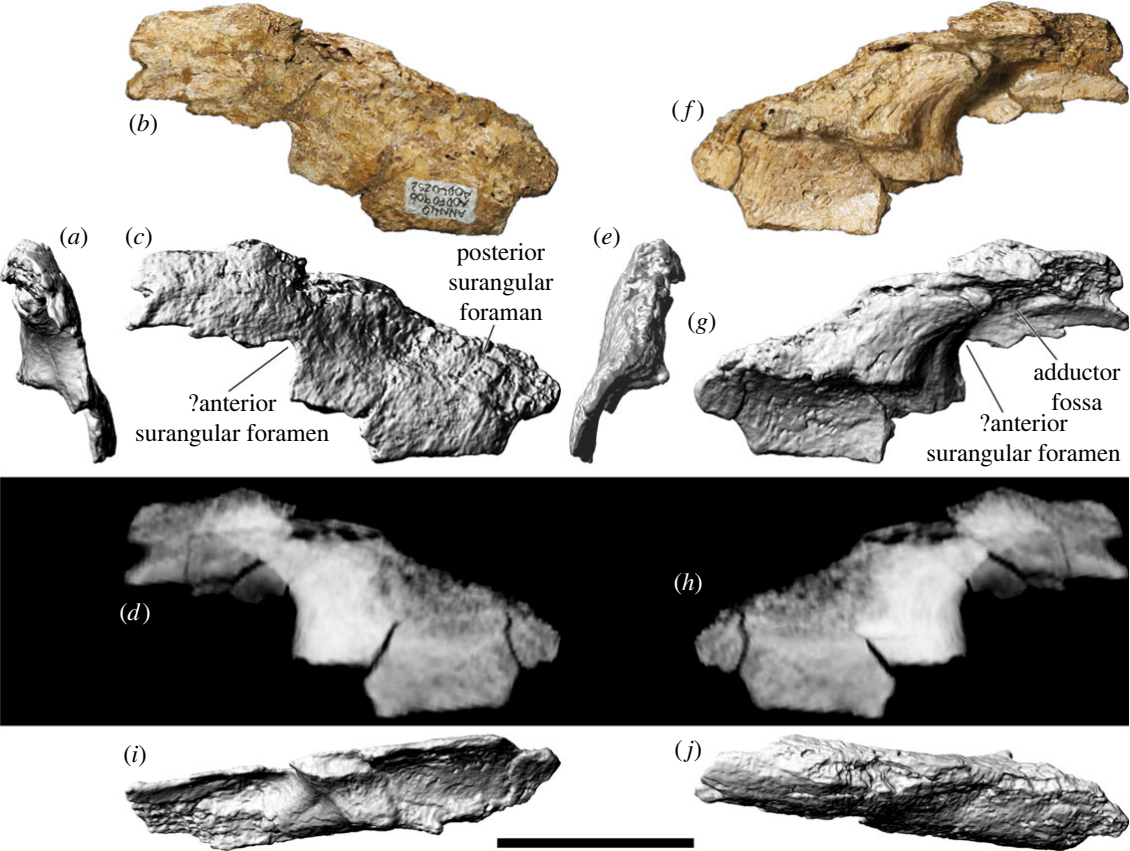
*Diamantinasaurus matildae* referred left surangular (AODF 0906). (*a–j*) Left surangular in (*a*) anterior, (*b–d*) lateral, (*e*) posterior, (*f–h*) medial, (*i*) ventral and (*j*) dorsal views. (*a*), (*c*), (*e*), (*g*) and (*i–j*) are three-dimensional models derived from surface scans; (*b*) and (*f*) are photographs; and (*d*) and (*h*) are three-dimensional models derived from CT scans. Scale bar = 50 mm.

The left surangular (figure 18) is almost identical to, and more completely preserved than, the surangular of AODF 0836 [41]. It is also broadly similar to the surangulars of *Giraffatitan* [25] and *Sarmientosaurus* [23]. The surangular of AODF 0906 is mediolaterally thicker dorsally (figure 18*j*) than ventrally (figure 18*i*). The undulating dorsal margin (which is the only complete margin) is shallowly convex anteriorly and shallowly concave posteriorly. The preserved portion of the lateral surface of the surangular (figure 18*b,c*) is broadly convex, but shallowly concave posterodorsally. This part of the surangular in *Tapuiasaurus* accommodated the anteroventral prong of the quadratojugal [22]; however, the quadratojugal of AODF 0906 lacks this feature. As in AODF 0836, the dorsal portion of the medial surface of the surangular of AODF 0906 is manifested as a distinct double arch (figure 18*f,g*). The anterior arch is incomplete anteromedially, whereas the posterior arch is almost complete. Ventral to each arch, the medial surface of the surangular is shallowly concave. The anterior concavity (the adductor fossa) appears to preserve the posterior margin of a fairly large foramen. This appears to be homologous with, albeit smaller than, the anterior surangular foramen present in the surangulars of *Nemegtosaurus* [18,20] and *Rapetosaurus* [17]. The surangulars of *Abydosaurus*, *Euhelopus*, *Europasaurus*, *Giraffatitan*, *Quaesitosaurus*, *Sarmientosaurus* and *Tapuiasaurus* lack an enlarged surangular foramen, instead having a much smaller anterior surangular foramen, situated further anteriorly [19,22,23,25,114,119]. The morphology of this foramen varies in *Camarasaurus*: an enlarged foramen is present in some exemplars, whereas in others a smaller foramen is evident [118]. CT scan data of *Sarmientosaurus* reveal that the anterior surangular foramen connects to a posteromedially projecting canal, which expands greatly (diameter approx. 10 mm) within the body of the surangular, and exits on the medial surface near the posterior margin of the adductor fossa [23]—the precise position at which this foramen is located in AODF 0906 (and, evidently, in AODF 0836). It is likely, then, that these foramina are homologous. In living reptiles, the anterior and posterior surangular foramina accommodate cutaneous branches of the inferior alveolar nerve [169,170].

### 5.16. Teeth

No loose teeth were found at the AODL 0252 site, and no active teeth remained within the preserved dentigerous elements. Computed tomography (CT) scan data revealed that the premaxilla and maxilla preserve replacement teeth, but that the dentaries do not. In the maxilla, the few preserved replacement teeth are small, situated nearer the base of the crypt than the external margins of their alveoli, only present in the distalmost alveoli, and barely distinguishable from the bone of the maxilla. By contrast, the premaxillary replacement teeth are large, some are close to eruption, and all are clearly differentiable from the surrounding bone (figure 4*j–l*).

**Figure 19.**
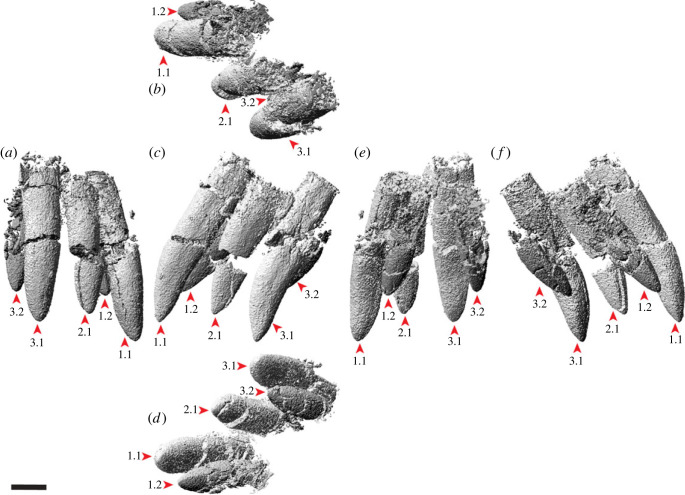
*Diamantinasaurus matildae* referred premaxillary replacement teeth (AODF 0906). (*a–f*) Three-dimensional models of the left premaxillary replacement teeth (derived from synchrotron scan data) in (*a*) anterior, (*b*) dorsal, (*c*) lateral, (*d*) ventral, (*e*) posterior and (*f*) medial views. The number preceding the period (1–3) indicates the alveolus position; the number succeeding the period (1 or 2) indicates the position of that tooth within the alveolus, with 1 being more labial than 2. Scale bar = 10 mm.

**Figure 20.**
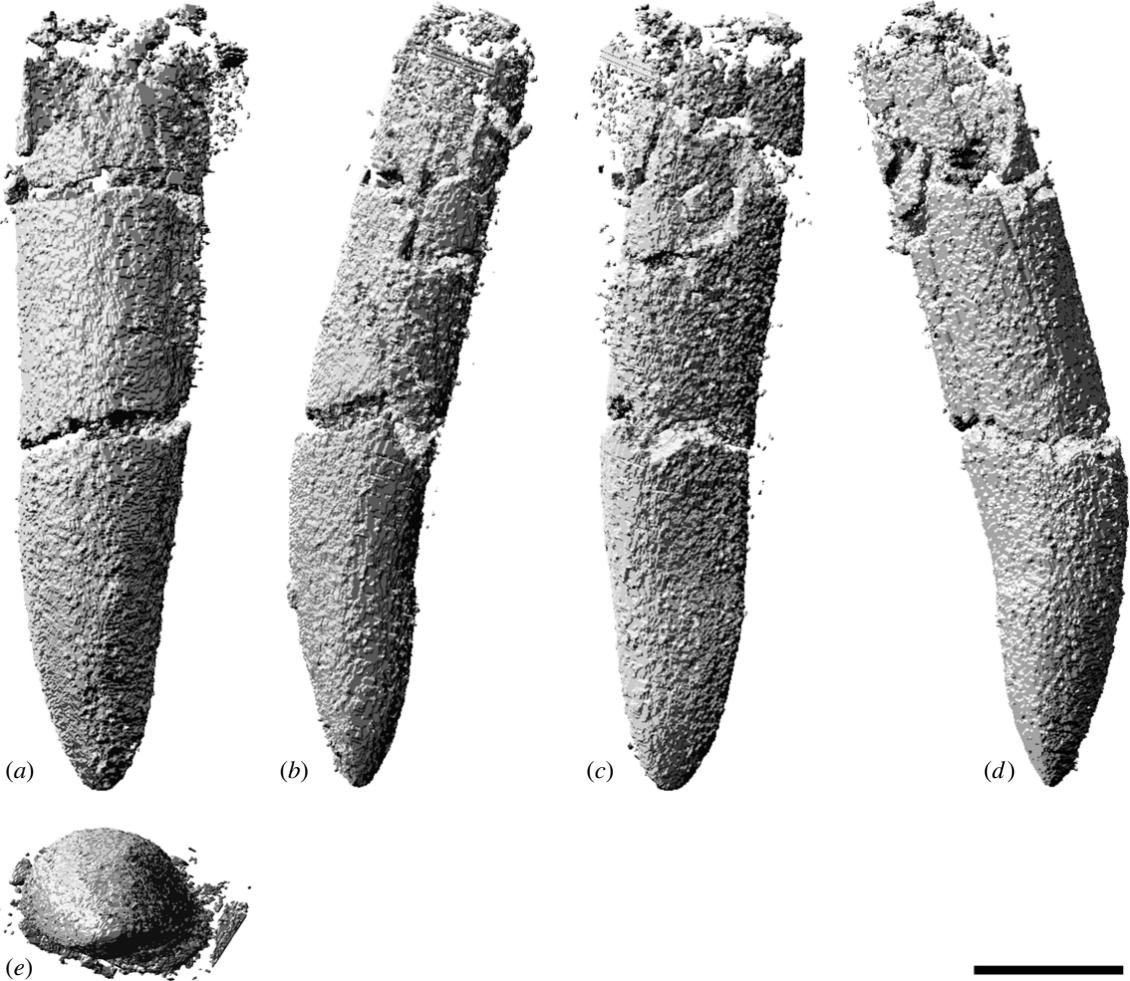
*Diamantinasaurus matildae* referred first premaxillary replacement tooth (AODF 0906). (*a–e*) Three-dimensional models of the left premaxillary replacement tooth 1.1 (derived from synchrotron scan data) in (*a*) labial, (*b*) distal, (*c*) lingual, (*d*) mesial and (*e*) apical views. Scale bar = 10 mm.

As outlined above, five replacement teeth are preserved within the left premaxilla of AODF 0906: two in the first alveolus, one in the second, and two in the third (figure 19). Thus, as in all sauropods other than diplodocoids, there were three or fewer replacement teeth per alveolus in AODF 0906 [104]. This description is based primarily on the largest replacement teeth present: the labialmost tooth in each of the first (figure 20) and third alveoli.

The teeth within the premaxilla of AODF 0906 are almost identical to those from the *Diamantinasaurus matildae* type (AODL 0085) and referred specimen (AODL 0127) sites, and those from the ‘Mitchell' site (AODL 0270; [43]). They also show some similarity to some of the sauropod teeth described from Lightning Ridge [33,35]. The crown of each tooth of the AODF 0906 premaxilla is tapered apically, but otherwise has parallel sides, showing virtually no expansion above the root, as in neosauropods generally [100,171]. The cross-sectional shape of the crown of each tooth is generally D-shaped (figure 20*d*), although each becomes more rounded towards the root. The labial surface (figure 20*a*) of each tooth is generally smooth—as in titanosaurs, some early-deriving somphospondylans, and most diplodocoids—thereby contrasting with the longitudinally grooved surfaces seen in the teeth of most other sauropods [38,106]. The lingual surface (figure 20*c*) of each tooth is mostly convex, as is the case in titanosaurs generally [8,100,101]. The teeth lack an apicobasally oriented lingual ridge, as in most somphospondylans [101,172]. They also lack the bulges present towards the mesial and distal ends of the lingual surface (near the crown base), differentiating AODF 0906 from *Euhelopus* and *Yongjinglong* [113,114,173]. The teeth lack prominent mesial (figure 20*d*) and distal (figure 20*b*) carinae. Carinae characterize the teeth of most somphospondylans, although they are also absent in *Ligabuesaurus* [174], *Sauroposeidon* [175], and a small number of titanosaurs, including *Rapetosaurus* and *Sarmientosaurus* [41,101,152]. Based on the synchrotron scan data, the thickness of the tooth enamel is uniform labially and lingually. As in other somphospondylans, serrations or denticles are not evident on any of the teeth [8,101,104].

Four of the five premaxillary replacement teeth were measured (table 2), with the only exception being the severely damaged tooth in alveolus 3. The apicobasal heights of the preserved tooth crowns range from 25.40 to 28.07 mm (table 2). Thus, they are larger than all of the sauropod teeth described from the Griman Creek Formation [35], similar in size to the sauropod teeth from the sites that produced the *Diamantinasaurus matildae* holotype (AODL 0085) and referred (AODL 0127) specimens in the Winton Formation, and smaller than the sauropod teeth from AODL 0270, also in the Winton Formation [43]. The slenderness indices (SI = apicobasal length: mesiodistal width of crown ratio) of the premaxillary replacement teeth range from 2.33 to 2.74 (table 2). Similar tooth SI indices have been recorded in early-branching macronarians, brachiosaurids, most non-titanosaurian somphospondylans, and early-branching titanosaurs such as *Sarmientosaurus*, whereas much higher SI indices are evident in most titanosaurs [8,23,26,43,100,101]. The AODF 0906 teeth are generally more slender than those from the Griman Creek Formation: the SI indices of these range from 1.36 to 3.10 (mean SI = 2.09), although only four of the 26 teeth sampled have SI indices greater than 2.40 [35]. The AODF 0906 teeth are also generally slenderer than those from AODL 0270, the SI indices of which range from 2.00 to 2.88 (with six of the 10 below 2.10) [43].

**Table 2.** Measurements of the premaxillary replacement teeth of AODF 0906 *Diamantinasaurus matildae*.

premaxillary tooth	apicobasal height	mesiodistal length	labiolingual breadth	SI	CI
alveolus 1 (labialmost)	28.07	12.05	10.11	2.33	0.84
alveolus 1 (lingualmost)	25.89	9.44	8.71	2.74	0.92
alveolus 2	25.40	9.83	9.61	2.58	0.98
alveolus 3 (labialmost)	27.40	10.31	9.19	2.66	0.89

The compression indices (CI = labiolingual breadth: mesiodistal width of crown ratio; [176]) of the AODF 0906 premaxillary replacement teeth range from 0.84 to 0.98. Thus, they are more compressed than the teeth from the Griman Creek Formation: CI indices range from 0.60 to 0.91, but only four of the 26 teeth sampled have CI values exceeding 0.85 [35]. The CI indices of the sauropod teeth from AODL 0270 range from 0.71 to 1.09, with five of the 10 having CI values below 0.80; thus, the AODF 0906 teeth are generally more compressed than those from AODL 0270.

## 6. Hyobranchial apparatus

**Figure 21.**
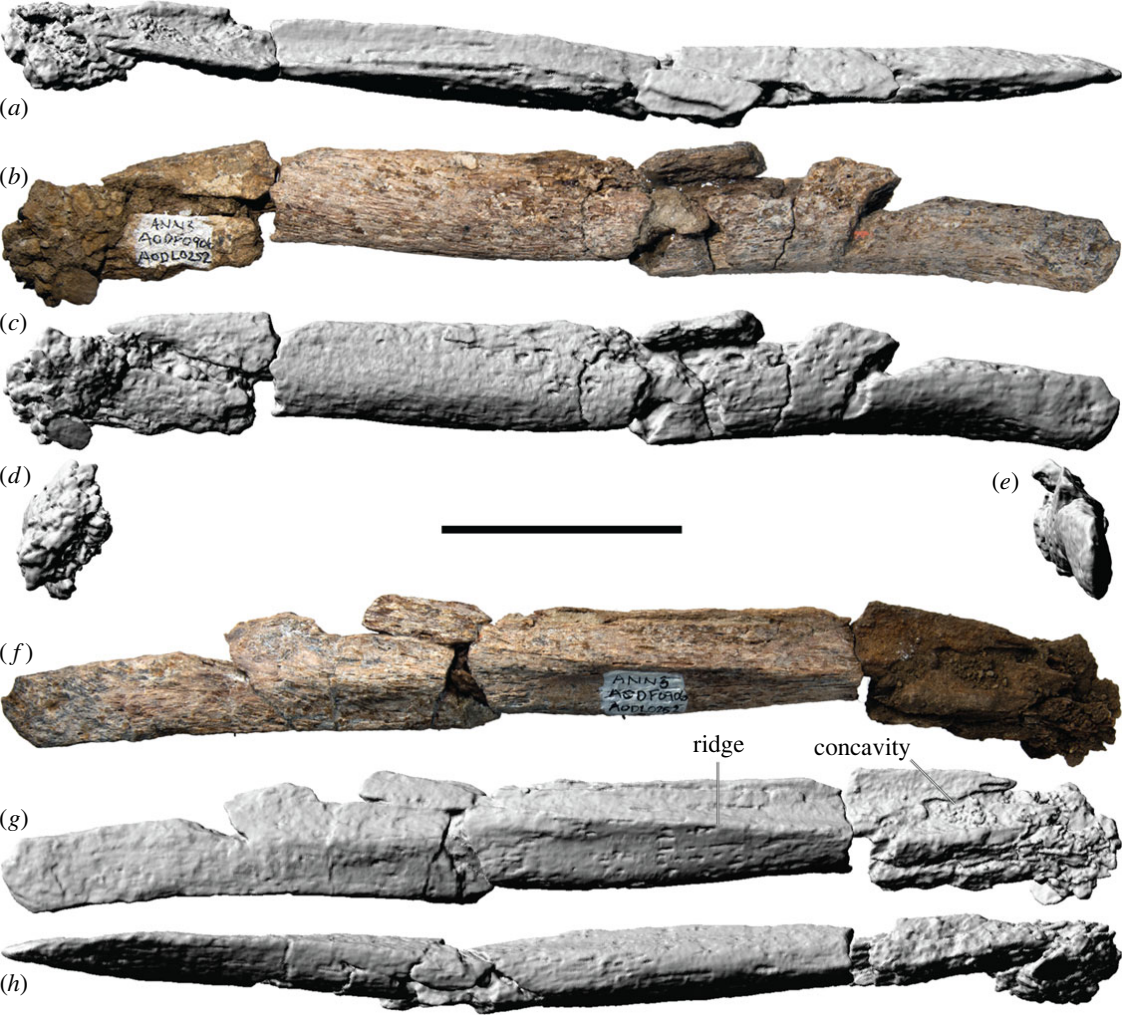
*Diamantinasaurus matildae* referred ?left ceratobranchial (AODF 0906). (*a–h*) ?Left ceratobranchial in (*a*) dorsal, (*b*,*c*) lateral, (*d*) anterior, (*e*) posterior, (*f*,*g*) medial and (*h*) ventral views. (*a*), (*c–e*) and (*g–h*) are three-dimensional models derived from surface scans; (*b*) and (*f*) are photographs. Scale bar = 50 mm.

An isolated, elongate and incomplete element (230 mm long), found associated with the left quadratojugal, appears to represent part of the hyobranchial apparatus (figure 21). It is described as if the long axis of the element runs anteroposteriorly, with the incomplete end interpreted as the anterior end and the complete end as the posterior tip, and the thinnest margin of the shaft being the dorsal one.

The incomplete anterior end is damaged (figure 21*d*), but is comma-shaped in cross-section. The dorsal margin of the shaft forms a narrow ridge along its length (figure 21*a*). The lateral surface is convex along its length (figure 21*b,c*). The medial surface (figure 21*f,g*) hosts a ridge along its midline, although this fades out towards the posterior end. Dorsal to this ridge on the anterior half, a prominent concavity is present. Ventral to this ridge, and dorsal to it on the posterior half, the medial surface is flat to slightly convex. The ventral margin is rounded except near the posterior end, where it becomes a mediolaterally narrow ridge (figure 21*h*). The complete posterior end is mediolaterally flattened (figure 21*e*).

Hyobranchial elements are not uncommonly preserved in early-branching sauropodomorphs but have relatively infrequently been identified in sauropods (table 3). Among the few sauropods for which hyoid elements have been reported, those that have been described and/or illustrated in detail pertain to the early-diverging eusauropods *Shunosaurus lii* [207] and *Omeisaurus junghsiensis* [208], the diplodocid *Galeamopus pabsti* [211], the early-diverging macronarians *Europasaurus* [119,214], *Giraffatitan* [25] and *Abydosaurus* [26], and the somphospondylans *Phuwiangosaurus* [215] and *Tapuiasaurus* [21,22]. The AODF 0906 hyoid element bears little resemblance to the slender, straight element preserved in *Abydosaurus* [26], instead showing similarities to the flared posterior ends of the boomerang-like hyoid elements seen in the other sauropods listed above. The hyoid elements preserved in these sauropods, and specifically *Tapuiasaurus*, were interpreted by Wilson *et al*. [22] as second ceratobranchials, following the interpretation of an exquisitely preserved ankylosaurid hyobranchial apparatus by Hill *et al*. [177]. However, Yoshida *et al*. [178] revised the interpretation of this ankylosaurid, claiming that the element in question is more likely to be ceratobranchial 1 than ceratobranchial 2; according to those authors, ceratobranchial 2 is commonly lost in Archosauria. Thus, we interpret the probable hyoid element in AODF 0906 as a ceratobranchial, likely ceratobranchial 1.

**Table 3.** Sauropodomorphs for which hyoid elements have been reported. If the identification by Wilson *et al*. [22] of the hyoid elements of *Tapuiasaurus macedoi* as second ceratobranchials is correct (following the interpretation of the hyobranchial apparatus of the ankylosaurid *Pinacosaurus grangeri* presented by Hill *et al*. [177]), then the same identification should also be applied to the morphologically similar hyoid elements of (at least) *Shunosaurus lii*, *Omeisaurus junghsiensis*, *Diplodocus* sp., *Galeamopus pabsti*, *Europasaurus holgeri*, *Giraffatitan brancai*, *Phuwiangosaurus sirindhornae* and *Diamantinasaurus matildae* among sauropods. By contrast, if Yoshida *et al*. [178] are correct in their reinterpretation of the hyobranchial apparatus of *Pinacosaurus grangeri*, then the identification of these as first ceratobranchials is correct; this seems likely.

taxon	published interpreted identity	reference
non-sauropodan sauropodomorphs		
*Buriolestes schultzei*	hyoid	Cabreira *et al.* [179]
	hyoid	Müller *et al.* [180]
*Pantydraco caducus*	?hyoid	Kermack [181]
	?Corpus hyoideum + cornua	Galton & Kermack [182]
*Macrocollum itaqui*	hyoid	Müller [183]
*Unaysaurus tolentinoi*	?hyoid	McPhee *et al.* [184]
*Issi saaneq*	ceratobranchial	Beccari *et al.* [185]
*Plateosaurus* spp.	illustrated but not identified	Jaekel [186]
	cornubranchial 1	Fürbringer [187]
	hyoid	Huene [188]
	hyoid	Huene [189]
	hypobranchial 1	Janensch [190]
	first ceratobranchial	Galton [191]
	ceratobranchial	Lallensack *et al.* [192]
*Lufengosaurus huenei* (=‘*Fulengia youngi*’)	ceratobranchial 1	Carroll & Galton [193]
	ceratobranchial 1	Evans & Milner [194]
*Xixiposaurus suni*	hyoid	Sekiya [195]
*Anchisaurus polyzelus*	first ceratobranchial	Galton [196]
	ceratobranchial	Fabbri *et al.* [197]
*Adeopapposaurus mognai*	ceratobranchial	Martínez [198]
*Leyesaurus marayensis*	ceratobranchial	Apaldetti *et al.* [199]
*Massospondylus carinatus*	illustrated but not identified	Gow *et al.* [200]
	ceratobranchial 1 (cornu branchial 1)	Sues *et al.* [201]
*Jingshanosaurus xinwaensis*	hyoid	Zhang & Yang [202]
*Melanorosaurus readi*	ceratohyal	Yates [203]
*Xingxiulong chengi*	ceratobranchial	Wang *et al.* [204]
	ceratobranchial	Wang *et al.* [205]
*Yunnanosaurus huangi*	ceratobranchial	Barrett *et al.* [206]
sauropods		
*Shunosaurus lii*	hyoid	Zhang [207]
*Omeisaurus junghsiensis*	hyoid	Dong *et al.* [208]
*Mamenchisaurus jingyanensis*	hyoid	Zhang *et al.* [209]
*Diplodocus* sp.	ceratobranchial	Woodruff *et al.* [210]
*Galeamopus pabsti*	ceratobranchial	Tschopp & Mateus [211]
*Brontosaurus excelsus*	two sets of hyoid bones	Marsh [212]
*Lavocatisaurus agrioensis*	hyoid	Canudo *et al.* [213]
*Europasaurus holgeri*	ceratobranchial 1 (?)	Laven [214]
	ceratobranchial 1	Marpmann *et al.* [119]
*Camarasaurus lentus*	thyrohyals	Gilmore [135]
	three fragments pertaining to two bones	Wilson *et al.* [22]
*Giraffatitan brancai*	hypobranchial 1	Janensch [190]
	hypobranchial 1	Janensch [25]
*Abydosaurus mcintoshi*	hyoid	Chure *et al.* [26]
*Phuwiangosaurus sirindhornae*	hyoid	Martin *et al.* [215]
*Diamantinasaurus matildae*	ceratobranchial 1	this paper
*Tapuiasaurus macedoi*	hyoid	Zaher *et al.* [21]
	ceratobranchial 2	Wilson *et al.* [22]

## 7. Postcranial axial skeleton

### 7.1. Dorsal ribs

The four dorsal ribs that comprise part of AODF 0906 are poorly preserved and incomplete. Whereas all lack their proximal ends, some preserve enough of their distal ends to demonstrate that they were plank-like, as in all titanosauriforms [104], including all Australian Cretaceous sauropod taxa for which ribs are known: *Austrosaurus mckillopi* [39], *Wintonotitan* [9,36], *Diamantinasaurus* [9,39,44] and *Savannasaurus* [40].

### 7.2. Sacral vertebrae

In vertebrate palaeontology, a vertebra is interpreted as a sacral if it makes contact with the ilium [216,217]. However, sacral centra also coalesce through ontogeny, such that the vertebrae that contact the ilium also tend to become coossified in adults [95]. Thus, we contend that it is reasonable to interpret coalesced, non-pathological vertebrae in sauropods as sacral, particularly when they are morphologically consistent with other described sacral vertebrae.

**Figure 22.**
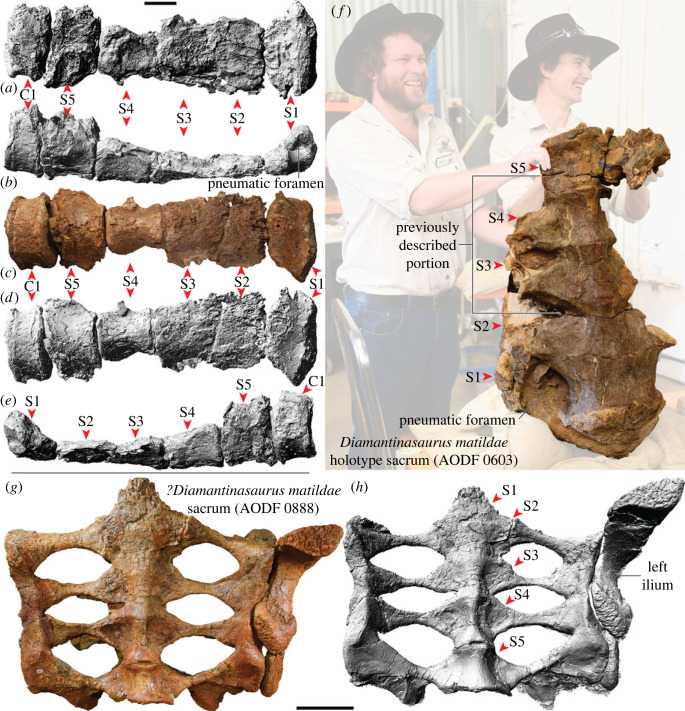
*Diamantinasaurus matildae* referred sacrum and caudal vertebra 1 (AODF 0906), *Diamantinasaurus matildae* holotype sacrum (AODF 0603); and ?*Diamantinasaurus matildae* referred sacrum and left ilium (AODF 0888). (*a–e*) *Diamantinasaurus matildae* referred (AODF 0906) sacral vertebrae 1–5 and caudal vertebra 1 of AODF 0906 in (*a*) dorsal, (*b*) right lateral, (*c*,*d*) ventral and (*e*) left lateral views. (*a–b*) and (*d–e*) are three-dimensional models derived from surface scans, (*c*) is a photograph. Scale bar = 100 mm. (*f*) *Diamantinasaurus matildae* holotype (AODF 0603) sacrum in ventrolateral view, with anterior towards bottom of page, showing the position of the previously described portion indicated. (*g*,*h*) ?*Diamantinasaurus matildae* referred (AODF 0888) sacrum and left ilium in ventral view. (*g*) is a photograph, (*h*) is a three-dimensional model derived from a surface scan. Scale bar = 200 mm.

The sacrum of AODF 0906 (figure 22*a–e*) was found in three pieces: one comprising the centrum and partial lower transverse processes of sacral vertebra I, a second comprising sacral centra II–IV (and the bases of the lower transverse processes of sacral vertebra IV), and a third comprising the centrum and partial lower transverse processes of sacral vertebra V.

The sacrum of AODF 0906 comprises five sacral vertebrae, albeit with the centrum of sacral vertebra V unsutured to sacral vertebra IV. Although some non-titanosaurian somphospondylans, namely *Huanghetitan liujiaxiaensis* [218], *Sauroposeidon* [175], *Sibirotitan* [219] and *Tastavinsaurus* [220,221], also have five sacral vertebrae, most somphospondylans—including all known titanosaurs that preserve complete sacra, as well as several taxa that probably lie outside of Titanosauria (e.g. *Dongyangosaurus*, *Huabeisaurus*, *Phuwiangosaurus* and *Ruyangosaurus* [149,222–224])—have six or more [95,100,101,106,109]. Thus, AODF 0906 appears to be plesiomorphic with respect to this character. The sacrum of the holotype specimen of *Diamantinasaurus matildae* (AODF 0603), which has been more completely prepared since its initial description [37], is now known to also comprise only five coalesced vertebrae (figure 22*f*). The previously figured portion of the sacrum of AODF 0603 comprises sacral vertebrae II–V, not III–VI as labelled in Poropat *et al*. [37]). An as yet unpublished diamantinasaurian specimen from the Winton Formation (AODF 0888), which is also likely referable to *Diamantinasaurus matildae*, preserves a sacrum comprising five coossified vertebrae: the transverse processes of the posterior four sacral vertebrae unequivocally form a sacricostal yoke that contacts the ilium (figure 22*g,h*). We infer that the vertebra fused to the anteriormost of these four also contacted the ilium, giving a sacral count of five. Whether or not an additional sacral vertebra was present anterior to this is unclear, but we consider it unlikely.

In AODF 0906, the anterior articular surface of the centrum of sacral vertebra I is convex and dorsoventrally compressed. Each lateral surface of sacral vertebra I preserves a pneumatic foramen, with that on the right side (figure 22*b*) better preserved than that on the left (figure 22*e*). The holotype sacrum of *Diamantinasaurus matildae* (AODF 0603) also has a prominent pneumatic foramen on sacral vertebra I (figure 22*f*). However, in both AODF 0603 and AODF 0906, pneumatic foramina are not evident on any of the other sacral centra. The presence of a pneumatic foramen on at least sacral vertebra I in both AODF 0603 and AODF 0906 distinguishes *Diamantinasaurus* from some titanosaurs, including *Opisthocoelicaudia skarzynskii* and *Saltasaurus*, wherein all sacral vertebrae lack pneumatic foramina [100,225]. Sacral vertebrae II and III in AODF 0906 have suffered extensive taphonomic dorsoventral compression (possibly a result of *post mortem* trampling by another dinosaur) and are too poorly preserved to elicit much comment. Their internal texture is camellate (possibly semicamellate), as in somphospondylans generally [101], and the transverse widths of the middle sacral centra appear to be little different from those of the first and last sacral centra, distinguishing AODF 0906 from saltasaurids and aeolosaurines, in which the middle centra are ‘waisted' relative to the first and last sacral centra [38,226–228]. The ventral surfaces of sacral vertebrae IV and V are anteroposteriorly concave and transversely convex. The acuteness of the transverse convexity is greater in sacral IV than in sacral V, such that it almost forms a weak median ridge in the former (figure 22*d*). Sacral vertebrae IV and V each preserve the bases of their transverse processes, and these are situated near the mid-length of the centrum in both vertebrae. The posterior articular surface of sacral vertebra IV and the anterior articular surface of the centrum of sacral vertebra V are both essentially flat, and these vertebrae were clearly unsutured in life; this implies that AODF 0906 was osteologically immature at death. The posterior articular surface of sacral vertebra V is shallowly concave and slightly wider transversely than tall dorsoventrally.

### 7.3. Caudal vertebra

The only caudal vertebra preserved is represented by an incomplete centrum (figure 22*a–e*). It is presumably caudal vertebra I, based on the size congruence of its anterior articular surface with that of the posterior articular surface of sacral vertebra V. Breakages on the dorsal surface of the centrum either side of the ventral margin of the neural canal reveal spongiose, but not camellate, internal texture. In this regard, it is similar to most sauropods, in which camellae do not extend into the tail [104,229]; however, it is possible that the AODF 0906 caudal neural arch (which is not preserved) was characterized by camellae, given that this tissue structure is present in that region, but not the centrum, in anterior caudal vertebrae of *Savannasaurus* [40]. The articular surfaces of the centrum are wider transversely than they are tall dorsoventrally (table 4), as in many titanosauriforms [8,101]. Both articular surfaces are shallowly concave, as is the case in the anteriormost caudal centra of some brachiosaurids and non-titanosaurian somphospondylans (wherein they are flat or concave), and differs from nearly all other titanosaurs, wherein one or both articular surfaces are convex [6,38,96,100,101,104,106,109,131]. However, *Savannasaurus* is also characterized by amphicoelous anterior caudal centra [38,40]. The average Elongation Index of the centrum of AODF 0906, calculated by dividing the anteroposterior length of the centrum by the mean of the mediolateral width and dorsoventral height of the anterior articular surface of the centrum [6,8,100,101,106,132], is 0.63. The ventral surface is anteroposteriorly concave and transversely convex (figure 22*e*). A tiny foramen (possibly pneumatic) is evident on the right lateral surface (figure 22*b*), and several probable nutrient foramina are present on the less complete left lateral surface (figure 22*e*). Despite the nearly complete state of the centrum, there is no evidence of a transverse process or a pneumatic foramen; this distinguishes AODF 0906 from the proximal–middle anterior caudal vertebrae of *Savannasaurus* [40]. However, a pneumatic opening is not always present in the anteriormost caudal centra, with substantial serial variation observed in some taxa [230,231], and thus its absence in AODF 0906 might merely reflect its proximal placement in the tail.

**Table 4.** Measurements of the sacral vertebrae and caudal vertebra of AODF 0906 *Diamantinasaurus matildae*. An asterisk (*) indicates a tentative measurement based on an incomplete or distorted specimen.

measurements (mm)			sacral vertebrae					caudal vertebra
			I	II	III	IV	V	
centrum	anteroposterior length		118	138	139	147	134	116
	anterior	maximum dorsoventral height	124	—	—	—	155	165
		maximum transverse width	247	194*	145*	126	144	201
	midline	minimum transverse width	192	137*	127*	92	149	—
	posterior	maximum dorsoventral height	—	—	—	112	168	162
		maximum transverse width	—	218*	154	135	214	205

### 7.4. Chevron

**Figure 23.**
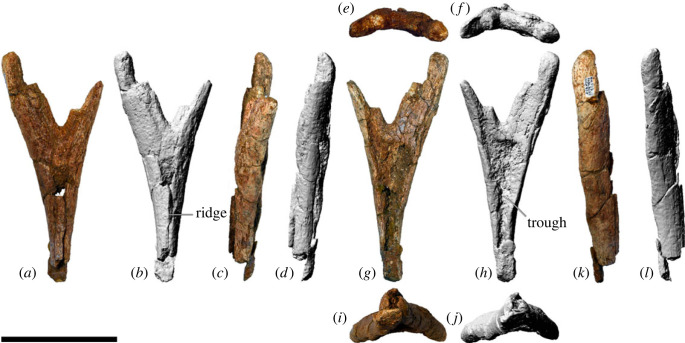
*Diamantinasaurus matildae* referred chevron (AODF 0906). (*a–l*) Chevron in (*a*,*b*) anterior, (*c*,*d*) left lateral, (*e*,*f*) dorsal, (*g*,*h*) posterior, (*i*,*j*) ventral and (*k*,*l*) right lateral views. (*a*), (*c*), (*e*), (*g*), (*i*) and (*k*) are photographs, and (*b*), (*d*), (*f*), (*h*), (*j*) and (*l*) are three-dimensional models derived from surface scans. Scale bar = 100 mm.

The sole chevron preserved is almost complete (figure 23; table 5), missing only the distal end, the proximal left ramus, and the medial half of the right ramus. The chevron has also been slightly impacted by *post mortem* distortion. As is typical of titanosauriform chevrons, it is Y-shaped in anterior (figure 23*a,b*) and posterior (figure 23*g,h*) aspects. Similarly, as in most titanosauriforms (other than, for example, *Dongbeititan dongi*, *Daxiatitan binglingi* and *Xianshanosaurus shijiagouensis*), the haemal canal is open proximally rather than bridged [100,101,106,144,232]. The depth of the haemal canal is slightly less than one third the total proximodistal length of the chevron; the chevrons of the contemporaneous titanosauriform *Wintonotitan* show a similarly shallow haemal canal [36], as do those of some other titanosauriforms, including the non-titanosaurian somphospondylans *Dongbeititan*, *Daxiatitan*, *Xianshanosaurus* and *Sauroposeidon*, and the saltasaurine titanosaur *Saltasaurus* [101]. As preserved, the proximal articular surface of the right ramus is rounded and undivided (figure 23*e,f*); if this reflects the true morphology of the proximal articular surface, then AODF 0906 lacks the grooves on this surface seen in some somphospondylans, including the early-branching members *Tangvayosaurus hoffeti* and *Phuwiangosaurus* [5], and several titanosaurs, such as *Epachthosaurus sciuttoi*, *Lohuecotitan pandafilandi*, *Mendozasaurus neguyelap*, *Notocolossus gonzalezparejasi* and *Aeolosaurus* [5,38,145,233–236]. The anterior surface of each ramus, and the anterior surface immediately distal to their junction, is smoothly transversely convex. The lateral surface of each ramus lacks ridges and bulges (figure 23*c,d*,*k,l*), thereby contrasting with the chevrons of *Epachthosaurus* and some saltasaurids, in which such structures are present [38,234], although these are sometimes absent from the anteriormost chevrons [2]. The distal blade forms a prominent ridge anteriorly and hosts a deep trough posteriorly. This contrasts with *Wintonotitan*, wherein both the anterior and posterior margins of the distal blade form ridges [36].

**Table 5.** Measurements of the chevron of AODF 0906 *Diamantinasaurus matildae*. An asterisk (*) indicates a tentative measurement based on an incomplete or distorted specimen.

measurements (mm)		chevron
dorsoventral height		201*
haemal canal depth		62*
haemal canal depth: chevron dorsoventral height		∼0.31
distal shaft	anteroposterior	23
	transverse	21.5

## 8. Pelvic girdle

### 8.1. Ilium

**Figure 24.**
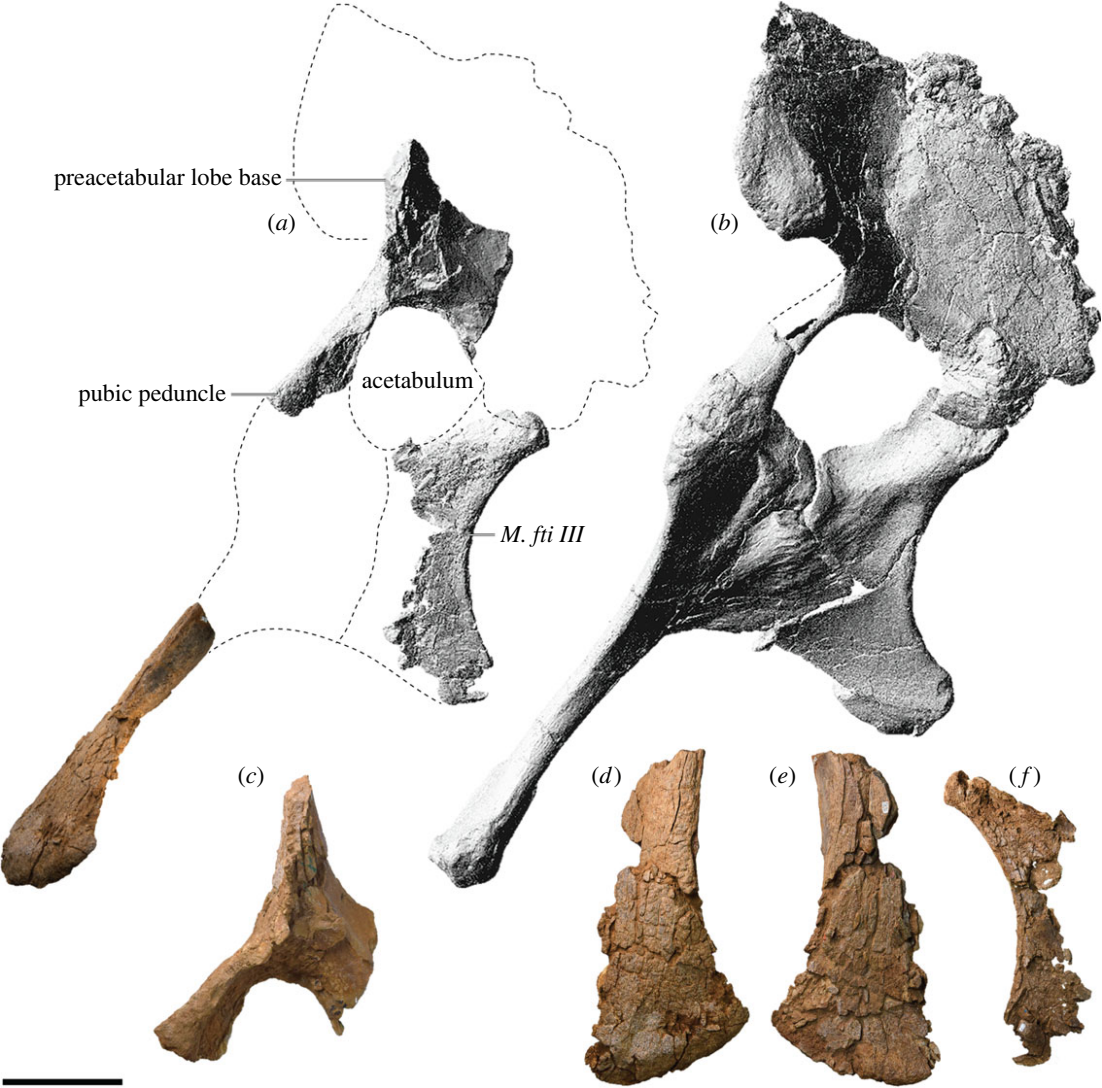
*Diamantinasaurus matildae* referred pelvis (AODF 0906), and *Diamantinasaurus matildae* holotype pelvis (AODF 0603). (*a*) *Diamantinasaurus matildae* referred (AODF 0906) left ilium, pubis and ischium in lateral view. (*b*) *Diamantinasaurus matildae* holotype (AODF 0603) articulated left ilium, pubis and ischium. (*c*) *Diamantinasaurus matildae* referred (AODF 0906) left ilium in lateral view. (*d*,*e*) *Diamantinasaurus matildae* referred (AODF 0906) left pubis in (*d*) anterior and (*e*) posterior views. (*f*) *Diamantinasaurus matildae* referred (AODF 0906) left ischium in medial view. (*a*) and (*b*) are three-dimensional models derived from surface scans (excluding the pubis in (*a*)), and (*c–f*) are photographs. *M. fti III*, *M. flexor tibialis internus III*. Scale bar = 200 mm.

The incomplete left ilium preserves the pubic and ischiadic processes—and therefore most of the acetabular margin—but lacks much of the blade, including the pre- and postacetabular processes (figure 24*a,c* and table 6). In all respects, it is practically identical to the ilium of the holotype of *Diamantinasaurus matildae* (figure 24*b*; [37]); it is also somewhat akin to the similarly incomplete ilium of *Wintonotitan wattsi* [36]. Despite being incompletely preserved, it is clear that, when viewed dorsally, the preacetabular lobe would have flared anterolaterally, as in neosauropods generally [100,106,109,144]. The pubic peduncle is perpendicular to the long axis of the AODF 0906 ilium, as in virtually all titanosauriforms [96]. The anteroposterior length (61 mm) of the incomplete pubic peduncle of the ilium is barely more than 25% its mediolateral width (220 mm), compared with just under 50% in the holotype of *Diamantinasaurus matildae* [37]. In saltasaurids, the anteroposterior length of the pubic peduncle of the ilium is at least 50% its mediolateral width [6].

**Table 6.** Measurements of the left ilium and ischium of AODF 0906 *Diamantinasaurus matildae*. An asterisk (*) indicates a tentative measurement based on an incomplete or distorted specimen.

measurement (mm)	left ilium	left ischium
maximum proximodistal length	—	646
maximum anteroposterior length	422*	242
maximum mediolateral diameter of pubic peduncle	228*	—
maximum anteroposterior diameter of pubic peduncle	61*	—
maximum mediolateral diameter of iliac peduncle	—	63*
maximum anteroposterior diameter of iliac peduncle	—	77*

### 8.2. Pubis

**Figure 25.**
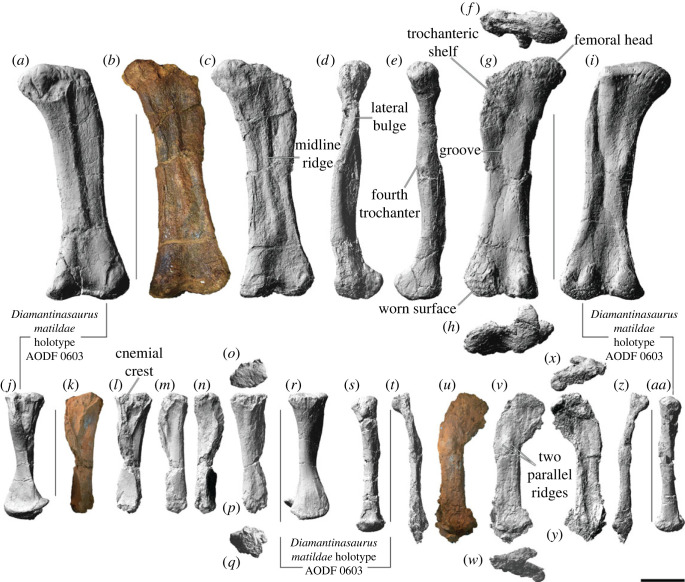
*Diamantinasaurus matildae* holotype hind limb elements (AODF 0603), and *Diamantinasaurus matildae* referred hind limb elements (AODF 0906). (*a* and *i*) *Diamantinasaurus matildae* holotype (AODF 0603) left femur (right femur mirrored) in (*a*) anterior and (*i*) posterior views. (*b–h*) *Diamantinasaurus matildae* referred (AODF 0906) left femur in (*b–c*) anterior, (*d*) lateral, (*e*) medial, (*f*) proximal, (*g*) posterior and (*h*) distal views. (*j*,*r*) *Diamantinasaurus matildae* holotype (AODF 0603) left tibia (right tibia mirrored) in (*j*) anterior and (*r*) posterior views. (*k–q*) *Diamantinasaurus matildae* referred (AODF 0906) left tibia in (*k–l*) anterior, (*m*) lateral, (*n*) medial, (*o*) proximal, (*p*) posterior and (*q*) distal views. (*s* and *aa*) *Diamantinasaurus matildae* holotype (AODF 0603) left fibula (right fibula mirrored) in (*s*) anterior and (*aa*) posterior views. (*t–z*) *Diamantinasaurus matildae* referred (AODF 0906) left fibula in (*t*) anterior, (*u–v*) lateral, (*w*) distal, (*x*) proximal, (*y*) medial and (*z*) posterior views. (*a*), (*c–j*), (*l–t*) and (*v*–*aa*) are three-dimensional models derived from surface scans, and (*b*), (*k*) and (*u*) are photographs. Scale bar = 250 mm.

The left pubis (figure 25*a* and *d,e*) is represented by its distal one-half and is practically identical to the pubes of the holotype of *Diamantinasaurus matildae* [37]. It is also very similar to the pubes of *Australotitan cooperensis* [34]. The distal end is laminar and expanded to form an anterior boot, as in some other titanosaurs [3,38,101], albeit not to the extent seen in *Savannasaurus elliottorum* [40].

### 8.3. Ischium

The left ischium is more complete than the right (figure 24*a* and *f*; table 6), although it is missing almost the entire pubic articular surface, a substantial portion of its iliac peduncle, and most (if not all) of the ischiadic articular surface. In all respects it is very similar to the holotype ischia of *Diamantinasaurus matildae* (figure 24*b*; [37]), *Savannasaurus elliottorum* [38,40] and *Australotitan cooperensis* [34]. It is also very similar to the holotype left ischium (QM F7292) of *Wintonotitan wattsi*, although the lateral ridge for *M. flexor tibialis internus III* is not as pronounced in AODF 0906 [36]. The preserved portion of the iliac peduncle demonstrates that it does not narrow anteroposteriorly in lateral view, and the mediolateral width of the acetabular margin is effectively uniform along its length. The only measurable portion of the proximal plate has an anteroposterior length of 177 mm, slightly greater than 25% the total proximodistal length of the ischium. This distinguishes AODF 0906 from several non-titanosaurian titanosauriforms, wherein the anteroposterior length of the proximal plate comprises less than 25% of the proximodistal length of the ischium [6,101]. The long axis of the shaft of the ischium projects towards the lower acetabular margin, as in all somphospondylans [95,100,101,106]. The attachment point for *M. flexor tibialis internus III* is a low ridge. No groove accompanies this ridge, with this also absent in most other titanosauriforms [5,6,38].

## 9. Hind limb

### 9.1. Femur

The right femur is incomplete and poorly preserved in two sections. By contrast, the left femur (figure 25*b–h*; table 7) is well preserved and almost complete, missing only the greater trochanter, part of the fibular condyle, and the lateral epicondyle (assuming it was present). It has, however, suffered some distortion, especially on the lateral margin (the curvature seen in figure 25*d* is not considered to be natural). The subsequent description is based entirely on the left femur.

**Table 7.** Measurements of the left femur of AODF 0906 *Diamantinasaurus matildae*.

measurements (mm)	left femur
proximodistal length	1366
proximal end mediolateral width	430
mid-shaft mediolateral width	261
mid-shaft anteroposterior length	126
mid-shaft minimum circumference	686
distal end maximum mediolateral width	435

In all measured dimensions, the left femur of AODF 0906 (table 7) is very slightly larger than the holotype right femur of *Diamantinasaurus matildae* (AODF 0603; [9,37,44,237]). However, the measured proximodistal length is likely exaggerated (possibly by as much as 50 mm) by infilled fractures in the element. Morphologically, the femur is also closely comparable with the holotype femur of *Diamantinasaurus matildae* [9,37]. The only notable difference between the femora of AODF 0906 and the *Diamantinasaurus matildae* holotype specimen (AODF 0603: figure 25*a,i*) is the morphology of the lateral bulge: in the latter, this structure is separated from the rest of the femur by a deep, longitudinal groove [37]. However, in AODF 0906 this section of the femur is flat. The AODF 0906 left femur shows evidence of substantial distortion, thereby accounting for the slight morphological disparity between it and the *Diamantinasaurus matildae* holotype femur observed here.

The femoral head is directed medially, rather than dorsomedially (figure 25*b,c,g*); among titanosaurs, medially deflected femoral heads are present in relatively few taxa, including *Alamosaurus*, *Diamantinasaurus*, *Jainosaurus*, *Malawisaurus*, *Mendozasaurus* and *Pitekunsaurus* [3,8,38,238]. The proximal lateral margin forms a flange-like trochanteric shelf, as in nearly all titanosaurs [101]. The proximolateral margin (dorsal to the lateral bulge) lies medial to the lateral margin of the distal half of the femoral shaft, differentiating AODF 0906 from some early-branching somphospondylans (e.g. *Tastavinsaurus*), *Patagotitan mayorum*, and saltasaurid titanosaurs, wherein this margin lies lateral to the lateral margin of the distal half of the femoral shaft [96,101,109,221,232,238,239]. The anterior surface (figure 25*b,c*) preserves a longitudinal midline ridge (*linea intermuscularis cranialis*) and a concavity on its proximal two-thirds, as is also the case in the *Diamantinasaurus matildae* holotype (figure 25*a*; [37]), as well as several other titanosaurs, including *Alamosaurus*, *Australotitan*, *Pellegrinisaurus powelli* and saltasaurines [5,34,240,241]. The fourth trochanter, which is situated on the posterior surface, slightly proximal to the mid-length (figure 25*g*), is manifested as a low, rounded ridge, contrasting with saltasaurines, wherein it is reduced to near absence [2]. The fourth trochanter is not visible in anterior view, distinguishing AODF 0906 from most non-somphospondylan macronarians, as well as some non-titanosaurian (e.g. *Phuwiangosaurus*, *Sauroposeidon*) and titanosaurian (e.g. *Malarguesaurus*, *Tapuiasaurus*) somphospondylans [101]. The femoral shaft is anteroposteriorly compressed (ratio of mediolateral width: anteroposterior length at midshaft = 2.07 versus 2.18 in the holotype; figure 25*d,e*) and the greatest anteroposterior thickness of the shaft (93.5 mm) is slightly more than one-third that of the anteroposterior length of the tibial condyle (272.5 mm); by contrast, in some non-titanosaurian macronarians, the anteroposterior thickness of the shaft is at least half the anteroposterior length of the tibial condyle [131]. A longitudinal groove extends along the mid-shaft of the posterior surface (figure 25*g*). The distal condyles are similar to one another in mediolateral width (figure 25*h*) and are bevelled dorsomedially such that the fibular condyle extends further distally than the tibial one, as in many titanosaurs [101,104,242], including the *Diamantinasaurus matildae* holotype [37]. The distal condyles do not extend on to the anterior surface of the shaft, contrasting with the condition in titanosaurs such as *Rapetosaurus* and saltasaurines [104,242].

### 9.2. Tibia

The right tibia is incomplete, missing much of its proximal end. However, its distal end is reasonably well preserved and undistorted. The left tibia is similarly fragmentary (figure 25*k–q*; table 8), but does preserve the proximal end (which is incomplete on all margins except the anterior one), despite lacking the distal one. The following description is largely based on the left tibia, with supplementary information derived from the right one.

**Table 8.** Measurements of the left tibia of AODF 0906 *Diamantinasaurus matildae*. An asterisk (*) indicates a tentative measurement based on an incomplete or distorted specimen.

measurements (mm)	left tibia	right tibia
proximodistal length	786*	532*
proximal end mediolateral width	152*	—
proximal end anteroposterior width	247*	—
mid-shaft long axis diameter	135*	—
mid-shaft dimension perpendicular to long axis	98*	—
mid-shaft minimum circumference	457	—
distal end maximum mediolateral width	>155*	241
distal end maximum anteroposterior width	132*	170

The tibia of AODF 0906 is very similar to that of the *Diamantinasaurus matildae* holotype specimen (figure 25*j* and *r*; [37]). The proximodistal length of the composite tibia is just under 60% the length of the femur. This is comparable to the condition in the holotype of *Diamantinasaurus matildae* [37], as well as in several non-titanosaurian somphospondylans [38], including *Chubutisaurus*, *Ligabuesaurus* and *Tastavinsaurus* [174,220,243]. The lateral edge of the proximal end (posterior to the cnemial crest) lacks the pinched-out projection, or ‘second cnemial crest', seen in some somphospondylans [101]. The deep anterolateral fossa hosts a small proximal projection and is bordered posteriorly by a sharp longitudinal ridge (the presence of a double ridge cannot be determined owing to incompleteness). Several tibial autapomorphies of *Diamantinasaurus matildae* [37] cannot be assessed in AODF 0906, including whether or not a fossa was present posterior to the lateral longitudinal ridge(s). No tubercle is evident on the posterior/medial surface of the cnemial crest, contrasting with some early-diverging macronarians [157]. The complete and undistorted distal end of the right tibia is, in all respects, effectively identical to the holotype tibia of *Diamantinasaurus matildae* [37].

### 9.3. Fibula

The left fibula is almost complete (figure 25*t–z* and table 9), but has suffered substantial mediolateral distortion, particularly at its proximal and distal ends. Consequently, it is not possible to assess the presence or absence of the sole fibular autapomorphy of *Diamantinasaurus matildae*: the ridges and grooves on the medial surface near the mid-length [37]. Nevertheless, the fibula of AODF 0906 is similar to that of the *Diamantinasaurus matildae* holotype specimen (figure 25*s* and AA). The proximal end is comma-shaped, with an anteromedially directed crest, as in many somphospondylans [5,101,113]. A medial triangular scar appears to be present at the proximal end. The fairly prominent lateral trochanter, which is manifested as two parallel ridges, as in most titanosauriforms [100,144], is situated one-third of the way down the fibula shaft. In lateral view, the shaft appears sigmoidal, but this is only because of the substantial mediolateral (and oblique) taphonomic distortion to which this element has been subjected: it was likely straight in life. The right fibula is less complete, but better preserved, than the left fibula. The proximal end is missing, but the mid-shaft and distal end are well represented; the distal end is practically identical to the subtriangular distal end of the holotype fibula (AODF 0603) of *Diamantinasaurus matildae* [37].

**Table 9.** Measurements of the left fibula of AODF 0906 *Diamantinasaurus matildae*. An asterisk (*) indicates a tentative measurement based on an incomplete or distorted specimen.

measurements (mm)	left fibula	right fibula
proximodistal length	865	704*
maximum proximal mediolateral breadth	79	—
maximum proximal anteroposterior width	223	—
mid-shaft mediolateral breadth	63	—
mid-shaft anteroposterior width	134	121*
mid-shaft minimum circumference	357	—
maximum distal mediolateral breadth	93*	98
maximum distal anteroposterior width	>200*	136

### 9.4. Astragalus

A worn element might represent that the worn dorsal portion of the right astragalus is present. However, very little of its morphology can be appraised.

### 9.5. Metatarsals

**Figure 26.**
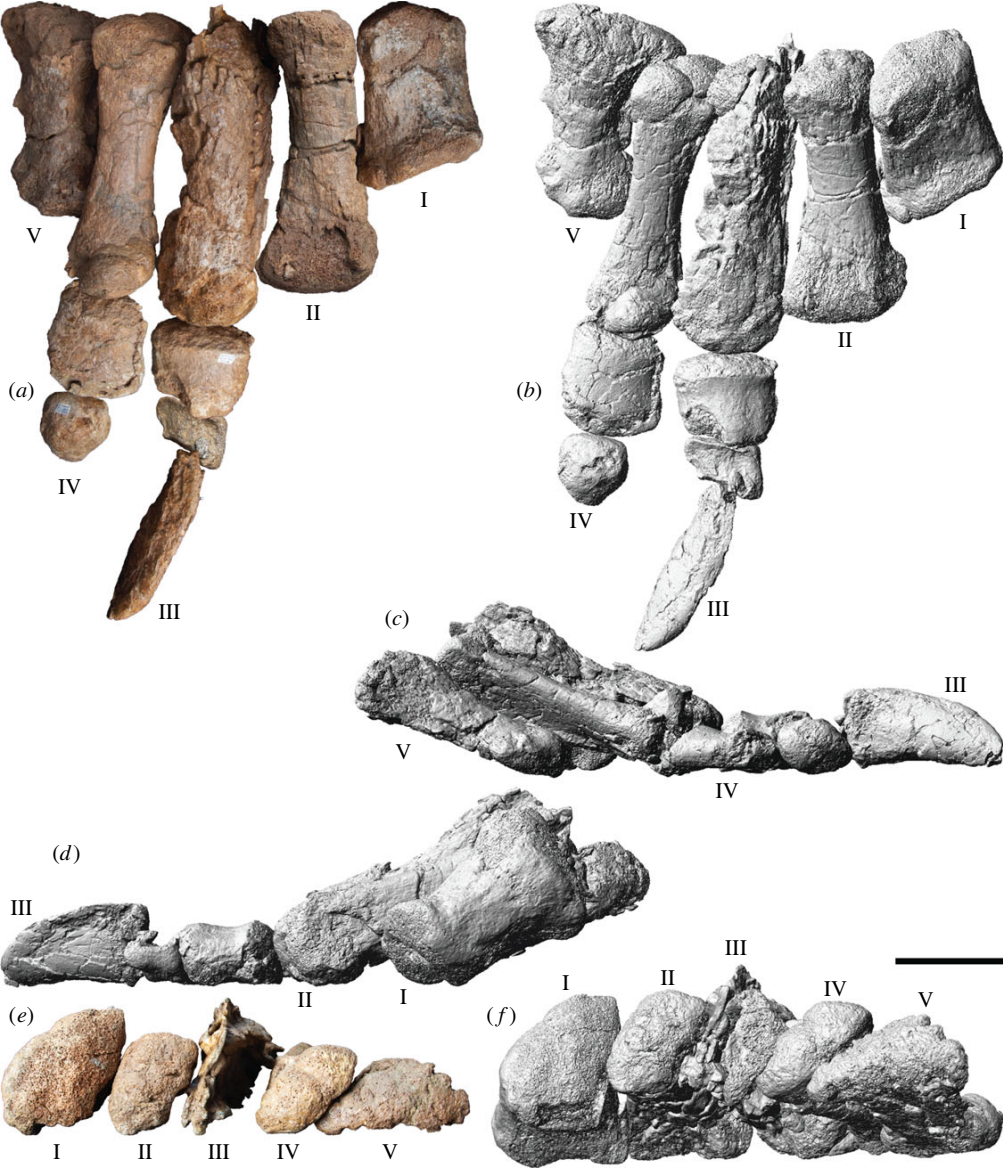
*Diamantinasaurus matildae* referred right pes (AODF 0906). (*a–f*) Articulated right pes in (*a*,*b*) dorsal, (*c*) lateral, (*d*) medial and (*e*,*f*) proximal views. (*a*) and (*e*) are photographs; (*b*–*d*) and (*f*) are three-dimensional models derived from surface scans. Scale = 100 mm.

**Figure 27.**
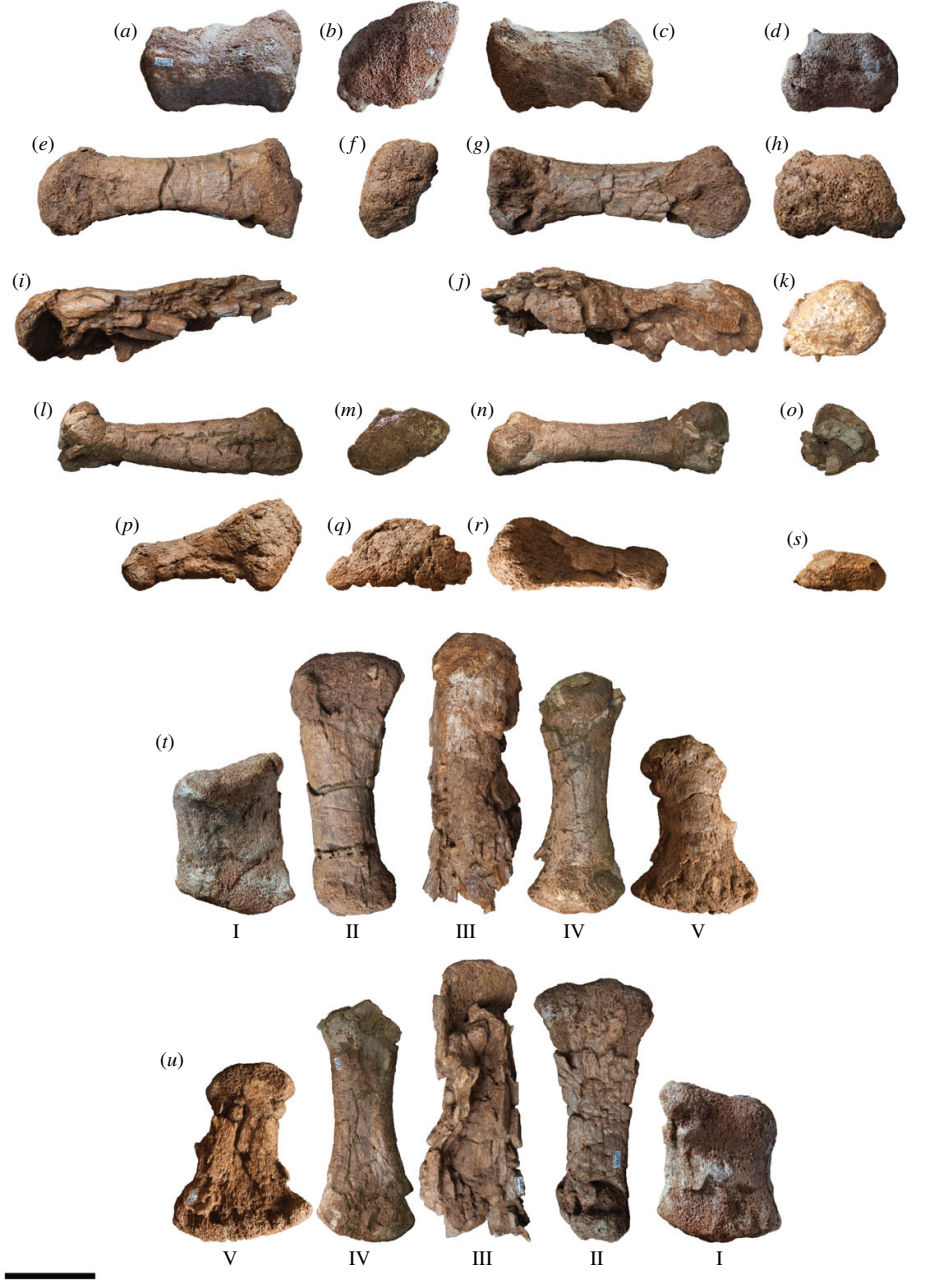
*Diamantinasaurus matildae* referred right metatarsals (AODF 0906). (*a–d*) Right metatarsal I in (*a*) medial, (*b*) proximal, (*c*) lateral and (*d*) distal views. (*e–h*) Right metatarsal II in (*e*) medial, (*f*) proximal, (*g*) lateral and (*h*) distal views. (*i–k*) Right metatarsal III in (*i*) medial, (*j*) lateral and (*k*) distal views. (*l–o*) Right metatarsal IV in (*l*) medial, (*m*) proximal, (*n*) lateral and (*o*) distal views. (*p–s*) Right metatarsal V in (*p*) medial, (*q*) proximal, (*r*) lateral and (*s*) distal views. (*t–u*) Right metatarsals I–V in (*t*) dorsal and (*u*) ventral (plantar) views. Scale bar = 100 mm.

AODF 0906 preserves all five right metatarsals (figures 26 and 27; table 10). Metatarsal III is the longest, followed by IV, II, V and I. The fact that metatarsal I is shorter proximodistally than metatarsal V (ratio 0.8) is a feature that AODF 0906 shares with most macronarians other than *Opisthocoelicaudia* and *Rapetosaurus* [101]. The substantial disparity between the proximodistal lengths of metatarsal III and I (III : I ratio = 1.76, although taphonomic distortion might have slightly exaggerated the length of metatarsal III) is a feature that AODF 0906 shares with most titanosauriforms, other than *Notocolossus* wherein this ratio is 1.2 [236,244]. Metatarsal III is longer proximodistally than metatarsal IV (III : IV ratio = 1.09), as in most titanosauriforms (e.g. *Opisthocoelicaudia*, *Rapetosaurus*, *Tastavinsaurus*) other than *Alamosaurus*, *Epachthosaurus* and *Notocolossus* [236,244]. Metatarsals I and II appear to lack dorsolateral rugosities near their distal ends, distinguishing AODF 0906 from some diplodocoids [106].

**Table 10.** Measurements of the right metatarsals of AODF 0906 *Diamantinasaurus matildae*. An asterisk (*) indicates a tentative measurement based on an incomplete or distorted specimen.

measurement (mm)	right metatarsals				
	I	II	III	IV	V
proximodistal length	157	243	277*	255*	198
maximum proximal mediolateral width	85	82	—	98	135
maximum proximal dorsoventral height	109	91	—	73	65
mid-shaft mediolateral breadth	98	64	—	46	70*
maximum distal mediolateral width	110	110	96*	84	94
maximum distal dorsoventral height	74	77	65*	50*	40

#### 9.5.1. Metatarsal I

Right metatarsal I (figure 27*a–d,t,u*) is almost complete, missing only a small section of the ventrolateral corner of the proximal end. In proximal view (figure 27*b*), the metatarsal has a convex medial margin and a lateral margin that is flat in its dorsal half and concave in its ventral half. The dorsal junction between the medial and lateral margins is manifested as a point situated near the lateral margin. When complete, the incomplete ventral margin was presumably broadly convex. The proximal surface is convex. In dorsal view (figure 27*t*), the distal half of metatarsal I shows effectively no flaring: it shares this morphology with some early-branching somphospondylans (e.g. *Tastavinsaurus*) and titanosaurs (e.g. *Epachthosaurus*, *Mendozasaurus*, *Opisthocoelicaudia*), but differs from other early-branching somphospondylans (e.g. *Gobititan*, *Ligabuesaurus*) and titanosaurs (e.g. *Alamosaurus*, *Muyelensaurus*, *Notocolossus*, *Rapetosaurus*, *Saltasaurus*), wherein ventrolateral expansion of this element results in significant distal flaring [101,245]. The dorsal surface is concave proximodistally, convex mediolaterally, and inclined such that it faces somewhat dorsomedially. The junction between the dorsal and lateral surfaces is a reasonably pronounced ridge. The lateral surface is essentially flat, faces slightly ventrolaterally, and is flared proximally and tapered distally (figure 27*c*). The ventral surface is incomplete proximolaterally but was evidently concave centrally and convex along its proximomedial and distal margins (figure 27*u*). The medial margin merges smoothly with both the dorsal and ventral margins (figure 27*a*). Although it has a subtle proximodistally elongate ridge at its mid-height, it appears to lack the median tubercle on this surface that is present in several brachiosaurids [6,246]. The distal end is ‘D'-shaped, with the slightly concave ventral margin corresponding to the flat side of the ‘D' and all other margins convex (figure 27*d*). The distal surface itself is mediolaterally concave and dorsoventrally convex either side of the midline, essentially forming two condyles for articulation with pedal phalanx I-1.

#### 9.5.2. Metatarsal II

Right metatarsal II is practically complete (figure 27*e–h,t,u*), with only minor wear evident at the proximal and distal ends. The proximodistal length of metatarsal II is 30% that of the incomplete tibia. In proximal view (figure 27*f*), the metatarsal is broadly wedge-shaped. The dorsal margin is narrow and mediolaterally convex, and the medial margin is shallowly convex. The lateral margin is somewhat more complex, being divisible into a shallowly concave section that faces laterally (dorsal two-thirds) and a straight section that faces ventrolaterally (ventral one-third). In several other titanosaurs (e.g. *Epachthosaurus*, *Mendozasaurus*, *Muyelensaurus*), the proximal end of metatarsal II is also concave laterally; however, in most taxa it is straight [101]. The proximal surface is mostly concave, but hosts a subtle depression in its dorsal one-third. In dorsal view, metatarsal II is flared at both the proximal and distal ends, with the flaring of the latter significantly more pronounced than the former. Although the distal one-quarter appears to have hosted a slight depression, this surface is otherwise proximodistally concave and almost entirely mediolaterally convex (figure 27*t*). The junction between the dorsal and lateral surfaces is manifested as a very subtle ridge that is more pronounced distally. The lateral surface (figure 27*g*) is proximodistally concave, dorsoventrally convex, and twisted such that the proximal one-third faces slightly ventrolaterally to accommodate metatarsal III. Separated from the lateral surface by a subtle ridge, the ventral surface (figure 27*u*) varies in aspect along its length despite being broadly proximodistally concave: whereas the proximal half is mediolaterally convex and faces slightly laterally, the distal half faces entirely ventrally and is mediolaterally concave. The medial surface is essentially flat (figure 27*e*), and its junctions with both the dorsal and ventral surfaces are not marked by ridges despite being relatively abrupt. The ventral surface is convex (figure 27*u*). In distal view (figure 27*h*), the metatarsal is essentially trapezoidal, with the ventral margin mediolaterally wider than the dorsal one and the lateral and medial margins inclined inwards towards the dorsal margin. In AODF 0906, and in most somphospondylans, the distal articular surface does not extend on to the dorsal margin of the shaft; however, in some somphospondylans (e.g. *Alamosaurus*, *Epachthosaurus*, *Euhelopus*, *Gobititan*), the inverse is true [101,245]. The ventral margin of the distal end is concave, lacking the ventrolateral projection seen in the early-branching somphospondylans *Dongbeititan*, *Gobititan* and *Tastavinsaurus*, as well as the titanosaur *Muyelensaurus* [6].

#### 9.5.3. Metatarsal III

Metatarsal III (figure 27*i–k,t,u*) is incomplete proximally and ventrally and has suffered substantial taphonomic distortion (dorsoventral compression). Consequently, its preserved proximodistal length might be a slight exaggeration. Nevertheless, it is probable that metatarsal III was at least 25% (and likely more than 30%) the length of the tibia. Few titanosaurs can be appraised for this feature, but those that can (e.g. *Epachthosaurus*, *Opisthocoelicaudia*) also have third metatarsals that are elongate relative to tibia length; among early-deriving somphospondylans, only *Tastavinsaurus* shares this feature [101,220]. Despite the distortion to which it has been subjected, it appears that the metatarsal III of AODF 0906 was less robust than that of *Savannasaurus elliottorum* [38,40].

#### 9.5.4. Metatarsal IV

Right metatarsal IV is practically complete (figure 27*l–o,t,u*), but the distal end is fractured and slightly distorted. The proximal end is sub-rhomboidal (figure 27*m*), with essentially straight dorsomedial and dorsal margins that meet at a point, and a broadly convex ventrolateral margin which is kinked slightly dorsomedially at two-thirds its overall length. The proximal surface is almost entirely convex, with the exception of a shallow depression extending from the dorsomedial junction to the ‘kink' on the ventrolateral margin; it is not clear if this corresponds to the embayment present in metatarsal IV in some titanosauriforms [5]. In dorsal view (figure 27*t*), both the proximal and distal ends are flared relative to the shaft, with the former more so than the latter as preserved. Both the dorsal and medial surfaces of the shaft are smoothly and shallowly convex mediolaterally along their respective lengths, with their junction only perceptible because it is slightly more strongly convex than either surface. Thus, the medial surface lacks the proximal concavity to accommodate metatarsal IV seen in some somphospondylans, including the early-branching forms *Chubutisaurus*, *Ligabuesaurus* and *Tastavinsaurus*, and the titanosaurs *Alamosaurus* and *Notocolossus* [5,174,243–245]. In dorsal view, both the medial and lateral margins are proximodistally concave, with the medial margin more strongly so than the lateral one. Along its length, the ventrolateral surface is shallowly concave, although this appears to have been exaggerated by the same distortion that has affected the distal end. The junction between the dorsal and ventrolateral surfaces is more acute than that between the ventrolateral and medial surfaces, although the latter again seems to have been distorted. Despite the fact that the dorsal three-quarters of the distal end have been shifted dorsally relative to the ventral quarter, it appears to have been reniform *in vivo* (figure 27*o*). The distal articular surface of metatarsal IV is approximately perpendicular to the shaft, not medially bevelled as in brachiosaurids [5].

#### 9.5.5. Metatarsal V

Right metatarsal V is virtually complete (figure 27*p–u*), but has suffered slight dorsoventral deformation. In proximal view (figure 27*q*), the proximal end is triangular, with a very shallowly concave ventral margin and somewhat straighter dorsolateral and dorsomedial margins. It is also dorsally domed and dorsoventrally expanded relative to the shaft, contrasting with *Tastavinsaurus*, *Muyelensaurus*, and some saltasaurids, wherein it is unexpanded [38]. The proximal surface is convex in all directions. The mediolateral width of the proximal end of metatarsal V is slightly more than twice that of the distal end—similar to the condition seen among somphospondylans, including the early-branching form *Tastavinsaurus* and the titanosaurs *Epachthosaurus* and *Alamosaurus*, but not as pronounced as in *Rapetosaurus* where the mediolateral width of the proximal end is more than thrice that of the distal end [101]. In dorsal view (figure 27*t*), the proximal margin is shallowly and evenly mediolaterally convex, the medial and lateral margins are proximodistally concave, and the distal margin is broadly rounded. The dorsal surface of metatarsal V is mediolaterally convex along its length. Although the steepness of the convexity diminishes distally, the fact that it has this shape means that metatarsal V lacks true lateral or medial surfaces—each merges smoothly with the dorsal margin. However, the margin between the dorsal and ventral surfaces appears to have been manifested as a ridge on the lateral and medial sides. The ventral surface (figure 27*u*) is essentially flat, although the proximal and distal ends show some sign of wear. There is no evidence of a median tubercle or ridge on the ventral surface, distinguishing AODF 0906 from *Epachthosaurus*, *Mendozasaurus*, and saltasaurines [38]. The distal end is ‘D'-shaped in distal view, with the flat side of the ‘D' being the ventral margin. The distal surface is convex.

### 9.6. Pedal phalanges

**Figure 28.**
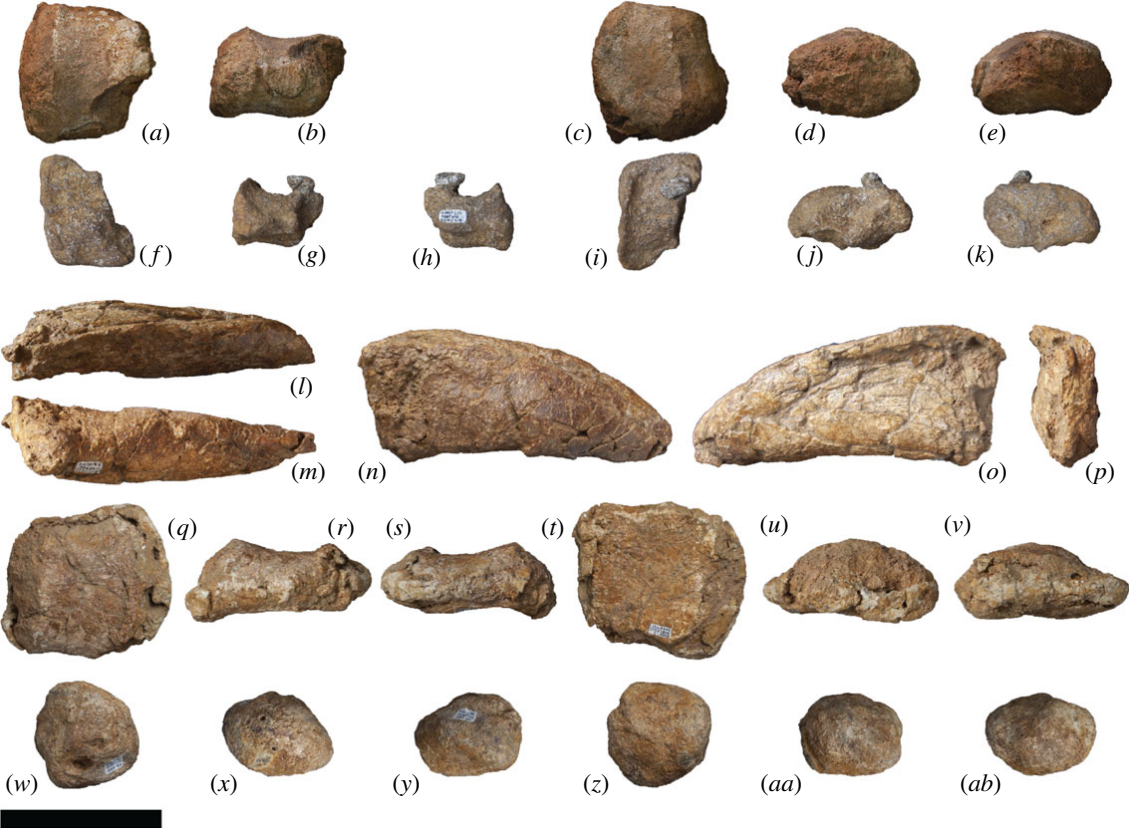
*Diamantinasaurus matildae* referred (AODF 0906) right pedal phalanges. (*a–e*) Right pedal phalanx III-1 in (*a*) dorsal, (*b*) lateral, (*c*) ventral, (*d*) proximal and (*e*) distal views. (*f–k*) Right pedal phalanx III-2 in (*f*) dorsal, (*g*) lateral, (*h*) medial, (*i*) ventral, (*j*) proximal and (*k*) distal views. (*l–p*) Right pedal ungual phalanx III-3 in (*l*) dorsal, (*m*) ventral, (*n*) lateral, (*o*) medial and (*p*) proximal views. (*q–v*) Right pedal phalanx IV-1 in (*q*) dorsal, (*r*) lateral, (*s*) medial, (*t*) ventral, (*u*) proximal and (*v*) distal views. (*w*–*ab*) Right pedal phalanx IV-2 in (*w*) dorsal, (*x*) lateral, (*y*) medial, (*z*) ventral, (*aa*) proximal and (*ab*) distal views. Scale bar = 100 mm.

Five pedal phalanges (including one ungual) are preserved in AODF 0906 (figure 28; table 11). All appear to be from the right foot, and they are interpreted as III-1, III-2, III-3, IV-1 and IV-2 (figure 26*a*). If these interpretations are correct, then digit III had three phalanges, as in brachiosaurids and *Epachthosaurus*, but unlike the early-branching somphospondylan *Gobititan*, the lognkosaurian titanosaurs *Mendozasaurus* and *Notocolossus*, and the saltasaurid titanosaur *Opisthocoelicaudia*, wherein only two phalanges are present in pedal digit III [244,247,248]. In AODF 0906, digit IV had two phalanges, as in neosauropods generally [8,100,106]. Given that all somphospondylans for which pedes are known have two phalanges (one non-ungual and one ungual) on pedal digits I and II, and none on V [2,244,249], we interpret the pedal phalangeal formula for *Diamantinasaurus* as being ?2-?2-3-2-?0.

**Table 11.** Measurements of the right pedal phalanges of AODF 0906 *Diamantinasaurus matildae*. An asterisk (*) indicates a tentative measurement based on an incomplete or distorted specimen.

measurement (mm)	right pedal phalanges				
	III-1	III-2	III-3	IV-1	IV-2
proximodistal length	85	46	162	106	60
maximum proximal mediolateral width	93	65	25	84	60
maximum proximal dorsoventral height	57	41	69	43	46
mid-shaft mediolateral breadth	85	63	—	85	59
maximum distal mediolateral width	81	57	—	87	60
maximum distal dorsoventral height	54	31	—	37	48

#### 9.6.1. Pedal phalanx III-1

Right pedal phalanx I-1 (figure 28*a–e*) is almost complete with the exception of a small proximomedial portion and a larger distolateral one. In proximal view (figure 28*d*), the phalanx is ovate, with the maximum dorsoventral height situated slightly lateral to the midline. The ventral margin of the proximal end is almost flat, whereas the dorsal margin is broadly convex and the lateral and medial margins more acutely so. In dorsal view (figure 28*a*), the phalanx is slightly flared proximally and would presumably have been similarly flared distally when complete. The dorsal surface is proximodistally concave and mediolaterally convex, merges smoothly with the more strongly dorsoventrally convex medial and lateral margins, and preserves a subtle bulge near the medial margin at the mid-length. The ventral surface (figure 28*c*) is shallowly concave, with the most pronounced but narrowest concavity at the distal end, where two subtle condyles are present. In distal view (figure 28*e*), the phalanx would presumably have been broadly ‘D'-shaped when complete (albeit with the ventral margin shallowly concave rather than straight). In lateral (figure 28*b*) and medial views, the distal surface is inclined somewhat proximoventrally–distodorsally. It is divided into subtle lateral and medial condyles, which are divided by a shallow groove that is more pronounced ventrally than dorsally.

#### 9.6.2. Pedal phalanx III-2

The pedal phalanx interpreted here as III-2 (figure 28*f–k*) is incompletely preserved but would have been relatively small, even when complete. In proximal view (figure 28*j*), the phalanx is D-shaped. The dorsal surface (figure 28*f*) is proximodistally concave and mediolaterally convex, with rather acute proximal and distal flanges (visible in lateral (figure 28*g*) and medial (figure 28*h*) views); however, these might be a result of taphonomic distortion, given that this element was found in direct contact with another. The ventral surface is worn and incomplete (figure 28*i*), and the incomplete distal end is D-shaped (figure 28*k*).

#### 9.6.3. Pedal ungual phalanx III-3

Right pedal ungual III-3 is complete but has suffered relatively minor mediolateral crushing (figure 28*l–p*). Relative to its dorsoventral height, it is quite elongate proximodistally, similar to the pedal unguals of some titanosaurs, including *Epachthosaurus*, *Bonatitan*, MUCPv-1533 (La Invernada titanosaur) and *Neuquensaurus* [155,162,247,250], but unlike the relatively proximodistally shorter and dorsoventrally taller unguals of others, including *Bonitasaura*, Lognkosauria, *Malawisaurus*, *Mnyamawamtuka*, *Opisthocoelicaudia* and *Rapetosaurus* [115,225,236,244,251–254]. The ungual in AODF 0906 lacks the strong curvature seen in the pedal unguals of the brachiosaurid *Cedarosaurus* [255] and the non-titanosaurian somphospondylan *Ligabuesaurus* [174]. In proximal view (figure 28*p*), the ungual is significantly taller dorsoventrally than it is wide mediolaterally, with the lateral margin somewhat concave and the medial one essentially straight. The proximal articular surface is bevelled proximolaterally relative to the long axis of the ungual. In lateral (figure 28*n*) and medial views (figure 28*o*), the ungual shows a gradual but distinct distal taper, the proximal margin is straight, the dorsal margin is convex, and the ventral surface is concave. The dorsal surface (figure 28*l*) is manifested as a sharp ridge, although this appears to have been exaggerated somewhat by *post mortem* deformation. The lateral surface (figure 28*n*) is dorsoventrally convex but undulates proximodistally, such that the mid-section is convex and the proximal and distal ends show some degree of concavity. The medial surface (figure 28*o*) is dorsoventrally and proximodistally convex, although the dorsal two-thirds have suffered deformation such that they are now partly concave. The medial surface contributes more to the ventral surface than the lateral one, with the junction between the two manifested as a ridge that more or less follows the lateral margin. Neither the medial nor lateral surface has a vascular groove, differentiating it from several titanosauriform taxa, including *Paludititan nalatzensis* [256]. The lack of vascular grooves also differentiates this ungual from the manual unguals of the *Diamantinasaurus matildae* holotype specimen (AODF 0603) and juvenile referred specimen (AODF 0663) [9,37,44]. The ventral margin appears to show a small tuberosity near the distal end, a feature that characterizes most somphospondylans, including the non-titanosaurians *Euhelopus*, *Tangvayosaurus*, *Tastavinsaurus* and *Gobititan*, and the titanosaurs *Malawisaurus*, *Epachthosaurus*, *Mendozasaurus* and *Muyelensaurus* [38,101,220].

#### 9.6.4. Pedal phalanx IV-1

Pedal phalanx IV-1 is complete but has suffered from slight dorsoventral compression (figure 28*q–v*); nevertheless, it was probably somewhat dorsoventrally compressed in life, as is the case in pedal phalanges IV-1 of *Bonitasaura* [257] and *Mendozasaurus* [236]. In proximal view (figure 28*u*), the phalanx is D-shaped with a flat ventral surface. The dorsal surface (figure 28*q*) is mediolaterally convex and very shallowly proximodistally concave. The lateral and medial margins are shallowly concave proximodistally in dorsal view, and the former has a subtle node at the mid-length. The distal end is convex in dorsal view. The medial (figure 28*s*) and lateral (figure 28*r*) surfaces are acutely dorsoventrally convex. The ventral surface is concave (figure 28*t*). The distal end is slightly crushed, but the distal surface would have been convex. In distal view (figure 28*v*), the phalanx is D-shaped but flattened, with the apex slightly lateral to the midline.

#### 9.6.5. Pedal phalanx IV-2

Pedal phalanx IV-2 is rounded and relatively featureless (figure 28*w*–*ab*). In proximal view (figure 28*aa*), the phalanx is shaped like a tall ‘D', with the flat margin being the ventral one. The proximal surface is proximodorsally–distoventrally inclined, as can be seen in lateral view (figure 28*x*). Whereas the lateral and medial margins are straight in dorsal view, the distal margin is convex. The dorsal surface is proximodistally concave and mediolaterally convex. Both the lateral (figure 28*x*) and medial (figure 28*y*) surfaces are smoothly proximodistally concave, but the lateral is dorsoventrally convex and the medial is dorsoventrally concave (such that it looks almost ‘kinked'). The ventral surface (figure 28*z*) is convex. The distal surface is rounded (figure 28*ab*).

## 10. Phylogenetic results

**Figure 29.**
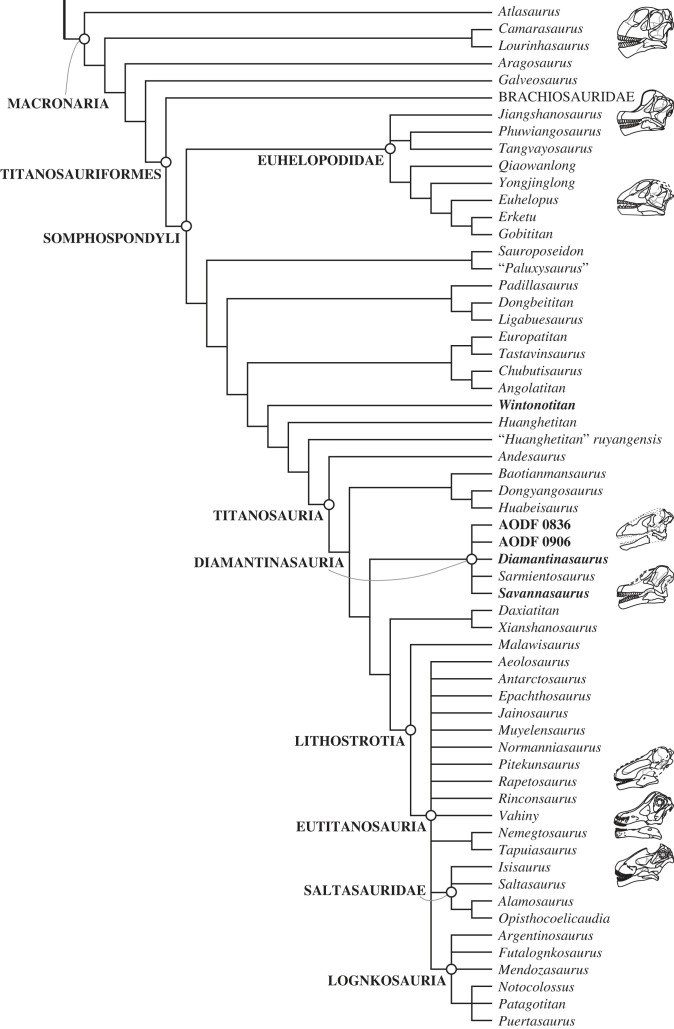
Equal weights phylogenetic analysis. Agreement subtree showing the relationships within Titanosauriformes based on the results of the equal weights phylogenetic analysis. Brachiosauridae has been collapsed into a single lineage. The names of Australian taxa/specimens are in boldface.

Analysis under equal weights results in 300 960 most parsimonious trees (MPTs) of length 2679 steps. The topology (figure 29) is essentially unchanged from that presented in Poropat *et al*. [41]. AODF 0906 is recovered as a member of Diamantinasauria, forming a polytomy with the *Diamantinasaurus matildae* holotype (AODF 0603), AODF 0836, *Savannasaurus* and *Sarmientosaurus*. Diamantinasauria has a Bremer support of 3 and is recovered as a non-lithostrotian titanosaur clade. It would take trees three steps longer to place Diamantinasauria outside of Titanosauria.

**Figure 30.**
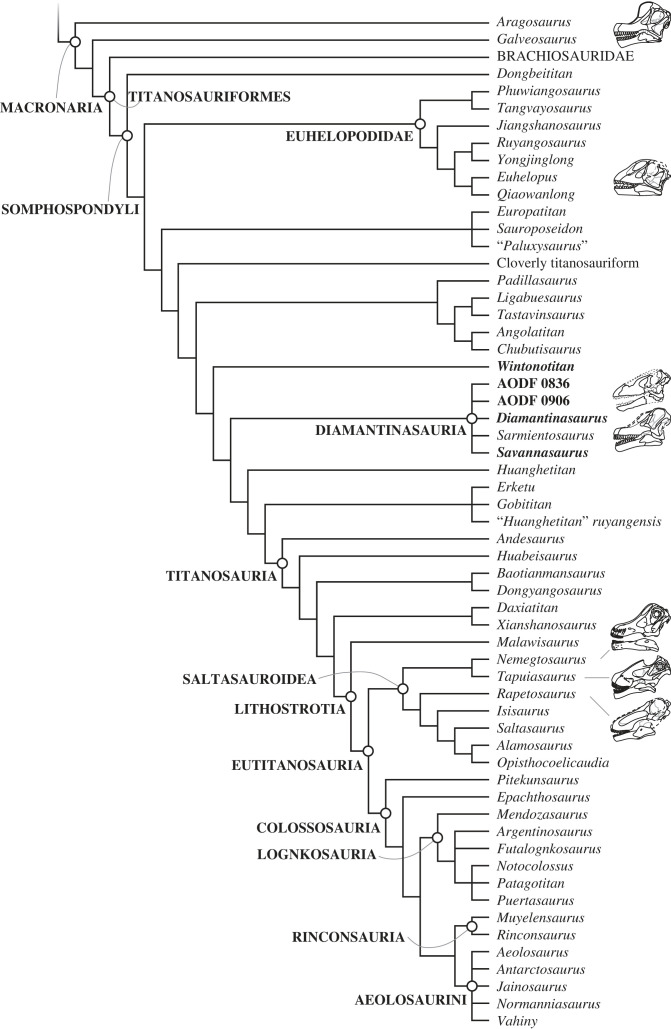
Extended implied weights phylogenetic analysis. Agreement subtree showing the relationships within Titanosauriformes based on the results of the equal weights phylogenetic analysis. Brachiosauridae has been collapsed into a single lineage. The names of Australian taxa/specimens are in boldface.

Analysis using extended implied weights produces 588 MPTs of length 139.9 steps. As with the equal weights analysis, the same five operational taxonomic units (OTUs) form a diamantinasaurian polytomy. The overall topology (figure 30) is little changed from the equivalent analysis in Poropat *et al*. [41]; however, Diamantinasauria is recovered outside of Titanosauria, with a small number of East Asian taxa placed as closer relatives to the titanosaurian radiation (figure 30).

Analyses were also run with AODF 0603, AODF 0836 and AODF 0906 combined as a single OTU. The topology of the trees was identical, but Diamantinasauria remained a polytomy.

## 11. Discussion

### 11.1. The phylogenetic composition and position of Diamantinasauria

As noted by Carballido *et al*. [1], the taxa comprising the clade Diamantinasauria have remained relatively consistent since the initial publication of *Savannasaurus elliottorum* and the referred specimen of *Diamantinasaurus matildae* (AODF 0836) by Poropat *et al*. [38]. *Sarmientosaurus musacchioi* was first resolved within Diamantinasauria by Poropat *et al*. [41], and this result is replicated herein—with additional support from features of the AODF 0906 skull, notably the presence of a posterior tongue-like process on the quadratojugal, which was identified as an autapomorphy of *Sarmientosaurus* by Martínez *et al*. [23], but can now be regarded as a diamantinasaurian synapomorphy. The only other taxa to have been resolved within Diamantinasauria are *Wintonotitan wattsi* and *Australotitan cooperensis*, by Hocknull *et al*. [34], and both of these taxa derive from the same stratigraphic unit as *Diamantinasaurus* and *Savannasaurus* (i.e. the Winton Formation in the Eromanga Basin). However, we note that *Wintonotitan* was only resolved within Diamantinasauria by Hocknull *et al*. [34] when they ran modified analyses in which large numbers of taxa (non-macronarian taxa or post-Cenomanian taxa) or characters (all cranial and axial characters) were excluded. As such, we contend that there is currently limited support for the inclusion of *Wintonotitan* within Diamantinasauria.

Whereas the content of Diamantinasauria has remained fairly consistent, the phylogenetic position of the clade within Somphospondyli has not [1]. Prior to the description of *Savannasaurus elliottorum* [38], *Diamantinasaurus* was generally regarded as a lithostrotian titanosaur, specifically either as a member of Opisthocoelicaudiinae within Saltasauridae [9,37], the sister taxon to Saltasauridae [9,21], or the sister taxon to *Tapuiasaurus* [37]. However, in other analyses it was resolved within Titanosauria but just outside Lithostrotia, or within Somphospondyli but outside Titanosauria [101]. The inclusion of *Savannasaurus elliottorum* and an additional *Diamantinasaurus* specimen (AODF 0836) within an updated and expanded phylogenetic analysis based on that of Mannion *et al*. [101] saw the two *Diamantinasaurus* specimens and *Savannasaurus* consistently resolved as a clade, which was situated near the base of Titanosauria in equal weights analyses, but within Lithostrotia (and close to Saltasauridae) in implied weights analyses [38). Subsequent analyses based on this dataset have generally returned the same result [12,90,236,258–260], with a few notable exceptions [6,90,249,261], as discussed by Poropat *et al*. [40]. An early-branching position for Diamantinasauria within Titanosauria has received additional support in several recent studies [34,40,41,43]. Nevertheless, it is plausible that the presence of several seemingly plesiomorphic titanosaurian characters [40,41] simply indicates that Diamantinasauria occupies a position outside Titanosauria, rather than an early-branching position within, as recovered in our analyses applying extended implied weighting. This interpretation is supported by at least one newly identified character state that would be plesiomorphic within Titanosauria or a slightly more inclusive clade—the presence of five, rather than at least six, sacral vertebrae in *Diamantinasaurus matildae*. We discuss the possible implications of this feature, as well as aspects pertaining to the pes and skull, below.

### 11.2. Implications of the presence of five sacral vertebrae in Diamantinasauria

Currently, *Diamantinasaurus* is the only diamantinasaurian for which we can evaluate the number of sacral vertebrae, with three specimens consistently demonstrating that the sacrum of this genus was composed of only five vertebrae. As noted above, no other titanosaur is characterized by fewer than six sacral vertebrae. By itself, this might merely indicate that five sacral vertebrae is the plesiomorphic condition in titanosaurs, given that we do not know the sacral counts in other early diverging titanosaurs, including *Andesaurus* [262], *Choconsaurus* [159] and *Mnyamawamtuka* [254]. However, as noted above, several somphospondylans that almost certainly lie outside of Titanosauria are characterized by six-vertebrae sacra. Of those somphospondylans that are known to have sacra composed of five vertebrae, most of these taxa (i.e. *Sauroposeidon*, *Sibirotitan* and *Tastavinsaurus*) are typically recovered as relatively early diverging members of the clade (e.g. [219]; this study), which could be interpreted as retention of the plesiomorphic eusauropod condition prior to the acquisition of a sixth sacral vertebra at a more nested node within Somphospondyli. Nevertheless, *Huanghetitan liujiaxiaensis*, which is also characterized by a five-vertebrae sacrum [218], is consistently recovered as one of the closest relatives of Titanosauria in our analyses. Furthermore, more detailed evaluation of somphospondylan interrelationships (figures 29 and 30) indicates that some lineages characterized by six-vertebrae sacra (namely taxa placed within Euhelopodidae) are typically recovered as more distantly related to Titanosauria than some taxa with five-vertebrae sacra (e.g. *Sauroposeidon*). This suggests that there have either been a number of independent acquisitions of a six-vertebrae sacrum among Somphospondyli and/or that there have been numerous reversals to a five-vertebrae sacrum. The fact that six-vertebrae sacra have also been described from non-titanosauriform sauropods—notably, a specimen of the early-branching macronarian *Camarasaurus* [263], the enigmatic neosauropod ‘*Apatosaurus' minimus* [264–266], and the non-neosauropodan eusauropod *Klamelisaurus gobiensis* [267]—further highlights the likely high levels of plasticity of this characteristic. As such, we suggest that the number of sacral vertebrae is of relatively limited importance in determining whether Diamantinasauria lies within or outside of Titanosauria.

### 11.3. Implications of the diamantinasaurian pes for titanosaurian pedal evolution

Several previous studies have discussed the evolution of the titanosauriform pes [2,244,247,249,251,253,268,269]. These authors have noted that there is a trend towards pedal phalangeal reduction in the clade, particularly within Titanosauria (table 12 in appendix). All titanosaurs have only two phalanges (including an ungual) on pedal digits I and II. Somphospondylans that unequivocally lie outside of Titanosauria, and that preserve near-complete pedes, are rare, but *Tangvayosaurus* and *Tastavinsaurus* have both been reconstructed with three phalanges on digit II [239,296]. By contrast, the non-titanosaurian somphospondylan *Gobititan* has only two phalanges on this digit [290]. Most titanosaurs have two phalanges on pedal digit III, including *Mendozasaurus*, *Notocolossus*, *Opisthocoelicaudia*, UNCUYO-LD 313 (Agua del Padrillo titanosaur) and MUCPv-1533 (La Invernada titanosaur). However, some early deriving forms, such as *Epachthosaurus* and (probably) *Diamantinasaurus*, have three phalanges on this digit. Three or more phalanges also characterize digit III of *Tangvayosaurus* and *Tastavinsaurus*, whereas *Gobititan* only has two phalanges on this digit. Nearly all somphospondylans have only two phalanges on digit IV; the only known possible exception is *Opisthocoelicaudia*, which only preserves one phalanx on this digit [225], although it has been inferred to have possessed a second based on the morphology of the distal end of that phalanx [249]. All of the few titanosaurs that preserve near-complete pedes lack phalanges on pedal digit V. *Gobititan*, *Tangvayosaurus* and *Tastavinsaurus* all have one or two phalanges on pedal digit V [239,290,296].

**Table 12.** Cretaceous titanosauriform taxa for which pedal elements are known. Reports only known to the authors from published abstracts have been omitted. L, left; l, lower; m, middle; mt, metatarsal; pp, pedal phalanx; pu, pedal ungual; R, right; u, upper.

taxon	specimen	locality	formation	age range	pedal elements present	phalangeal formula	ungual formula	reference(s)
Lithostrotia indet.	TMM 45890-2	Texas, USA	Black Peaks	u. Maastrichtian	R pu	?	?	Fronimos & Lehman [270]
*Rapetosaurus krausei*	FMNH PR 2209	Mahajanga, Madagascar	Maevarano	Maastrichtian	L mt I–IV, R mt V, four pp (including two pu)	2-?-?-?-?	1-?-?-?-?	Curry Rogers [251]
*Rapetosaurus krausei*	UA 9998	Mahajanga, Madagascar	Maevarano	Maastrichtian	R mt I	?	?	Curry Rogers [251]
Titanosauria indet. (formerly ?*Antarctosaurus* sp.)	GSI K27/509	Maharashtra, India	Lameta	Maastrichtian	L mt I	?	?	Huene & Matley [271]
*Alamosaurus sanjuanensis*	TMM 43621 1	Texas, USA	Black Peaks	Maastrichtian	Mt	?	?	Lehman & Coulson [272]
*Alamosaurus sanjuanensis*	TMM 42495	Texas, USA	Javelina / Black Peaks	Maastrichtian	Mt	?	?	Lehman & Coulson [272]
*Alamosaurus sanjuanensis*	TMM 43598	Texas, USA	Javelina / Black Peaks	Maastrichtian	Mt II	?	?	Woodward & Lehman [273]
?*Alamosaurus sanjuanensis*	NMMNH P-49967	New Mexico, USA	Ojo Alamo	Maastrichtian	R mt I–V, five R pp	?	?	D'Emic *et al.* [245]
*Paludititan nalatzensis*	UBB NVM1	Haţeg Basin, Romania	Sânpetru	Maastrichtian	two pu	?	?	Csiki *et al*. [256]
*Opisthocoelicaudia skarzynskii*	Z. PAL MgD-I/48	Ömnögovi, Mongolia	Nemegt	Maastrichtian	R mt I–V, L mt I–II and IV, seven R pp (including three pu)	2-2-2-1-0 [225]	1-1-1-0-0	Borsuk-Białynicka [225]
						2-2-2-2-0 (inferred by González Riga *et al*., [249] on the basis of the morphology of pp IV-1)		
*Saltasaurus loricatus*	PVL 4017 various	Salta, Argentina	Lecho	?Campanian–Maastrichtian	seven mt (from more than one individual)	?	?	Powell [144,145]; S.F.P. and P.D.M. *pers. obs.* 2013
*Aeolosaurus* sp.	MPCA-Pv 27178	Río Negro, Argentina	Allen	Campanian–Maastrichtian	mt I	?	?	García & Salgado [274]
*Bonatitan reigi*	‘Individual A’ (MACN PV RN 821, 1061 and unnumbered)	Río Negro, Argentina	Allen	Campanian–Maastrichtian	L mt I, R mt III, mt V	?	?	Martinelli & Forasiepi [146]; Salgado *et al*. [155]; S.F.P. and P.D.M. *pers. obs.* 2013
*Bonatitan reigi*	‘Individual D’ (MACN PV RN 821, 1061 and unnumbered)	Río Negro, Argentina	Allen	Campanian–Maastrichtian	mt I, III (×2), V (×2), pu	?	?	Martinelli & Forasiepi [146]; Salgado *et al*. [155]; S.F.P. and P.D.M. *pers. obs.* 2013
Titanosauria indet.	MUCPv-1533 (‘La Invernada titanosaur’)	Neuquén, Argentina	Allen	Campanian–Maastrichtian	L mt I–V, eight pp (including three pu)	2-2-2-2-0	1-1-1-0-0	González Riga *et al*. [247,253]
Lithostrotia indet. (formerly *Laplatasaurus araukanicus*)	MLP CS 1203, 1217, 2002	Río Negro, Argentina	Allen	Campanian–Maastrichtian	R mt II and ?V, L pu (possibly from more than one individual)	?	?	Huene [162]; Gallina & Otero [275]
*Dreadnoughtus schrani*	MPM PV 1156	Santa Cruz, Argentina	Cerro Fortaleza	Campanian–Maastrichtian	R mt I–II, R pu I-2	?	?	Lacovara *et al*. [252]; Ullmann & Lacovara [276]
*Ampelosaurus atacis*	MDE C3 various	Languedoc-Roussillon, France	Marnes Rouges Inférieures	Campanian–Maastrichtian	mt, pp	?	?	Le Loeuff [168,277]
*Lirainosaurus astibiae*	MCNA 14474	Alava, Spain	Vitoria	u. Campanian–l. Maastrichtian	L mt III	?	?	Díez Díaz *et al*. [278]
*Aeolosaurus* sp.	MPCA-Pv 27100	Río Negro, Argentina	Los Alamitos	u. Campanian–l. Maastrichtian	L mt I–IV	?	?	Salgado *et al*. [97]
Lithostrotia indet.	UNPSJB-PV 1051	Chubut, Argentina	Lago Colhué Huapi	Campanian–?Maastrichtian	two mt and one pp	?	?	Ibiricu *et al*. [279]
*Brasilotitan nemophagus*	MPM 125R	São Paulo, Brazil	Adamantina	u. Campanian	pu	?	?	Machado *et al*. [164]
*Atsinganosaurus velauciensis*	MMS/VBN.93.10	Bouches du Rhône, France	`Begudian’ sandstones	u. Campanian	L mt I	?	?	Garcia *et al*. [280]; Díez Díaz *et al*. [13]
*Mansourasaurus shahinae*	MUVP 2000	Dakhla Oasis, Egypt	Quseir	Campanian	R mt I, II or IV, and III	?	?	Sallam *et al*. [160]
*Antarctosaurus wichmannianus*	MACN 6904	Río Negro, Argentina	Anacleto	l. Campanian	L mt I–IV (and V?)	?	?	Huene [162]; Powell [145]
cf. *Bonitasaura* sp. (formerly *Laplatasaurus araukanicus*)	MLP Av. 2102, 2111, 2113	Neuquén, Argentina	Anacleto	l. Campanian	L mt I, R mt III–IV (possibly from more than one individual)	?	?	Huene [162]; Gallina & Otero [275]
*Neuquensaurus australis* (including *N. robustus*)	MLP CS various	Río Negro, Argentina	Anacleto	l. Campanian	mt I (×3), II (×4), III (×3) IV (×4) and V (×4), seven pp (including two pu) (from more than one individual)	?	?	Huene [162]; Powell [145]; Otero [240]
*Neuquensaurus australis*	MCS 10	Río Negro, Argentina	Anacleto	l. Campanian	one mt	?	?	Salgado *et al*. [226]; Otero [240]
Lithostrotia indet.	MACN	Río Negro, Argentina	Anacleto	l. Campanian	two mt	?	?	Huene [162]; Gallina & Otero [275]
*Bonitasaura salgadoi*	MPCA-Pv 460	Río Negro, Argentina	Bajo de la Carpa	Santonian	L mt I–III and V, R mt I–II and IV–V, three L pp (including one pu)	?	?	Apesteguía [163]; Gallina and Apesteguía [257]
*Notocolossus gonzalezparejasi*	UNCUYO-LD 302	Mendoza, Argentina	Plottier	u. Coniacian–l. Santonian	R mt I–V, eight pp (including three pu)	2-2-2-2-0	1-1-1-0-0	González Riga *et al*. [244,253]
Titanosauria indet.	UNCUYO-LD 313 (Agua del Padrillo titanosaur)	Mendoza, Argentina	Plottier	u. Coniacian–l. Santonian	L mt I–V, eight pp (including three pu)	2-2-2-2-0	1-1-1-0-0	González Riga *et al*. [244,253]
*Kaijutitan maui*	MAU-Pv-CM-522	Neuquén, Argentina	Sierra Barossa	u. Coniacian	R mt II	?	?	Filippi *et al*. [281]
*Mendozasaurus neguyelap*	IANIGLA-PV 77–79, 100, 153	Mendoza, Argentina	Sierra Barossa	m.–u. Coniacian	mt I (×3), II, III (×2), IV (×2) and V (×4), 10 pp (including three pu) (from more than one individual)	?	?	González Riga [236,282]
Saltasauridae indet.	FC-DPV 1900	Paysandú, Uruguay	Guichón	Turonian–Santonian	several mt and pp	?	?	Soto *et al*. [283]
*Muyelensaurus pecheni*	MRS Pv various	Neuquén, Argentina	Portezuelo	m.–u. Turonian	15 mt and 11 pp (from more than one individual)	?	?	Calvo *et al.* [116]
Titanosauria indet.	IZANUZ 27, 248 and 636; ZIN PH 672/16	Navoi, Uzbekistan	Bissekty	Turonian	mt I (×2) and II (×2) (probably from more than one individual)	?	?	Sues *et al*. [284]
*Epachthosaurus sciuttoi*	UNPSJB-PV 920	Chubut, Argentina	Lower Bajo Barreal	u. Cenomanian–l. Turonian	L and R mt I–V, 18 pp (including six pu)	2-2-3-2-0	1-1-1-0-0	Martínez *et al*. [250]; S.F.P. and P.D.M. *pers. obs.* 2013
*Savannasaurus elliottorum*	AODF 0660	Queensland, Australia	Winton	Cenomanian	L mt III	?	?	Poropat *et al*. [38,40]
*Diamantinasaurus matildae*	AODF 0906	Queensland, Australia	Winton	Cenomanian	R mt I–V, five R pp (including one pu)	?-?-3-2-0	?-?-1-0-0	This paper
Titanosauriformes indet. (=‘*Acanthopholis*’ *platypus*)	CAMSM B55449–55461	Cambridgeshire, United Kingdom	Upper Greensand	l. Cenomanian	L mt I–V and one pp	?	?	Seeley [285–287]); Le Loeuff [288]
*Sonorasaurus thompsoni*	ASDM 500	Arizona, USA	Turney Ranch	u. Albian–l. Cenomanian	R mt I–II and IV–V, six pp (including two pu)	?	?	Ratkevich [289]; D'Emic *et al*. [246]
*Gobititan shenzhouensis*	IVPP 12579	Gansu, China	Xinminbao Group	l.–m. Albian	L mt I–V, 10 pp (including three pu)	2-2-2-2-2	1-1-1-0-0	You *et al*. [290]
*Ligabuesaurus leanzai*	MCF-PVPH-233	Neuquén, Argentina	Lohan Cura	l. Albian	R mt I–V, R pp I-1–2 and II-1 (including one pu)	2-?-?-?-?	1-?-?-?-?-?	Bonaparte *et al*. [291]; Bellardini *et al*. [174]
*Cedarosaurus weiskopfae* (=*Pleurocoelus* sp.)	FMNH PR 977	Texas, USA	Paluxy	l. Albian	L mt I–V, 11 L pp (including four pu)	2-3-4-2-0 [292] 2-3-3-3-1 [293]	1-1-1-1-0	Langston [294]; Gallup [292]; D'Emic [293]
*Mnyamawamtuka moyowamkia*	RRBP 05834	Rukwa Rift Basin, Tanzania	Galula	Aptian–Cenomanian	L mt I–II and IV–V, R mt III, three pp (including two pu)	?	?	Gorscak & O'Connor [254]
*Sauroposeidon proteles* (=*Paluxysaurus jonesi*)	FWMSH 93B-10-16	Texas, USA	Twin Mountains	Aptian–Albian	R mt I and II	?	?	Rose [175]; D'Emic [293]
*Sauroposeidon proteles* (=*Paluxysaurus jonesi*)	FWMSH 93B-10-26	Texas, USA	Twin Mountains	Aptian–Albian	?L mt IV	?	?	Rose [175]; D'Emic [293]
*Chubutisaurus insignis*	MPEF-PV 1129	Chubut, Argentina	Cerro Barcino	u. Aptian–l. Albian	L mt IV	?	?	Carballido *et al*. [243]
*Tapuiasaurus macedoi*	MZSP-PV 807	Minas Gerais, Brazil	Quiricó	Aptian	L mt I–V, several pp	2-2-2-1-1	1-1-1-0-0	Zaher *et al*. [21]; Navarro [295]
*Malawisaurus dixeyi*	MAL 145	Dinosaur Beds	Karonga, Malawi	Aptian	?mt III	?	?	Gomani [115]
*Malawisaurus dixeyi*	MAL 210–213	Dinosaur Beds	Karonga, Malawi	Aptian	R mt V, R pp I-1, L pu I-2 and II-2	?	?	Gomani [115]
*Tangvayosaurus hoffeti*	TV2-1–40	Savannakhet, Laos	Grès supérieurs	Aptian	L mt I–III, nine pp (including three pu)	2-3-3-2-1	1-1-1-0-0	Allain *et al*. [296]
Titanosauriformes indet. (=*Astrodon johnstoni*, =*Pleurocoelus altus*, =*P. nanus*)	USNM various	Maryland, USA	Arundel	l. Aptian	six mt and 13 pp (only one positively identified as pedal)	?	?	Marsh [297]; Lull [298]; Carpenter & Tidwell [123]
*Tastavinsaurus sanzi*	MPZ 99/9	Teruel, Spain	Xert	l. Aptian	R mt I–V, L mt IV, seven pp (including four pu)	2-3-3?-2?-1?	1-1-1-0-0	Canudo *et al*. [220]; Royo-Torres [221]
*Tastavinsaurus sanzi*	CPT- various	Teruel, Spain	Forcall	l. Aptian	L mt I–V, 12 L pp (including three pu)	2-3-4-2-1	1-1-1-0-0	Royo-Torres *et al*. [239]
*Venenosaurus dicrocei*	DMNH 40932	Utah, USA	Cedar Mountain (Poison Strip Member)	Barremian–l. Aptian	R mt I–II and IV	?	?	Tidwell *et al*. [299]; D'Emic *et al*. [246]
*Phuwiangosaurus sirindhornae*	SM K11-0001–0168	Khon Kaen, Thailand	Sao Khua	Barremian	three ?mt, two phalanges (might be manual)	?	?	Suteethorn *et al*. Suteethorn *et al*. [149]
*Dongbeititan dongi*	DNHM D2867	Liaoning, China	Yixian (Jianshangou Member)	Barremian	L mt I–V, pu I and II	?	?	Wang *et al*. [300]
*Fukuititan nipponensis*	FPDM-V8468	Fukui, Japan	Kitadani	Barremian	pp	?	?	Azuma & Shibata [301]
*Cedarosaurus weiskopfae*	DMNH 39045	Utah, USA	Cedar Mountain (Yellow Cat Member)	Valanginian	right mt I–II and V and four R pp (including three pu)	?	1-1-1-?-?	Tidwell *et al*. [255]; D'Emic *et al*. [246]
*Euhelopus zdanskyi*	PMU 24706	Shandong, China	Mengyin	Berriasian	R mt I–IV, five R pp (including one definite and one possible pu)	?	?	Wiman [111]; Wilson & Upchurch [113]

If the above interpretation of the pedal phalanges of AODF 0906 is correct, and irrespective of whether Diamantinasauria occupies an early-branching position within Titanosauria, or lies just outside of Titanosauria, then *Diamantinasaurus* seemingly fits neatly into the pattern of phalangeal reduction seen within Somphospondyli. Nevertheless, more specimens of taxa just outside of Titanosauria, as well as early members of this clade, are required to test this, including whether phalangeal number might be relatively plastic.

### 11.4. Implications of the diamantinasaurian skull for titanosaurian cranial evolution

The evolution of titanosaurian sauropod skull disparity is poorly understood. Few taxa are known from partial or complete skulls, and those that are date either to the late Early Cretaceous (Aptian–Albian: *Tapuiasaurus* [21,22] and *Malawisaurus* [115,120,302]), the early Late Cretaceous (Cenomanian: *Sarmientosaurus* [23]), or the latest Cretaceous (Maastrichtian: *Nemegtosaurus* [18,20] and *Rapetosaurus* [16,17]). The absence of titanosaur skull material prior to the Aptian and relative scarcity from the Turonian–Campanian interval—the incomplete crania of *Antarctosaurus* [145,162], *Bonitasaura* [141] and *Quaesitosaurus* [19], and the embryonic skulls from Auca Mahuevo [129,130] notwithstanding—remains the greatest impediment to understanding titanosaurian skull evolution.

The skulls of early-branching titanosaurs, typified by *Diamantinasaurus* and *Sarmientosaurus* [23], are quite different from those of phylogenetically nested titanosaurs [17,20–22]. The skulls of diamantinasaurians have a more undulatory lateral profile (with the back of the skull taller and somewhat raised relative to the snout), more robust dentigerous elements, and compressed-cone–chisel-like teeth. In all of these ways, the skulls of these early-branching titanosaurs are similar to those of brachiosaurids [24–26,133]. Current evidence implies that diamantinasaurians, as well as other early-branching titanosaurs, such as *Choconsaurus* [159], had rounded jaws, similar to those of *Camarasaurus* [118] and brachiosaurids [24–26].

More phylogenetically nested titanosaurs have elongate, low skulls with pencil- or chisel-like teeth confined to the anterior part of the snout [17,20–22]. In all of these ways, their skulls are reminiscent of those of diplodocoids [161,303], so much so that the skulls of the titanosaurs *Antarctosaurus*, *Nemegtosaurus* and *Quaesitosaurus* were once thought to be from diplodocoids [18,19,100,106,162,304]. However, the titanosaurian affinities of these skulls were demonstrated beyond reasonable doubt through a detailed reappraisal of the specimens [20,305], and confirmed through the subsequent discovery of titanosaurian postcrania at the *Nemegtosaurus* type site [306]. In the interim, the discovery of near-complete skulls associated with undisputed titanosaurian postcranial remains (all attributed to *Rapetosaurus*) settled the issue [3,17].

Among ‘derived' titanosaurs, there is evidence for two main jaw shapes: jaws with rounded ends, like those of *Ampelosaurus*, *Karongasaurus*, *Malawisaurus*, *Mansourasaurus*, *Nemegtosaurus*, *Quaesitosaurus*, *Rapetosaurus* and *Tapuiasaurus* [17–22,115,160,168]; and squared-off jaws, like those of *Antarctosaurus*, *Baalsaurus*, *Bonitasaura* and *Brasilotitan* [141,145,162–165]. This disparity parallels that seen in diplodocoids [105]: some have rounded jaws, such as the dicraeosaurids *Suuwassea emilieae* and *Dicraeosaurus hansemanni* [25,166]; some have squared-off jaws, such as the diplodocids *Apatosaurus louisae*, *Diplodocus longus* and *Tornieria africana*, and the rebbachisaurid *Lavocatisaurus agrioensis* [25,213,303,307]; and others have squared-off and transversely expanded jaws, namely the rebbachisaurid *Nigersaurus* [110,308,309].

Whereas several brachiosaurids are known from skulls of varying levels of completeness (e.g. ‘*Brachiosaurus*' [24], *Giraffatitan* [25], *Abydosaurus* [26]), few non-titanosaurian somphospondylan taxa are represented by any cranial remains at all. Several non-titanosaurian somphospondylans preserve only teeth, including *Astrophocaudia slaughteri* [293], *Borealosaurus wimani* [310], *Europatitan eastwoodi* [311], *Huabeisaurus allocotus* [223,312], *Sibirotitan astrosacralis* [219] and *Yongjinglong datangi* [173]. *Ligabuesaurus leanzai* preserves teeth and a fragmentary maxilla [174,291,313], whereas teeth and a braincase are preserved in both *Mongolosaurus haplodon* [152,314] and *Tambatitanis amicitiae* [151,315]. Teeth are preserved in several specimens of *Phuwiangosaurus sirindhornae*, with one specimen also preserving a partial skull [82,149,215,316–319]. *Euhelopus zdanskyi* preserves a nearly complete skull, along with teeth, but lacks the braincase [111–114,320]; furthermore, it is possible that it lies outside Neosauropoda [267]. By far the most complete non-titanosaurian somphospondylan skull is the holotype specimen of *Liaoningotitan sinensis*, although this has only been briefly described [321]. This skull has suffered substantial taphonomic distortion, but the dentaries are effectively mirror images of each other, implying that they probably reflect their original morphology; if so, then *Liaoningotitan* had jaws with somewhat squared-off ends, paralleling some diplodocids, rebbachisaurids, and some titanosaurs, and implying even greater plasticity in sauropod jaw morphology.

**Figure 31.**
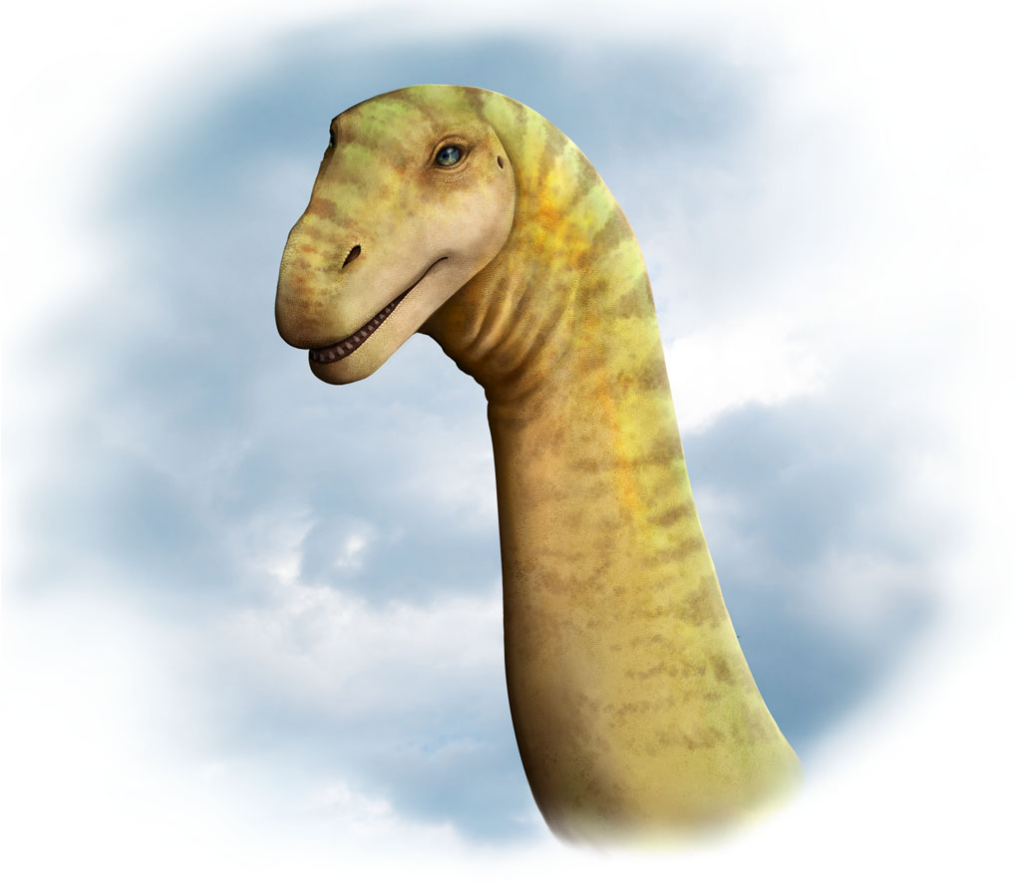
Life restoration of the head and neck of *Diamantinasaurus matildae*, based on AODF 0906 (referred skull) and AODF 0836 (referred partial skull and anterior cervical vertebrae). Artwork © Elena Marian.

The similarity between the skulls of the diamantinasaurians *Diamantinasaurus* and *Sarmientosaurus* implies morphological conservatism in this clade of early-branching titanosaurs (figure 31), starkly contrasting with the disparity seen in the snout shapes of more phylogenetically nested titanosaurs. The similarities between the skulls of *Diamantinasaurus* and *Sarmientosaurus* and those of brachiosaurids are quite striking, as are the differences they present when compared with the skulls of the non-titanosaurian somphospondylan *Liaoningotitan*, and those of other titanosaurs, including the stratigraphically older *Tapuiasaurus* and *Malawisaurus*. Although the disparity in maxillary and dentary alveolus count (nine maxilla, 11 dentary in *Diamantinasaurus*; 11 or 12 maxilla, 13 dentary in *Sarmientosaurus*), and the differing number of ossified exits on each side for cranial nerves V and XII (two for CN V, one for CN XII in *Diamantinasaurus*; three for CN V, two for CN XII in *Sarmientosaurus*) indicate some disparity between the two taxa, a similar level of disparity has been identified within some sauropod genera. *Camarasaurus*—one of the few sauropod genera known from multiple skulls—shows variability in all but one of these characters: maxillary alveolus count ranges from 8 to 10 [118], dentary alveolus count ranges from 12 to 13 [118], and either one or two exits for CN XII can be present on each side [118,138,153,154].

The relative conservatism seen in the skulls of diamantinasaurian titanosaurs might be a reflection of the restricted spatio-temporal range of the clade (as presently understood). Both *Sarmientosaurus* and *Diamantinasaurus* lived at approximately the same palaeolatitude (approx. 50° S for *Sarmientosaurus*, approx. 48° S for *Diamantinasaurus* [322]) during the Cenomanian–Turonian, which is regarded as the warmest interval the Earth has endured in the past 150 Myr [323]. By contrast, *Tapuiasaurus* and *Malawisaurus*, which are geologically older (Aptian), but more phylogenetically nested than Diamantinasauria, were discovered at sites that during the Aptian would have been at approximately 15° S and approximately 30° S, respectively [322].

Recent work on Cenomanian–Turonian floras in South America and Zealandia [324] has strongly supported the hypothesis that several, largely latitudinally controlled floral provinces existed in Gondwana during the early Late Cretaceous [325]. The latitudinal band at 50° S was dominated by gymnosperms in both South America and Australia; however, whereas cheirolepidacean gymnosperms were dominant in the former region (constituting more than 80% of the samples studied), gymnosperms other than cheirolepidaceans were most abundant in northern Australia (although cheirolepidaceans still comprised approx. 25% of the flora), and angiosperms had become a notable component of the flora as well [324]. The plant macrofossil record of the Winton Formation indicates that gymnosperms and angiosperms were co-dominant [326]; the findings of Santamarina *et al*. [324] corroborate this. Given that diamantinasaurians have been interpreted as mid-level feeders (*sensu* Whitlock [105]) based on tooth wear analyses [43,327], and given the variety of plants available within the 1–10 m height bracket to diamantinasaurians in northeast Australia (summarized in [43]), it is tempting to attribute at least some of the subtle differences between the skulls of *Sarmientosaurus* and *Diamantinasaurus*—namely those related to alveolus count and tooth morphology—to subtle differences in their diets. The marked difference between the skulls of diamantinasaurians and more derived titanosaurs presumably reflects a greater degree of dietary divergence, but this requires further testing.

## 12. Conclusion

The new specimen of the sauropod dinosaur *Diamantinasaurus matildae* described herein preserves numerous skeletal elements not previously known for this taxon, primarily a partial skull and an incomplete pes. When this new specimen is considered in conjunction with the holotype specimen (presacral vertebrae, sacrum, pectoral girdle, forelimbs, pelvic girdle, right hind limb), and the two previously referred specimens AODF 0836 (partial skull, presacral vertebrae, appendicular elements) and AODF 663 (presacral vertebrae, appendicular elements), the osteology of *Diamantinasaurus* is now among the most completely known of any somphospondylan.

The skull of *Diamantinasaurus matildae* is herein shown to be strikingly similar to that of the approximately coeval *Sarmientosaurus musacchioi* from Argentina. This supports our previous proposal [41] of a close evolutionary relationship between these taxa (forming the clade Diamantinasauria), and is borne out with renewed support in the revised phylogenetic analyses presented herein. Although the position of Diamantinasauria within Somphospondyli remains uncertain, our analyses imply that it lies near the base of Titanosauria: probably just within (meaning that diamantinasaurians were early-branching titanosaurs), or possibly just outside (meaning they were non-titanosaurian somphospondylans), that clade. Future discoveries in the Winton Formation will hopefully fill the remaining gaps in our knowledge of the skeleton of *Diamantinasaurus matildae*, thereby enhancing its importance for future research into the early evolution of Titanosauria.

## Data Availability

The electronic supplementary material, comprising three-dimensional models (derived from surface scans) of the AODF 0906 skull, hindquarters and right pes, three-dimensional models (derived from surface scans) of the AODF 0836 skull, and the CT and Synchrotron data of the AODF 0906 skull elements, is available from MorphoSource: https://www.morphosource.org/projects/000486235. The data are provided in electronic supplementary material [328].

## Appendix

Characters 1–548 (and their scorings) follow those of Mannion *et al*. [90], and those for C549–552 follow those of Poropat *et al*. [41], except where stated below. However, C550, as proposed by Poropat *et al*. [41], was incorrectly worded. This character statement is modified as follows (note that the scores are unaffected):

C550. Cranial nerve V, number of external openings: two or more on each side (0); one on each side (1) ([23,41]; modified here).

We also add the following new characters (C### denotes the character number):

C553: postorbital, lateral surface hosts nutrient foramen near the junction of the squamosal and jugal processes: absent (0); present (1) (new character).
C554: squamosal, quadrate facet is: undivided (0); divided such that it is bilobate or trilobate (1) (new character).
C555: quadratojugal, posterior tongue-like process: absent or weakly developed (0); present (1).
C556: quadratojugal and quadrate, horizontal ridge present across both elements anterior to their articulation point (lateral surface of quadrate, medial surface of quadratojugal: absent (0); present (1) (new character).

Several character scores were changed in existing OTUs. Below, C### denotes the character number, the first value in the parentheses (before the arrow) indicates the original score, and the second value (after the arrow) in the parentheses denotes the new score; sources external to this paper are cited where necessary.

*Diamantinasaurus matildae* AODF 0603: C24 (? → 0); C108 (? → 1; [43]); C109 (? → 0&1; [43]); C110 (? → 0; [43]); C111 (? → 1; [43]); C113 (? → 1; [43]); C173 (0 → 1); C221 (? → 0; [41]); C222 (? → 0; [41]); C260 (0 → 1); C321 (? → 1; [43]); C347 (? → 0); C402 (? → 0; [43]); C408 (? → 1); C454 (? → 0; [43]); C513 (? → 1; [44]); C546 (? → 0; [43]).

*Diamantinasaurus matildae* AODF 0836 (including AODF 2298 [tooth]): C108 (? → 1; [43]); C173 (1 → ?); C321 (? → 1; [43]); C402 (? → 0; [43]); C454 (? → 0; [43]); C546 (? → 0; [43]).

*Sarmientosaurus musacchioi*: C76 (0 → ?); C435 (1 → 0).

*Phuwiangosaurus sirindhornae*: C315 (? → 1; [149]); C316 (0 → 1; [82]); C317 (1 → 0; [82]); C550 (1 → 0; [82]); C551 (? → 1; [82]).
